# Learning brain dynamics across distinct scaling regimes reveals psychiatric signatures

**DOI:** 10.1038/s42003-026-10011-7

**Published:** 2026-05-08

**Authors:** Sangyoon Bae, Junbeom Kwon, Shinjae Yoo, Jiook Cha

**Affiliations:** 1 Interdisciplinary Program in Artificial Intelligence, Seoul National University, Seoul, South Korea; 2 Department of Psychology, University of Texas at Austin, Austin, TX USA; 3 Computational Science Initiative, Brookhaven National Laboratory, Shirley, NY USA; 4 Department of Psychology, Seoul National University, Seoul, South Korea; 5 Department of Brain and Cognitive Sciences, Seoul National University, Seoul, South Korea

**Keywords:** Learning algorithms, Computational models

## Abstract

Understanding how the brain’s nonlinear dynamics give rise to cognition remains a central challenge in neuroscience. Conventional neuroimaging methods assume linearity and stationarity, failing to capture frequency-specific neural computations. We introduce Multi-Band Brain Net (MBBN), a transformer-based framework that models frequency-specific spatiotemporal brain dynamics from fMRI. MBBN integrates biologically grounded frequency decomposition with multi-band self-attention, enabling discovery of frequency-dependent network interactions. Trained on 49,673 individuals across three large-scale cohorts (UK Biobank, Adolescent Brain Cognitive Development Study (ABCD), Autism Brain Imaging Data Exchange (ABIDE)), MBBN achieves state-of-the-art performance in predicting psychiatric and cognitive outcomes—including major depressive disorder (MDD), attention-deficit/hyperactivity disorder (ADHD), and autism spectrum disorder (ASD)—with AUROC improvements of up to 41.36% alongside strong cognitive intelligence prediction. Frequency-resolved analyses reveal disorder-specific signatures: in ADHD, high-frequency fronto-sensorimotor connectivity is attenuated and opercular somatosensory nodes emerge as dynamic hubs; in ASD, orbitofrontal-somatosensory circuits show focal high-frequency disruption alongside enhanced ultra-low-frequency coupling between the temporo-parietal junction and prefrontal cortex. By combining biologically informed frequency decomposition with transformer architecture, MBBN delivers interpretable biomarkers and improved prediction of psychiatric and cognitive traits.

## Abstract (web summary)

A transformer-based framework decomposes fMRI signals into biologically informed frequency bands, revealing frequency-specific brain connectivity patterns that improve prediction of cognitive traits and psychiatric disorders across large cohorts.

## Introduction

Understanding the brain’s dynamic processes is essential for unraveling the mechanisms underlying adaptive cognition and behavior. These processes influence critical functions such as learning, decision-making, and social interactions, with mounting evidence that their dysregulation is closely linked to mental health conditions^1–4^. While functional magnetic resonance imaging (fMRI) has been instrumental in probing brain activity, many conventional analytical methods remain constrained by assumptions of linearity, stationarity and static connectivity. Approaches ranging from mass-univariate statistical tests and seed-based correlations to static functional connectivity maps often treat the brain as a relatively fixed system, thus failing to capture the temporal fluctuations, nonlinear interactions, and adaptive reconfigurations that characterize real neural dynamics. Recent advances in artificial intelligence, particularly transformer-based architectures originally designed for sequential data, have shown promise for modeling brain activity as a continuously evolving network of interdependent processes. Yet, these approaches largely ignore the frequency-specific nature of neural oscillations^5–8^.

This oversight is particularly problematic given that existing neuroimaging models, including recent deep learning approaches, treat brain signals as broadband phenomena, overlooking the fundamental frequency-specific organization of neural computation. Indeed, this organization is best understood through two fundamental properties that describe the brain’s complexity: its multifractal structure and its scale-free dynamics.

Multifractality, a key structural property, points to the brain’s nested, hierarchical organization where multiple scaling dimensions and power-law behaviors coexist^9–13^. Crucially, it is within this complex structure that scale-free dynamics-a key functional property-become apparent. While a broadband signal is not inherently scale-free, decomposing it into distinct frequency components reveals power-law relationships (equation (1)) that signify consistent activity patterns across different timescales. These dynamics play crucial roles in brain maturation and state transitions, with temporal variability serving as a key regulatory mechanism^14,15^. The power-law exponent (*β*) varies across brain regions and states, providing valuable insights into neural network heterogeneity and serving as a potential marker for neurological and psychiatric disorders^16,17^. Evidence from fMRI, ECoG, and EEG studies further suggests that these principles drive frequency-specific changes in connectivity and topological organization, such as small-worldness and modularity^18,19^. Understanding these patterns is crucial for developing more effective diagnostic and therapeutic strategies.

To address these challenges, we introduce Multi-Band Brain Net (MBBN), a transformer-based framework specifically designed to model frequency-resolved spatiotemporal brain dynamics from fMRI. Unlike existing approaches that analyze broadband signals, MBBN explicitly decomposes neural activity into biologically meaningful frequency bands using scale-free principles, then applies specialized attention mechanisms to capture frequency-specific connectivity patterns, incorporating network theory concepts like communicability. MBBN divides fMRI BOLD signals into distinct frequency bands, enabling the capture of band-specific functional and topological properties. This approach provides a neurobiologically informed framework for modeling the brain’s multifractal and scale-free dynamics. The model features two key strengths: Firstly, its self-attention weighted connectivity dynamically models functional connectivity by capturing complex, nonlinear interactions and time-varying relationships between brain regions, thereby overcoming the limitations of traditional static, correlation-based linear representations. Secondly, a communicability-inspired pretraining loss function optimizes for biologically meaningful connectivity patterns. By leveraging these advances, MBBN not only provides deeper insights into brain function but also identifies frequency-specific markers for neurological and psychiatric disorders, offering a powerful new tool for clinical neuroscience research and applications.

1 $$log(power)\propto -\beta \cdot log( \, frequency)$$

## Results

### Experimental setup and downstream tasks

To rigorously evaluate the performance and generalizability of MBBN, we designed a diverse set of predictive tasks spanning biological, cognitive, and clinical domains across three large-scale cohorts (UKB, ABCD, and ABIDE).

The tasks were defined as follows. For biological phenotypes, we performed sex classification. For cognitive function, we predicted Fluid Intelligence scores derived from the NIH Toolbox. In the clinical domain, we predicted a variety of psychiatric outcomes: (1) Major Depressive Disorder (MDD) diagnosis in the ABCD dataset (classification), (2) Patient Health Questionnaire-9 (PHQ-9) MDD scores in the UKB dataset (regression), (3) ADHD diagnosis based on a Child Behavior Checklist (CBCL) T-score exceeding 65 in the ABCD dataset (classification), and (4) autism spectrum disorder (ASD) diagnosis in the ABIDE dataset (classification).

For each downstream task, participants with missing or invalid assessment scores were excluded from the analysis (listwise exclusion). Model performance was evaluated using the Area Under the Receiver Operating Characteristic curve (AUROC) for all classification tasks and the mean absolute error (MAE) for all regression tasks.

#### MBBN demonstrates superior performance across diverse neuroimaging tasks

**Fig. 1:**
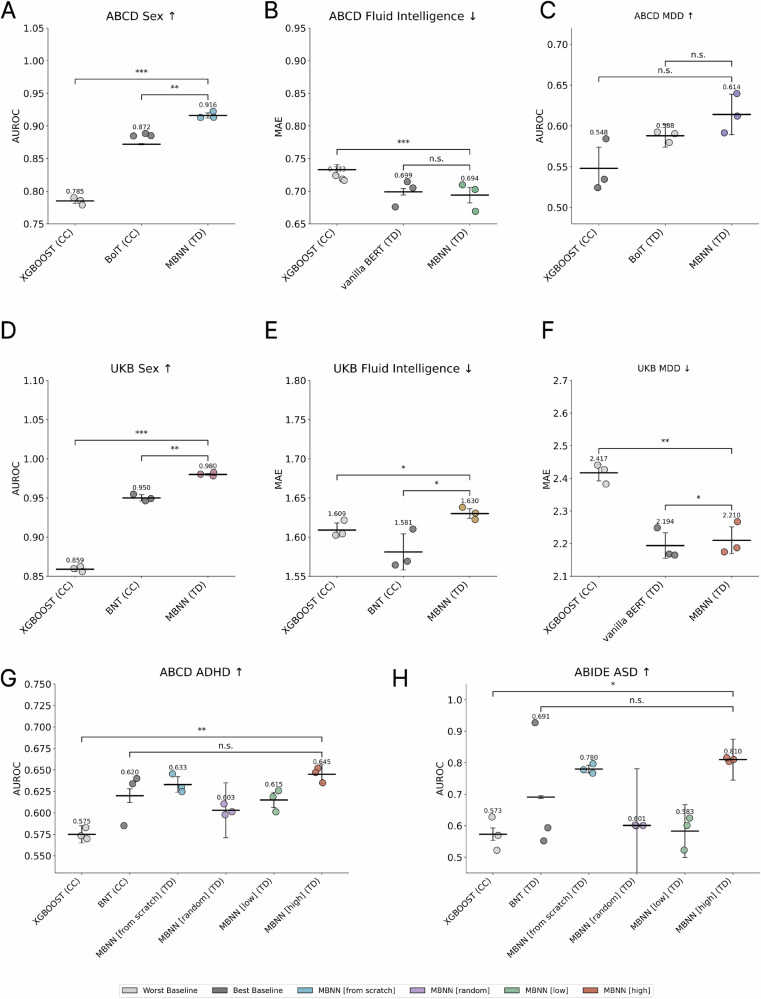
Comparative analysis of fMRI prediction models. Across datasets and tasks, MBBN demonstrates superior predictive performance compared with baseline models, including state-of-the-art approaches such as the Brain Network Transformer^5^ and a vanilla BERT model without multifractal decomposition. Each dot represents model performance from a single random seed (*n* = 3). Horizontal bars and error bars indicate the mean and standard error of the mean (SEM), respectively. **A** Sex prediction in the ABCD dataset (*n* = 8833). **B** Fluid intelligence prediction in the ABCD dataset (*n* = 5162). **C** MDD  prediction in the ABCD dataset (*n* = 3122). **D** Sex prediction in the UK Biobank dataset (*n* = 40,699). **E** Fluid intelligence prediction in the UK Biobank dataset (*n* = 21,464). **F** MDD  prediction in the UK Biobank dataset (*n* = 27,647). **G** ADHD classification in the ABCD dataset (*n* = 2938). **H** ASD classification in the ABIDE dataset (*n* = 141). Models labeled [CC] use Pearson correlation-based functional connectivity, whereas [TD] indicates models that incorporate temporal dynamics. The *x* axis represents model architectures and the *y* axis indicates prediction performance, measured using AUROC for classification tasks and MAE for regression tasks. Experiments in **A**–**F** correspond to models trained from scratch, whereas **G**, **H** show fine-tuning results. *** denotes *p* < 0.001, ** denotes *p* < 0.01, * denotes *p* < 0.05, and n.s. denotes not significant.

To evaluate its performance, MBBN was trained from scratch on a diverse set of downstream tasks spanning biological, cognitive, and clinical domains, utilizing both the HCP-MMP1 and Schaefer atlases with the UKB and ABCD datasets. While MBBN demonstrated unparalleled performance in classification tasks, its advantage in regression tasks (e.g., fluid intelligence and MDD score prediction) was less pronounced, suggesting that the model’s architecture is particularly optimized for distinguishing categorical phenotypes rather than predicting continuous scores. The primary results presented in Fig. 1A–F and Table 1 focus on experiments conducted with the HCP-MMP1 atlas; corresponding analyses using the Schaefer atlas are detailed in the Supplementary Materials 5.

**Table 1:** Performance gain of MBBN between the best and worst baseline models

Task	MBBN	vs best baseline (%)	vs worst baseline (%)
ABCD sex	0.916 (AUROC)	+2.346 (**)	+19.271 (***)
ABCD fluid intelligence	0.717 (MAE)	+0.693	+9.584 (***)
ABCD MDD	0.556 (AUROC)	+1.460	+7.961
UKB sex	0.980 (AUROC)	+3.158 (*)	+14.086 (***)
UKB fluid intelligence	1.630 (MAE)	−3.099 (*)	−1.305 (*)
UKB MDD	2.21 (MAE)	−1.231 (*)	+0.924 (**)
ABCD ADHD	0.645 (AUROC)	+4.032	+12.174 (**)
ABIDE ASD	0.810 (AUROC)	+16.212	+41.361 (*)

We illustrated fine-tuned MBBN for comparison of ABCD ADHD classification and ABIDE ASD classification. Statistical significance against baseline models was determined by a paired *t* test (*p < 0.05, **p < 0.01, ***p < 0.001). Statistical significance shown only where applicable.

For biological phenotype prediction, MBBN achieved substantial improvements in sex classification with gains up to 14.09% (UKB) and 19.27% (ABCD) using HCP-MMP1 atlas. Cognitive assessment showed marked improvements, with fluid intelligence prediction demonstrating up to 9.58% enhancement on ABCD. Also, clinical prediction tasks revealed MBBN’s therapeutic potential, achieving 0.92% improvement in MDD prediction (UKB) and exceptional performance in psychiatric disorders (detailed in Supplementary Materials 5).

#### Confound sensitivity and matched re-estimation

Using ABCD, we tested associations between knee frequencies and major confounds. Sex was associated with *f*_1_ only (not *f*_2_), suggesting a potential sex-linked physiological contribution at lower frequencies, whereas site effects were present for both knees (expected under 22-site acquisition heterogeneity). By contrast, age and head motion (mean FD) were not significantly related to either knee. To ensure that disorder effects were not driven by these factors, we constructed a propensity score–matched subsample jointly balancing sex, age, motion, and site; covariates were well balanced (standardized mean differences  < 0.10). After propensity score matching, AUROC increased from 0.633 to 0.651; however, the difference did not reach statistical significance under our paired comparison (*p* = 0.3742). Full association tables and balance diagnostics are provided in the Supplementary Materials 5.

#### Communicability-based pretraining enables superior clinical prediction performance

The exceptional performance of MBBN, particularly in clinical prediction tasks, stems from our novel communicability-based pretraining strategy that masks nodes with high communicability within the brain network. This strategy was predicated on the hypothesis that selectively reconstructing these highly influential nodes during pretraining would better represent the complex communication patterns within the brain. To validate this hypothesis and evaluate the downstream impact of this pretraining strategy, MBBN was subsequently fine-tuned on two clinically relevant classification tasks: ADHD classification using the ABCD dataset and ASD classification using the ABIDE dataset. These datasets and tasks were chosen for their established clinical significance and high prevalence rates.

Fine-tuning experiments (Fig. 1G, H) revealed a clear advantage for the MBBN-high model, which employed a pretraining strategy that selectively masked nodes with high communicability. This approach resulted in significant performance improvements compared to baselines: a 7.86% gain (*p* < 0.005) in ADHD classification AUROC and a 41.36% (*p* < 0.05) gain in ASD classification AUROC. In contrast, models using random node masking (MBBN-random) or masking nodes with low communicability (MBBN-low) showed inferior performance, even compared to the MBBN model trained from scratch (MBBN-from scratch). These findings underscore the critical role of communicability-based pretraining in effectively leveraging the underlying network structure of the data to enhance predictive performance. Detailed results and statistical details are available in Supplementary Materials 5. These results demonstrate that communicability-based loss better captures a complex information flow of the brain than conventional masking loss, and MBBN’s frequency-resolved approach captures clinically relevant neural signatures that are missed by conventional broadband analysis methods.

#### Frequency-specific connectivity patterns reveal distinct psychiatric biomarkers

**Fig. 2:**
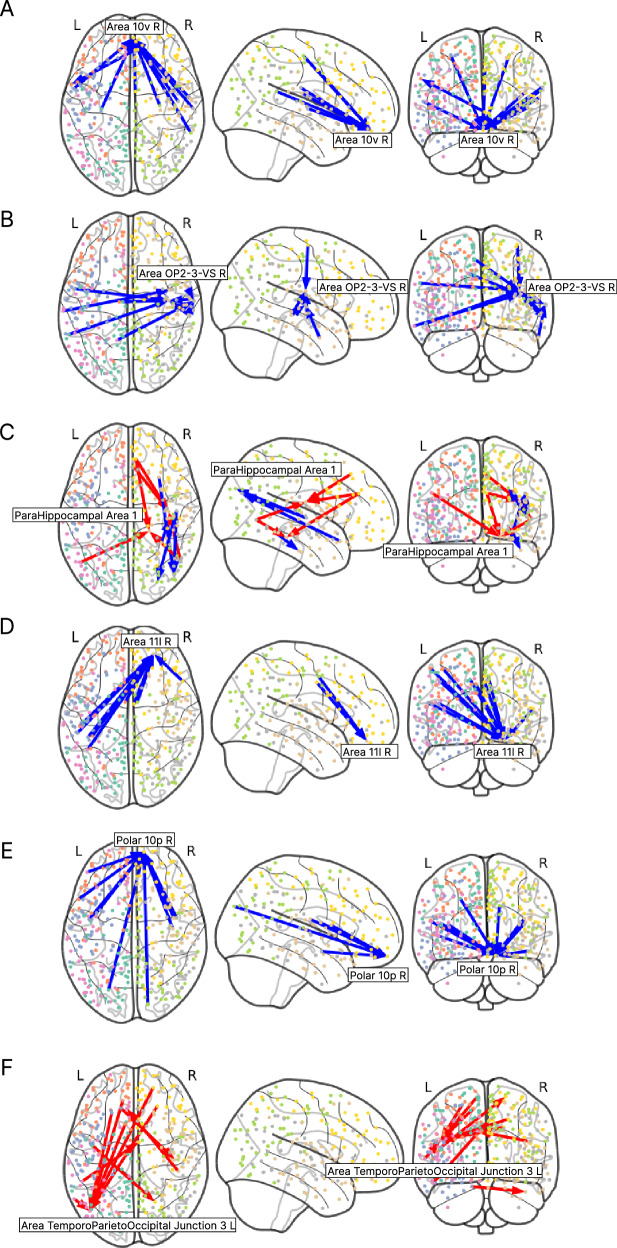
Frequency-specific connectivity disruptions in ADHD and ASD. Statistically significant connections (FDR-corrected *p* < 0.05, top 10 effect sizes) distinguishing individuals with ADHD (**A**–**C**) and ASD (**D**–**F**) from healthy controls are shown. **A**–**C** Highlight ADHD-related connectivity patterns centered on the right ventral Brodmann Area 10 (**A**), right parietal operculum (**B**), and parahippocampal gyrus (**C**). **D**–**F** illustrate ASD-related connectivity patterns centered on the right lateral Brodmann Area 11 (**D**), right polar Brodmann Area 10 (**E**), and the left temporo-parieto-occipital junction (**F**). Red indicates increased connectivity in the disorder group relative to controls, whereas blue indicates decreased connectivity. Additional statistical details are provided in the Supplementary Information.

To identify the neurobiological features that our model learned to distinguish between psychiatric and control groups, we applied a Grad-CAM-based attribution analysis. This method allowed us to extract the specific, frequency-dependent attention patterns that were most influential in the model’s classification decisions. By examining these discriminative patterns, we uncovered distinct neural signatures for ADHD and ASD that were not apparent in conventional broadband analyses, thereby validating that the model had learned clinically relevant information (See Section 5 for details). Figure 2 focuses specifically on ADHD and ASD because these are the representative psychiatric disorders for which our model achieved the highest classification performance, thereby providing a clear and effective demonstration of the model’s interpretability.

In the ABCD test dataset, we identified significant group differences between individuals with ADHD and healthy controls (HC) in the model’s learned attention patterns across frequency bands (Fig. 2A–C). The analysis revealed that the most extensive alterations occurred in the high-frequency band, accounting for 8.43% of all significant inter-regional attention differences (*p* < 0.05). These patterns showed moderate effect sizes (75th percentile Cohen’s *d* = 0.332). In contrast, the low-frequency and ultralow-frequency bands exhibited more localized but still significant alterations, accounting for 3.46% (Cohen’s *d* 75th percentile = 0.220) and 3.56% (Cohen’s *d* 75th percentile = 0.117) of the total discriminative patterns, respectively.

Anatomically, the high-frequency patterns that the model learned to distinguish ADHD centered on Area 10v (ventral part of Brodmann Area 10), implicating networks crucial for executive control. The discriminative low-frequency patterns consistently involved Area OP2-3-VS (opercular parts of Areas 2 and 3 and adjacent Ventral Somatosensory/Sylvian region) and the Auditory 4 Complex, suggesting that the model leveraged features related to sensorimotor-auditory integration. Finally, the subtle ultralow-frequency patterns in the Medial Belt Complex and ParaHippocampal regions point to alterations in memory and emotional processing circuits that contributed to the model’s classification.

**Fig. 3:**
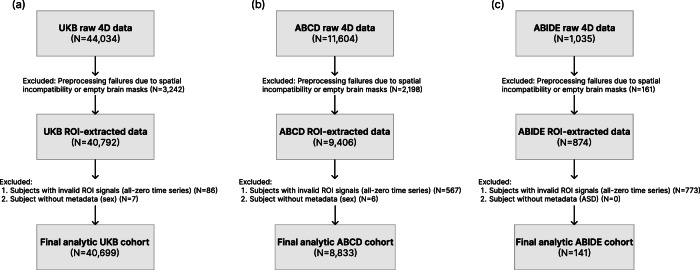
Participant selection and quality control across datasets. Flowcharts summarize the stepwise subject inclusion and exclusion procedures for **a** UK Biobank (UKB), **b** Adolescent Brain Cognitive Development (ABCD), and **c** Autism Brain Imaging Data Exchange (ABIDE). Across datasets, raw 4D resting-state fMRI data were first excluded if preprocessing failed due to spatial incompatibility or empty brain masks. Atlas-based ROI time series were then extracted, after which subjects were further excluded if invalid ROI signals were detected (defined as all-zero time series in one or more ROIs) or if required metadata were missing. Final analytic cohort sizes after all quality control steps are reported for each dataset.

In the ABIDE dataset, the model learned a distinct set of discriminative features for ASD classification, with a contrasting frequency profile (Fig.3 2D–F). T-test comparisons revealed that the most extensive alterations in learned attention weights were in the ultralow-frequency band, representing 4.54% of all significant patterns with large effect sizes (75th percentile Cohen’s *d* = 0.717). Low-frequency alterations were similarly prominent, accounting for 4.04% of significant patterns with substantial effect sizes (Cohen’s *d* 75th percentile = 0.714). Notably, the model found high-frequency patterns to be the least discriminative, comprising only 1.81% of significant features, though these still showed moderate effect sizes (Cohen’s *d* 75th percentile = 0.575).

The few high-frequency patterns the model used for classification were localized to Area 11l (lateral part of Brodmann Area 11), consistent with its role in social-cognitive functions. The prominent low-frequency discriminative features affected Area 10pp (frontopolar cortex), implicating higher-order executive functions. Most significantly, the extensive ultralow-frequency attention patterns involved Area TPOJ3 (temporo-parieto-occipital junction) and multiple frontal regions. This indicates that the model’s classification heavily relied on features related to fundamental disruptions in social cognition and language networks—core domains of ASD pathophysiology.

In both the ABCD and ABIDE datasets, analysis of false positive and false negative subjects (based on predicted values) showed no statistically significant connections in the self-attention-based connectivity analysis. These frequency-resolved patterns reveal disorder-specific neural signatures: ADHD primarily affects high-frequency sensorimotor networks with compensatory ultralow-frequency changes, while ASD shows pervasive low and ultralow-frequency disruptions in social-cognitive circuits. Importantly, false positive and false negative cases showed no significant connectivity patterns, confirming the specificity of these frequency-dependent biomarkers for accurate psychiatric classification.

## Methods

### Preprocessing

The ABCD, UKB, and ABIDE datasets underwent dataset-specific preprocessing pipelines to honor the official protocols and best practices established by each respective consortium. In ABCD, a conservative bandpass filter (0.009–0.08 Hz) was applied following consortium protocols for adolescent populations^20^. UKB employed a broader filter (0.008–0.1 Hz) optimized for adult populations to minimize heart rate variability effects^21^. ABIDE used finite impulse response (FIR) filtering (0.01–0.1 Hz) specifically designed for multi-site ASD data integration^22^.

For the present study, additional preprocessing was performed on the resting-state BOLD data. The following preprocessing procedures were applied to each participant’s resting-state BOLD data: First, skull-stripping, slice-timing correction, susceptibility distortion correction, and spatial normalization to MNI space were conducted using fMRIPrep^23^. Subsequently, head motion artifacts and signals from white matter were removed using component-based noise correction (CompCor)^24^. The mean time series signals from predefined regions of interest (ROIs) were extracted using the Nilearn package, with HCP-MMP1 asymmetric^25^ and Schaefer 400^26^ atlases employed for ROI definitions. Participants whose data contained voxels with NaN values, typically arising from the regression of unreliable white-matter signals, were excluded from the analysis. Additionally, to minimize spurious signal fluctuations due to scanner instability, the first 20 volumes of each time series were discarded.

For deep neural network training, uniform sequence lengths across all participants were required. To standardize the sequence length, it was set to the smallest multiple of eight that was greater than or equal to the shortest sequence length in the dataset, optimizing computational efficiency for GPU-based matrix operations. The final sequence lengths for the datasets were as follows: UK Biobank (UKB) 464, Adolescent Brain Cognitive Development (ABCD) 348, and Autism Brain Imaging Data Exchange (ABIDE) 280.

### Dataset description and subject selection criteria

This study utilized three large-scale, publicly available datasets: the UK Biobank (UKB), the Adolescent Brain Cognitive Development (ABCD) Study, and the Autism Brain Imaging Data Exchange (ABIDE). The final sample size for each cohort was determined following a rigorous quality control and subject selection procedure.

Figure 3 summarizes the stepwise subject selection and attrition procedure for each dataset, including imaging availability, quality control, preprocessing success, and phenotype completeness. All sample sizes reported in the text, tables, and supplementary materials were cross-checked for numerical consistency.

UK Biobank (UKB): The UKB dataset is a prospective cohort study of ∼500,000 adults. For our study, we included participants who had available resting-state fMRI data. Subjects were excluded if preprocessing failed due to spatial incompatibility or empty brain masks, or if invalid ROI signals were detected after atlas-based extraction (e.g., all-zero time series). Participants without the required metadata were also excluded. These procedures resulted in a final analytic sample of 40,699 individuals.

Adolescent Brain Cognitive Development (ABCD) Study: We used the baseline, year-one data from the ABCD Study, a longitudinal study of brain development in U.S. children. The initial release contained imaging data from 11,604 participants. All subjects included in the present study passed the standardized MRI quality control procedures provided by the ABCD consortium. Additional exclusions were applied if preprocessing failed due to spatial incompatibility or empty brain masks, or if invalid ROI signals were identified after atlas-based extraction (e.g., all-zero time series). Participants without relevant behavioral or diagnostic data required for downstream tasks were also excluded. These criteria yielded a final analytic sample of 8833 participants.

Autism Brain Imaging Data Exchange (ABIDE): We used data from the ABIDE-I cohort, which aggregates resting-state fMRI data from 17 international acquisition sites. All sites were included to maximize sample heterogeneity. Subjects were excluded if preprocessing failed due to spatial incompatibility or empty brain masks, if atlas-based ROI time series extraction resulted in invalid signals (e.g., all-zero time series), or if diagnostic metadata were incomplete. Quality control information from the Preprocessed Connectomes Project was used as an initial reference. After applying these criteria uniformly across subjects, the final analytic sample consisted of 141 participants for the ASD classification task.

Demographic and diagnosis distribution details and neuroimaging parameter information for the three datasets (UKB, ABCD, ABIDE) are summarized in Tables 2, 3. The UK Biobank (UKB) dataset, which includes 40,699 participants, was used for pretraining due to its large sample size and broad demographic representation. This extensive dataset enabled the model to capture generalizable brain dynamics patterns. The Adolescent Brain Cognitive Development (ABCD) dataset, consisting of 4527 participants (including individuals with ADHD and healthy controls), and the Autism Brain Imaging Data Exchange (ABIDE) dataset, which includes 141 participants (comprising individuals with ASD and healthy controls), were employed for fine-tuning to capture disorder-specific patterns.

**Table 2:** Demographic and clinical characteristics of the study cohorts

Feature	UKB	ABCD	ABIDE
Overall analytic cohort			
Total *N*	40,699	8833	141
Gender (M/F)	21,556/19,143	4635/ 4198	114/27
Age (years)	54.9 ± 7.5	–	14.3 ± 3.6
Age (months)	–	119.2 ± 7.5	–
Task-specific subsets			
MDD (disorder/controls)	–	2652/461 (*N* = 3113)	–
ADHD (disorder/controls)	–	2439/1850 (*N* = 4289)	–
ASD (disorder/controls)	–	–	77/64 (*N* = 141)
MDD severity (PHQ-9 score)	11.5 ± 3.5	–	–

Overall cohort characteristics are reported for the final analytic samples of each dataset. Disorder/control counts are reported for task-specific subsets used in downstream analyses. Values are presented as mean ± standard deviation unless otherwise indicated.

**Table 3:** Key acquisition parameters for each dataset

Parameter	UKB	ABCD	ABIDE
Repetition time (TR, s)	0.735	0.8	Various (1.5–3.0)
Echo time (TE, ms)	39	30	Various
Voxel size (mm^3^)	2.4 × 2.4 × 2.4	2.4 × 2.4 × 2.4	Various

Key MRI acquisition parameters for the UK Biobank (UKB), Adolescent Brain Cognitive Development (ABCD), and Autism Brain Imaging Data Exchange (ABIDE) datasets.

ADHD diagnosis was based on CBCL attention and ADHD scores exceeding a T-score threshold of 65, while healthy controls were defined as individuals with no diagnosed mental health disorders^27^. This classification ensured a distinct separation between clinical and non-clinical groups, facilitating model training and evaluation.

Our selection of downstream prediction tasks was guided by the prioritization of well-established phenotypes that ensure large sample sizes, minimal missing data, and high clinical relevance for classification frameworks.

For the ABCD dataset, we focused on a binary classification of MDD (disorder vs. controls) rather than regression on continuous symptom scores. This choice was motivated by the goal of testing the model’s utility in a categorical diagnostic context, which aligns with common clinical applications and provides a clear, robust target for classification models.

For the ABIDE dataset, we similarly prioritized the primary diagnostic label (ASD vs. typically developing controls) over continuous behavioral metrics such as the Social Responsiveness Scale (SRS) or Autism Diagnostic Observation Schedule. This decision was driven by the need for maximum data standardization and reliability. The primary diagnosis is the most consistently harmonized variable across the many different data collection sites within the ABIDE consortium, whereas behavioral scores can be subject to significant inter-site variability in administration and scoring.

### Experimental settings

To address the imbalance in clinical variables within the dataset, stratified sampling was employed to ensure balanced distributions of target variables (ADHD, MDD, ASD) across the training, validation, and test sets. A similar approach was used for the biological phenotype (sex), which showed minimal imbalance. To enhance model robustness and reduce overfitting, training was conducted using three random seeds, and the average performance across these runs was reported.

Gradient clipping with a norm of one and gradient accumulation were applied to stabilize training and improve predictive accuracy. To accelerate the training process, AMP was utilized. The BERT model architecture comprised eight hidden layers and eight or 12 attention heads, depending on the number of ROIs in the atlas used. The number of attention heads was chosen based on the constraint that it should be a divisor of the total number of ROIs. Hyperparameter optimization included testing learning rates ([1e-5, 1e-2]), weight decay ([1e-3, 1e-2]), learning rate policy (step, SGDR), and optimizers (Adam, AdamW, RMSprop).

For computational efficiency, pretraining was performed on a single A100 GPU, while training from scratch and fine-tuning were conducted using a single NVIDIA RTX 3090 GPU. The number of epochs was set to 100 for training from scratch. Efficient pretraining was achieved by determining the optimal number of epochs using weightwatcher^28^, which identifies well-trained layers based on statistical learning theory. Optimal pretraining epochs were set to 1000 for the HCP-MMP1 atlas and 400 for the Schaefer 400 atlas. Additional methodological details are provided in Supplementary Materials 5.

### Frequency-resolved decomposition based on scale-free principles

Brain activity exhibits multifractal properties with distinct scaling behaviors across frequency ranges, reflecting the hierarchical organization of neural networks from local circuits to global integration pathways. Conventional broadband connectivity analysis obscures these frequency-specific dynamics, potentially missing critical neurophysiological signatures of psychiatric disorders. To capture these frequency-dependent neural processes, we developed a systematic two-step fitting procedure to identify physiologically meaningful frequency boundaries (knee frequencies) that demarcate distinct scaling regimes in the power spectral density of resting-state fMRI signals. Following frequency decomposition using the identified knee frequencies, we confirmed that each of the three segments exhibited distinct power-law scaling behavior, as evidenced by significantly different *β* values across frequency bands (*p* < 0.001).

#### Step 1: Ultralow-frequency boundary identification using Lorentzian fitting

We first applied the Lorentzian equation (equation (2)) to the entire power spectral density to identify *f*_1_, the primary knee frequency that separates ultralow frequency activity from higher-frequency neural processes:

2 $$Power( \, f)=\frac{A\cdot {f}_{1}^{2}}{{f}^{2}+{f}_{1}^{2}}$$

where *A* represents the amplitude parameter and *f*_1_ denotes the characteristic frequency at which the power spectrum transitions from flat (low-frequency plateau) to steep decay (high-frequency roll-off). This Lorentzian model effectively captures the fundamental 1/f characteristics of neural signals while identifying the critical transition point. The fitting was performed using SciPy’s curve_fit function, which is well-suited for this two-parameter optimization problem. Physiologically, *f*_1_ corresponds to the boundary between slow cortical potentials and faster network dynamics, typically associated with default mode network fluctuations and global integration processes.

#### Step 2: Low-high frequency boundary identification using spline multifractal modeling

For frequencies above *f*_1_, we applied a sophisticated spline multifractal equation to identify *f*_2_, the second knee frequency that distinguishes low-frequency network connectivity from high-frequency local processing:

To ensure smooth transitions between different scaling regimes and prevent fitting artifacts, we implemented a cubic spline weight function:

3 $$w(f)=\left\{\begin{array}{ll}0 \hfill & \,{{\rm{if}}}\,\log (f) < \log ({f}_{2})-s\\ 1 \hfill & \,{{\rm{if}}}\,\log (f) > \log ({f}_{2})+s\\ \frac{1}{2}(1-\cos (\frac{\pi (\log (f)-\log ({f}_{2})+s)}{2s})) & \,{{\rm{otherwise}}} \hfill \end{array}\right.$$

This weight function *w*(*f*) provides a smooth, differentiable transition between two distinct power-law regimes. When *f* ≪ *f*_2_, *w*(*f*) ≈ 0, ensuring the function follows low-frequency scaling behavior characterized by *β*_low_. Conversely, when *f* ≫ *f*_2_, *w*(*f*) ≈ 1, shifting to high-frequency scaling defined by *β*_high_. The smoothness parameter *s* controls the sharpness of this transition, preventing abrupt discontinuities that could introduce fitting artifacts.

The complete spline multifractal function then becomes:

4 $$Power( \, f)=A\cdot {f}^{{\beta }_{low}(1-w( \, f))+{\beta }_{high}w(f)}$$

where:

- *A* is the amplitude scaling factor
- *β*_low_ is the low-frequency scaling exponent (typically steeper, reflecting long-range correlations)
- *β*_high_ is the high-frequency scaling exponent (typically flatter, reflecting local processing)
- *f*_2_ is the second knee frequency (transition point between network and local dynamics)
- *s* is the smoothness parameter controlling transition width

This five-parameter model was fitted using the iminuit package, specifically designed for high-dimensional parameter estimation with robust convergence properties. Physiologically, *f*_2_ represents the transition from large-scale network connectivity to localized neural processing, typically corresponding to the boundary between task-negative and task-positive network activity.

#### Frequency band segmentation and filtering implementation

Based on the identified knee frequencies *f*_1_ and *f*_2_, we segmented each participant’s time series data into three physiologically distinct frequency bands:

1. Ultra-low frequency band ( < *f*_1_): Captures slow cortical potentials, default mode network activity, and global integration processes
2. Low frequency band (*f*_1_ to *f*_2_): Represents intermediate-scale network connectivity and inter-regional communication
3. High frequency band ( > *f*_2_): Reflects local circuit dynamics, sensorimotor processing, and rapid cognitive operations

The segmentation was implemented using FIR filters from the nitime package for ultra-low and low-frequency bands, and Boxcar filters for high-frequency extraction. The choice of filter types was optimized through systematic hyperparameter tuning on validation sets, evaluating their impact on downstream classification performance. FIR filters were preferred for lower frequencies due to their superior phase preservation properties, while Boxcar filters provided computational efficiency for high-frequency components without compromising signal quality.

#### Validation of frequency-specific scaling properties

Table 4 reports the dataset-wise knee frequencies (*f*_1_, *f*_2_; mean ± s.d.), together with the acquisition repetition time (TR) and the corresponding Nyquist limit (${{\rm{Nyquist}}}=1/(2\cdot {{\rm{TR}}})$). As expected, absolute knee values differ across datasets because the analyzable frequency range is physically bounded by TR: ABCD (TR  = 0.735 s; Nyquist = 0.6803 Hz) and UKB (TR = 0.8 s; Nyquist = 0.625 Hz) exhibit higher *f*_1_/*f*_2_ than ABIDE, which spans longer TRs (1.5–3.0 s; Nyquist = 0.1667–0.3333 Hz). Importantly, when expressed relative to the Nyquist limit, the knees occupy comparable portions of the spectrum across datasets—*f*_1_ ≈ 5–10% and *f*_2_ ≈ 9–18% of Nyquist. This pattern indicates that our data-driven procedure adapts to each dataset’s spectral support while preserving a consistent relative band structure, in line with the TR/Nyquist constraints of fMRI acquisitions.

In addition to the TR/Nyquist effects summarized in Table 4, we verified that the learned knees are robust along three axes: population, measurement time, and boundary shifts. First, bootstrap resampling across subgroups revealed no significant differences in *f*_1_ or *f*_2_ (*p* = 0.6541 and *p* = 0.8956). Second, an HCP test–retest analysis (run–1 vs. run–2) showed no significant shifts (*p* = 1.000 for *f*_1_; *p* = 0.9428 for *f*_2_). Third, perturbing each cutoff by  ± 5–10% yielded a broad performance plateau centered at the learned boundaries (peak AUROC  = 0.916), indicating insensitivity to small boundary changes. Full procedures and results are provided in Supplementary Materials 5.

**Table 4:** Different *f*_1_, *f*_2_, TR and Nyquist frequency among datasets

Dataset	*f*_1_ (Hz)	*f*_2_ (Hz)	TR (s)	Nyquist (Hz)
ABCD	0.0456 ± 0.0106	0.0784 ± 0.0151	0.735	0.6803
UKB	0.0624 ± 0.0113	0.0961 ± 0.0157	0.8	0.625
ABIDE	0.0182 ± 0.0121	0.0299 ± 0.0256	1.5–3.0	0.1667–0.3333

Dataset-specific frequency boundary estimates and acquisition parameters. The knee frequencies *f*_1_ and *f*_2_ define the boundaries between ultralow, low, and high frequency ranges. Values for *f*_1_ and *f*_2_ are reported as mean ± s.d.

Before examining frequency-specific scaling properties, we first assessed the goodness-of-fit of our two-step fitting procedure. Across all three datasets, both the Lorentzian and spline multifractal equations achieved high R² values (Table 5), confirming that the identified knee frequencies (f1, f2) reliably captured the underlying power-law structure of the fMRI spectrum. To further validate that our frequency decomposition captured distinct neurophysiological processes, we computed scaling exponents (*β* values) for each frequency band using power-law fitting (Table 6):

**Table 5:** Goodness of fit (*R*^2^) statistics for Lorentzian and spline multifractal equations across datasets

Dataset	Lorentzian equation	Spline multifractal equation
	Mean *R*^2^	Mean *R*^2^
ABCD	0.7423 ± 0.0765	0.6822 ± 0.2375
UKB	0.6605 ± 0.0786	0.8292 ± 0.0854
ABIDE	0.7435 ± 0.1018	0.8309 ± 0.1019

Values are presented as mean  ± s.d. *R*^2^ indicates the proportion of variance explained by each model.

**Table 6:** Different *β* value among frequency ranges and datasets

Dataset	*β*_ultralow_	*β*_low_	*β*_high_
ABCD	0.2442 ± 0.2991	1.7845 ± 0.3836	1.1595 ± 0.4260
UKB	0.0209 ± 0.2185	2.8959 ± 0.4562	1.1232 ± 0.4107
ABIDE	0.5805 ± 0.3925	2.5939 ± 0.3923	0.9563 ± 0.2228

Dataset-specific power-law scaling exponents (*β*) across ultralow, low, and high frequency ranges. Values are reported as mean  ± s.d.

Statistical analysis revealed significant differences in *β* values across all frequency bands (*p* < 0.001), confirming that each band exhibits unique scale-free dynamics. Ultralow frequencies showed the flattest scaling (smallest *β*), consistent with long-range temporal correlations. High frequencies exhibited intermediate scaling, reflecting network-level integration. Low frequencies displayed the steepest scaling (largest *β*), characteristic of local, rapidly decorrelated processes. These findings support the hypothesis that frequency-specific decomposition captures fundamentally different aspects of brain organization, from global integration to local specialization.

#### Validation of frequency threshold stability

To rigorously evaluate the stability and reproducibility of our data-driven frequency thresholds (*f*_1_ and *f*_2_), we performed a non-parametric bootstrapping procedure with 1000 iterations. In each iteration, we randomly sub-sampled 80% of the subjects from the original cohort and re-calculated the subject-specific *f*_1_ and *f*_2_ distributions. We then assessed the stability of both the central tendency and the overall distributional shape. The central tendencies of the thresholds demonstrated exceptional stability; the distribution of differences between the sampled means and the original population means was tightly centered at zero with negligible deviation for both *f*_1_ and *f*_2_. Furthermore, to assess the stability of the entire distributional shape, we performed a two-sample Kolmogorov-Smirnov test for each iteration. The resulting *p* values were overwhelmingly non-significant (*p* > 0.05 in over 95% of iterations for both thresholds), indicating that the distributions derived from the sub-samples were not statistically different from the original population’s distribution. Taken together, these results confirm that our method for defining frequency bands is robust, reproducible, and generalizable across subjects, thus validating its use for the primary analyses in this study.

#### Comparison with other frequency-dividing methods

Our frequency decomposition is based on a physiologically grounded, data-driven characterization of the scale-free fMRI spectrum: we fit Lorentzian components and locate spectral knees with a spline procedure to obtain dataset-specific cutoffs (*f*_1_, *f*_2_). We compared this approach with two widely used alternatives.

First, a fixed-band scheme (Slow-5: 0.01–0.027 Hz; Slow-4: 0.027–0.073 Hz^29^ yielded a modest but consistent drop in performance for the same tasks (AUROC  = 0.911 vs. 0.916 with our knees; Supplementary Materials 5).

Second, we implemented an adaptive alternative approach that (i) computes the Welch power spectral density, (ii) clusters frequencies into three groups via power-weighted *k*-means (*k* = 5), (iii) uses the midpoints between adjacent clusters as two cutoffs, and (iv) performs wavelet-based decomposition using those data-driven boundaries^30^. This adaptive variant achieved AUROC  = 0.905, broadly consistent yet modestly below our knee-based method (Supplementary Materials 5).

#### Confound analyses and propensity score matching

Associations between knee frequencies (*f*_1_, *f*_2_) and sex, age, head motion (mean FD), and site (22 levels) were assessed using regression/AN(C)OVA with site modeled as a factor; multiplicity-adjusted *p* values are reported in Supplementary Materials 5. For sensitivity, we performed 1:1 nearest-neighbor propensity score matching (logistic model; covariates: sex, age, mean FD, site; caliper = 0.2 SD of the logit; no replacement) to create a confound-balanced subsample. Balance was confirmed by standardized mean differences  < 0.10 for all matched covariates. Classification AUROC was then compared before and after matching using a paired *t* test.

### Model design

#### Model architecture

**Fig. 4:**
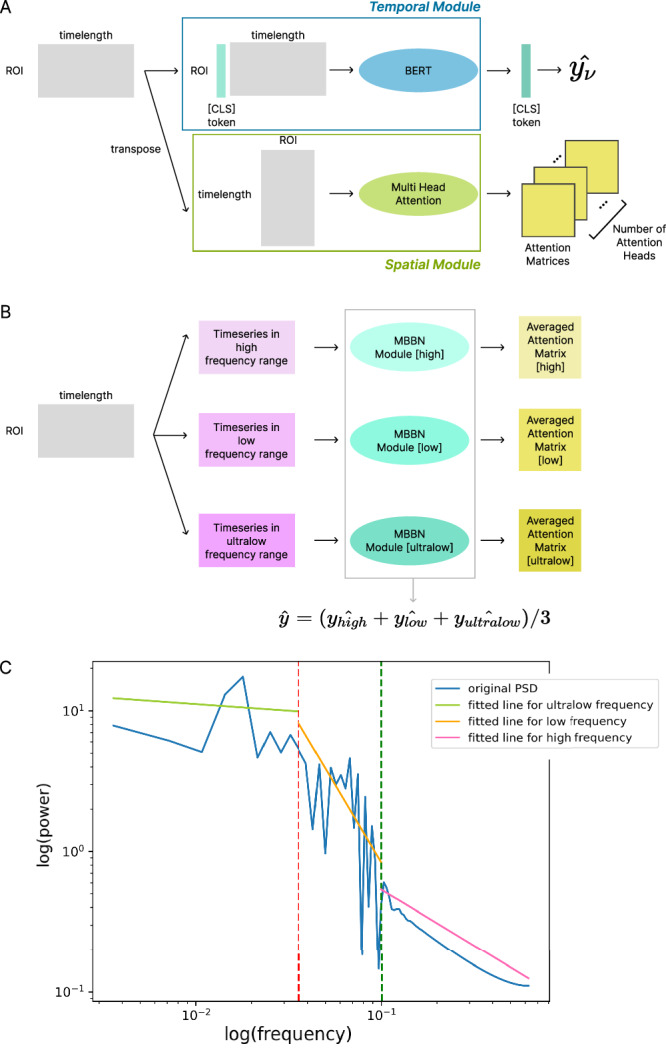
Participant selection and quality control across datasets. **a** The MBBN module processes each time series signal after frequency decomposition. In the temporal module (denoted as bert), the model updates the [cls] (classification) token, which is passed through a classifier to generate a prediction ${\widehat{y}}_{\nu }$, where *ν* denotes the frequency range. The spatial module employs a multi-head attention mechanism to compute attention matrices representing interactions between regions of interest (rois). **b** MBBN modules are applied to time series signals across three frequency ranges (ultra-low, low, and high frequency). Parameter sharing is implemented in the temporal module, while spatial modules have independent parameters for each frequency range. The final output is computed as the average prediction across frequency ranges together with frequency-specific attention matrices. **c** Frequency decomposition based on scale-free principles applied to representative UK biobank data. individual-specific knee frequencies (*f*_1_, *f*_2_) separate three biologically distinct bands: high (pink), low (yellow), and ultralow (yellow-green), each exhibiting characteristic power-law scaling. The dotted red and green lines indicate the frequency boundaries between ultra-low and low (*f*_1_) and between low and high (*f*_2_) frequencies.

MBBN integrates temporal and spatial processing through a dual-module architecture that consists of the temporal module and the spatial module. These modules work synergistically to encode both the temporal and spatial characteristics of frequency-divided time series data, as shown in Fig. 4A. To ensure an effective balance between generalization and specificity, a parameter-sharing strategy is employed, as outlined below.

The temporal module (depicted as a blue oval in Fig. 4A) is designed to capture the temporal dynamics of the data. For each frequency-divided time series (ultra-low, low, and high), the data are processed independently using a shared BERT encoder, reflecting the temporal similarities across frequency bands. The BERT encoder outputs a sequence of hidden states for each time series, with the final hidden state corresponding to the [CLS] token passed to a classifier. This process produces logits ($\widehat{{y}_{ultralow}}$, $\widehat{{y}_{low}}$, and $\widehat{{y}_{high}}$), which represent the model’s predictions for each frequency range. The hidden dimension size of the MBBN temporal module is set to match the number of ROIs. Due to the constraints imposed by the BERT backbone in MBBN, the number of attention heads must be a divisor of the hidden dimension size. Consequently, when using the Schaefer atlas, the number of attention heads is set to 8, whereas for the HCP MMP1 atlas, it is set to 12.

The spatial module (depicted as a yellow-green oval in Fig. 4A) focuses on the spatial connectivity among ROIs. Unlike the temporal module, the spatial module uses a frequency-specific approach to capture the distinct spatial patterns of each frequency band. Specifically, a multi-head attention mechanism is applied to the transposed time series data, generating a set of attention matrices for each frequency band. These attention matrices-reflecting the distinct focus of different heads-are averaged to produce a single attention matrix per band. These averaged matrices represent the spatial connectivity patterns, which are subsequently used to compute the spatial loss (equation (9)). In practice, the MBBN spatial module receives the transposed input of the temporal module, so the number of attention heads must divide the sequence length. For example, we set 12 heads for ABCD (sequence length is 348) and 8 heads for both UKB (464) and ABIDE (280).

Although the temporal and spatial modules do not directly exchange hidden states, they remain closely coupled through the composite loss function. The total loss combines cross-entropy (or task-specific) loss with the spatial loss, so backpropagation updates parameters across the entire network. As a result, while the BERT-based temporal module primarily absorbs gradient updates from the mask and task objectives, and the attention-based spatial module is mostly driven by spatial loss, their optimization processes are fundamentally interdependent. Changes in one module can indirectly shift the parameter landscape of the other, ensuring a shared optimization of temporal and spatial patterns.

This architectural design supports module specialization-BERT focuses on temporal dependencies, while the attention module models spatial relationships-without direct interference. Yet both modules ultimately contribute to the network’s overall optimization, facilitating a more integrated representation of brain dynamics. Additionally, by treating these modules as partly separable, the framework allows for independent evaluation and targeted enhancements in each module, preserving their complementary roles in the final model.

As shown in Fig. 4 B, the temporal and spatial modules operate on each frequency range, generating corresponding logits and attention matrices. To obtain the final prediction, the logits from the three frequency ranges are averaged and compared with the true label (*y*_true_) using a task-specific loss function (*Loss*_task_). In parallel, a spatial loss is computed to enforce distinct spatial connectivity patterns across frequency bands. The composite loss function enforces both task performance and frequency-specific spatial patterns:

5 $$Los{s}_{{\mathrm{total}}}=Los{s}_{{\mathrm{task}}}({y}_{{\mathrm{true}}},\frac{\widehat{{y}_{{\mathrm{ultralow}}}}+\widehat{{y}_{{\mathrm{low}}}}+\widehat{{y}_{{\mathrm{high}}}}}{3})+\lambda \cdot Los{s}_{{\mathrm{spatial}}}$$

where task-specific loss ensures predictive accuracy while spatial loss encourages distinct connectivity patterns across frequency bands.

In Equation (5), *Loss*_task_ represents the cross-entropy loss for classification tasks or L1 loss for regression tasks. The *Loss*_spatial_ term, as described in Equation (9), emphasizes the distinct spatial connectivity patterns inherent to each frequency band. *λ* was determined by hyperparameter tuning. The chosen *λ* value and the filter type were shown in Table 7.

**Table 7:** Optimal filter types and *λ* determined through hyperparameter tuning

Dataset	Filter type	Spatial factor *λ*
Schaefer atlas		
ABCD	Boxcar	10
UKB	Boxcar	1
ABIDE	Boxcar	100
HCPMMP1 atlas		
ABCD	FIR	10
UKB	Boxcar	1
ABIDE	Boxcar	100

Optimal filter types and spatial regularization factors (*λ*) selected through hyperparameter tuning for each dataset and brain atlas.

#### Parameter sharing

To enhance model performance, we adopted a multi-view learning approach, which is well-suited for frequency-divided time series data. We treat the ultra-low, low, and high frequency time series as three ‘views’ of the same underlying data, where different representations of the same underlying phenomenon are considered. Multi-view data enables the model to leverage shared information across views, allowing it to capture complementary aspects of the data.

For the temporal module, we utilized a parameter-sharing strategy. Given that temporal dynamics exhibited minimal differences across frequency bands (as indicated by similar autocorrelation structures), a single shared BERT encoder was used to process time series data from all frequency bands. This approach allows the model to exploit common temporal patterns across frequency bands, leading to a robust and generalized representation of temporal dynamics.

In contrast, the spatial module required a frequency-specific approach. Previous studies^31^ and our analysis have shown that spatial connectivity patterns differ significantly across frequency bands. Therefore, we implemented frequency-specific parameters for the spatial module. Self-attention-weighted connectivity was calculated independently for each frequency range, with each frequency band using its own set of parameters to capture the unique spatial relationships among ROIs. This design ensures that the model preserves the distinct connectivity characteristics of each frequency band while maintaining the interpretability of spatial attention.

This parameter-sharing strategy strikes a balance between generalization and specificity. Shared temporal parameters capture global patterns common across frequency bands, while frequency-specific spatial parameters emphasize the unique spatial relationships within each frequency band. By integrating this approach with task-specific and spatial loss functions (equation (5)), the model effectively captures the multi-level organization of frequency-divided time series data, facilitating the identification of relevant biomarkers across multiple scales.

### Temporal BERT module

#### Data encoding

To capture temporal dependencies across ROIs, the frequency-divided time series data were sequentially input into the BERT model. Each signal at a single time point was treated as a distinct token, with the token length corresponding to the total number of ROIs. This encoding strategy effectively represented both the temporal and spatial dynamics of the fMRI data, ensuring that these features were preserved for downstream analysis.

#### Using original form of BERT

FMRI BOLD signal data consists of long sequences, typically ranging from 280 to 464 tokens, necessitating a model that can handle such extended sequences. BERT, originally developed for natural language processing tasks, is particularly well-suited for this purpose due to its ability to capture complex contextual relationships within sequences^32^. This makes BERT an ideal model for modeling the intricate temporal dependencies inherent in fMRI data.

In contrast, models like GPT are optimized for text generation tasks and do not emphasize inter-sequence contextual relationships, which are critical for our analysis. Unlike GPT’s causal masking, which is tailored to text generation and unidirectional context BERT’s bidirectional attention allows it to capture long-range temporal dependencies from both past and future time points simultaneously, making it more suitable for modeling complex fMRI time series. Although some previous studies have used convolutional filters as a preprocessing step before entering signals into BERT^33^, we chose to bypass this step. Although convolutional preprocessing can be computationally efficient, it may sacrifice the preservation of fine-grained temporal dynamics within the fMRI data, which could impact model performance (see Supplementary Materials 5). By directly inputting the time series data into the original BERT structure, we aimed to retain the full complexity of the temporal dynamics, allowing the model to effectively capture both short-term and long-term dependencies.

To derive a single, task-relevant representation from the entire fMRI time series, we utilize the special [CLS] token, a standard component of the BERT architecture. This token is prepended to the input sequence and is designed to aggregate information across all time points during the model’s encoding process. Conceptually, the [CLS] token functions like an executive summary of a detailed report. While the model processes the entire sequence (the full report), it learns to distil the most critical information for the downstream classification task into the final hidden state of this single token. The classifier then uses this condensed summary vector to make its prediction (e.g., diagnosing ADHD), providing an efficient and powerful mechanism for sequence-level classification.

### Spatial attention module

#### Extracting and validating neurobiologically informed features from attention

While traditional functional connectivity (FC) methods based on statistical correlations often assume linearity and static relationships^34^, brain dynamics are inherently nonlinear and time-varying. Our framework aims to capture these complex interactions using a self-attention mechanism, as implemented in transformer models^35^.

The multi-head attention architecture is particularly well-suited for this task. By projecting the input into multiple representation subspaces, the model can learn diverse and multifaceted relationships between brain regions in parallel. In our model, each of the multiple attention heads generates a unique *N*_ROI_ × *N*_ROI_ attention matrix. These matrices highlight distinct patterns of inter-regional relationships that the model learns to prioritize for its predictions; for example, one head might focus on frontal-temporal relationships while another emphasizes occipital-parietal connections.

Crucially, we propose that these attention patterns represent a novel, task-optimized connectivity metric that is qualitatively distinct from traditional FC. While traditional FC captures static, linear correlations, which may include non-informative physiological noise, MBBN’s attention is a dynamic, non-linear weighting learned to solve a specific predictive task. Therefore, it is optimized to selectively capture inter-regional interactions that are salient for the prediction, while learning to suppress non-informative connections. To create a comprehensive summary of these learned connectivity patterns for a given fMRI scan, we compute an aggregate attention map by averaging the matrices from all heads. This final map represents the pairwise regional relationships that the model, in aggregate, deemed most salient for a given prediction.

Therefore, the goal of this analysis is two-fold. First, to establish that these task-optimized attention matrices represent a new and valid connectivity metric that is distinct from traditional FC. Second, to validate that this new metric captures neurobiologically meaningful and clinically relevant biomarkers. This fundamental distinction from FC is empirically supported by our supplementary analyses:

- The two metrics show distinct topological properties. In the raw signal, traditional FC exhibits clear modularity, whereas the attention matrix shows virtually none. However, after our frequency decomposition, robust modularity emerges only in the attention matrices (Supplementary Table 1).
- The two metrics capture complementary, frequency-specific information. While they show a strong negative correlation in the raw signal, this correlation becomes markedly weaker in the frequency-specific bands (Supplementary Table 2).

To validate the clinical relevance of this new metric, we use these aggregate attention maps in a group-level procedure. First, we identify which inter-regional patterns consistently differ between clinical and control groups (creating a ’group-difference attention map’). We then analyze these model-derived discriminative patterns to uncover novel, frequency-specific biomarkers of brain organization. This procedure allows us to demonstrate that our model has learned clinically relevant neural signatures that are distinct from, and complementary to, those found using conventional FC.

#### Spatial loss

The three frequency-divided components, separated by frequencies (*f*_1_, *f*_2_), exhibit more distinct differences in connectivity patterns^31^. To highlight these differences and enforce this specialization, we introduced a novel spatial loss term designed to encourage different spatial connectivity patterns for each frequency range. Conceptually, this loss function can be analogized to a rule that rewards a cartographer for creating three different, specialized maps of a city (e.g., a traffic map, a zoning map, and a topographical map) from the same satellite data, rather than three nearly identical general maps.

Specifically, the loss term maximizes the sum of L1 losses between attention matrices derived from different frequency ranges (equation (9)), thereby penalizing similarity. The use of the negative logarithm in the loss function further magnifies smaller differences between frequency bands, ensuring that even subtle distinctions are emphasized during training. This approach enables the model to learn unique spatial connectivity patterns for each frequency band, reflecting the potentially distinct contributions of these bands to brain activity. Moreover, this spatial loss term works in conjunction with other loss functions in the model to strike a balance between generalization and specificity.

6 $$Los{s}_{high-low}=Los{s}_{L1}(attma{t}_{high},attma{t}_{low})$$

7 $$Los{s}_{high-ultralow}=Los{s}_{L1}(attma{t}_{high},attma{t}_{ultralow})$$

8 $$Los{s}_{ultralow-low}=Los{s}_{L1}(attma{t}_{ultralow},attma{t}_{low})$$

9 $$Los{s}_{spatial}=-log(Los{s}_{high-low}+Los{s}_{high-ultralow}+Los{s}_{ultralow-low})$$

The negative logarithm amplifies subtle connectivity differences, ensuring the model learns distinct spatial patterns even when frequency bands show similar overall connectivity structures. We confirmed that this approach indeed derives neurobiologically plausible network properties (see Supplementary Materials 5).

### Communicability-based pretraining strategy

#### Masking strategy and pretraining loss

The goal of pretraining is to enable the model to learn the underlying structure of brain signal data in a self-supervised manner. To achieve this, we incorporated the concept of communicability into the pretraining process. A detailed definition of communicability used in this study is provided in Supplementary Materials 5.

High-communicability nodes act as critical hubs in brain networks, integrating information across distributed regions. Conceptually, our masking strategy can be analogized to understanding a social network by temporarily hiding its most central ’influencers’ and inferring their roles based on the activity of their connections. By masking these influential nodes during pretraining, we force the model to learn their functional roles through contextual inference, similar to masked language modeling but tailored to brain network topology.

Our pretraining strategy draws inspiration from masking techniques commonly used in language models to capture contextual relationships and data patterns^32^. We hypothesized that masking nodes with high communicability would encourage the model to infer the activity of these influential nodes based on the surrounding network dynamics. This approach highlights the role of high-communicability hubs in shaping the overall structure of resting-state networks and compels the model to learn the underlying rules of neural communication.

To validate this hypothesis, we compared three masking strategies:

- Masking nodes with high communicability.
- Masking nodes with low communicability.
- Randomly masking ROIs.

Detailed model performance results (Supplementary Materials 5) show that masking high-communicability nodes led to the most effective learning of the data structure, resulting in superior performance in downstream tasks.

**Fig. 5:**
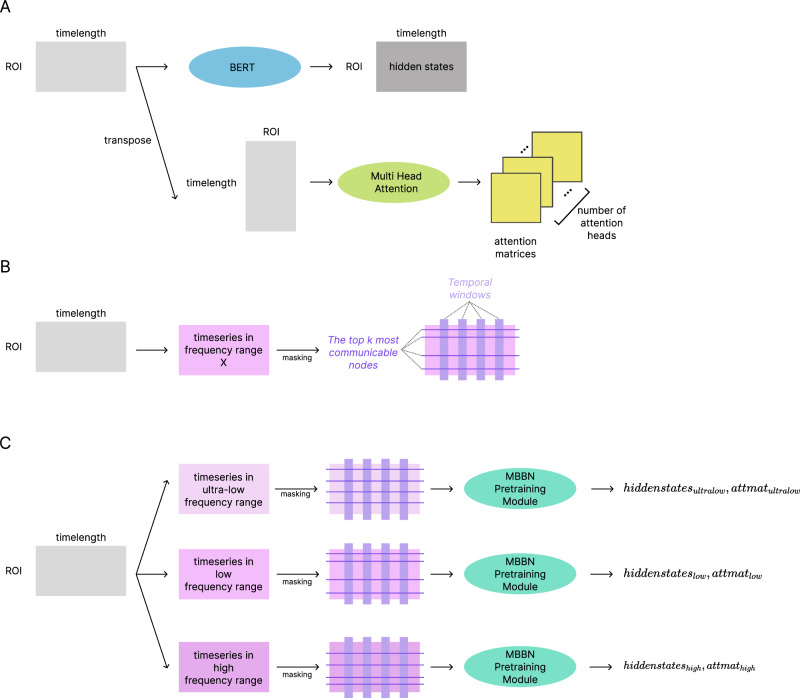
MBBN Pretraining Workflow. **A** Each frequency-divided time series is processed using the MBBN pretraining module, where the temporal module extracts hidden states using a BERT encoder, and the spatial module computes attention matrices via a multi-head attention mechanism. **B** Masking strategies: spatial masking nullifies the time series data of the top *k* nodes with the highest communicability scores, while temporal masking zeros out ROI signals within predefined time windows. **C** Pretraining is conducted separately for high, low, and ultralow frequency bands. Parameter sharing is applied in the temporal module but not in the spatial module. Outputs include hidden states and averaged attention matrices for each frequency range.

Pretraining involved two complementary masking strategies: temporal masking and spatial masking (Fig. 5). Spatial masking involved masking the time series data of the top *k* nodes with the highest communicability scores, where *k* is less than the total number of ROIs. Temporal masking, on the other hand, initialized all ROI signals to zero within predefined time windows. Both strategies were applied to each frequency band (ultralow, low, and high), denoted as *ν*.

The masking loss (*Loss*_mask_) was calculated as the sum of L1 losses between the original BOLD signals (*BOLD*_*ν*_) and their masked counterparts (*maskedBOLD*_*ν*_):

10 $$Los{s}_{{\mathrm{mask}}}={\sum }_{\nu }Los{s}_{L1}(BOL{D}_{\nu },maskedBOL{D}_{\nu })$$

The pretraining loss was the combination of the masking loss and the spatial loss (equation (9)), which enables the model to simultaneously learn node relationships and recover masked regions. *λ* employed in the pretraining loss function was maintained at the same value as that used in the total loss calculation (equation (5)) during from-scratch training.

11 $$Los{s}_{{\mathrm{pretrain}}}=Los{s}_{{\mathrm{mask}}}+\lambda \cdot Los{s}_{{\mathrm{spatial}}}$$

#### Temporal masking optimization

In temporal masking, rather than predicting a single time point, the model is tasked with predicting entire time windows. This strategy makes the task more challenging, encouraging the model to learn temporal dependencies more effectively. Masking was implemented by setting ROI signals to zero within predefined time windows.

To optimize temporal masking, we empirically tested various configurations by adjusting two parameters:

- *N*: the length of the masked time window.
- *k*: the distance between consecutive masked windows.

The best performance in downstream tasks was achieved with *N* = 20 and *k* = 1, where *N* ranged from 5 to 45 in multiples of 5, and *k* was an integer between 1 and 5.

#### Implementation details and dataset

Pretraining was conducted using the UK Biobank dataset, with a sequence length of 464. During fine-tuning, padding was applied to align shorter sequences (*τ*) to this fixed length, with padding added equally to both ends of the sequence ((464 − *τ*)/2). The outputs from the pretraining process, which include hidden states and attention matrices for each frequency band, were used to initialize the MBBN model for downstream tasks. Figure 5 illustrates the pretraining workflow, including the temporal and spatial masking procedures. Temporal masking is represented by lavender rectangles, while spatial masking is depicted with purple lines.

#### Interpretability

MBBN computes attention matrices between ROIs across different frequency ranges. These matrices serve as estimates of functional connectivity, making it crucial to analyze individual connections to understand their contributions to phenotype classification. This analysis offers insights into the neural mechanisms underlying the phenotype of interest.

Traditional attribution methods are not directly applicable to this model, as the relationship between attention matrices and target values is indirect. To address this challenge, we developed a GradCAM^36^-inspired approach to identify frequency-specific connections driving psychiatric classification. This method highlights connections where gradient magnitude (importance) aligns with attention strength (activation), revealing clinically relevant biomarkers.

12 $$Heatma{p}_{k}=\frac{\partial {Loss}_{{\mathrm{spatial}}}}{\partial {f}_{k}(inpu{t}_{k})}\odot {f}_{k}(inpu{t}_{k})$$

GradCAM typically highlights important regions by computing the gradient of the loss for the target class and weighting it by the output feature maps. Similarly, we compute heatmaps by multiplying the activation values (attention matrices) with their respective gradients, as described in Equation (12). This method highlights specific functional connectivity patterns that are relevant to mental disorders. In Equation (12), *f* refers to the spatial attention module, *Loss*_spatial_ corresponds to the loss term from Equation (9), *k* represents the frequency range (high, low, ultralow), and *input*_*k*_ denotes the fMRI BOLD data for frequency range *k* ($Heatma{p}_{k}\in {{\mathbb{R}}}^{({N}_{ROI}\times {N}_{ROI})}$).

To interpret the model’s predictions, we selected the best-performing model based on the validation dataset and applied it to the test dataset. Heatmaps were generated for true positive subjects. Analysis for false positive and false negative subjects was conducted to validate the method. For inter-group comparisons, we conducted *t* tests to identify statistically significant connections (*p* < 0.05). For multiple comparison correction, we applied false discovery rate (Benjamin–Hochberg method) to control for Type I errors. Then we calculated the effect size using Cohen’s *d*^37^. The sign of the t-statistic indicated which group had a higher attribution score for a given connection. For example, in the classification of ADHD using the ABCD dataset, a positive t-statistic suggested that a connection contributed more significantly to the HC group than to the ADHD group.

Statistically significant connections (*p* < 0.05) and regions were analyzed, but the number of connections was too large to handle effectively. To address this, we selected only a few top connections based on the absolute values of Cohen’s *d* for further evaluation of their impact. An exhaustive list of all significant connections can be found in Supplementary Materials 5.

##### Stability and consistency of Interpretability analysis

To assess the stability and consistency of the connections identified by our GradCAM-inspired approach, we performed a rigorous bootstrapping analysis. We created 100 subsamples by randomly selecting 100 subjects from the test set for both the ADHD and ASD classification tasks. For each subsample, we repeated the group comparison and identified the top 100 most discriminative connections based on effect size (Cohen’s *d*).

The consistency of the identified connections across these subsamples was quantified using the Jaccard Index, which measures the similarity between sets. The analysis revealed a high degree of stability for the top-ranked connections in all frequency bands:

- High-frequency band: Jaccard Index = 0.940 ± 0.101
- Low-frequency band: Jaccard Index = 0.900 ± 0.078
- Ultralow frequency band: Jaccard Index = 0.920 ± 0.035

These high Jaccard Index values, consistently above 0.9, indicate that the discriminative connectivity patterns highlighted by our model are highly robust and not driven by a specific subset of subjects. This confirms that the identified biomarkers are stable features of the clinical groups.

#### Performance comparison with baseline models

To evaluate the effectiveness of MBBN in learning spatiotemporal dynamics, we compared its performance with baseline models, including functional connectivity-based models (XGBoost^38^, BNT^5^) and temporal dynamics models (BolT^6^, vanilla BERT^32^). These models were selected based on their relevance to brain dynamics interpretation, either through static functional connectivity or sequential processing of temporal data. While functional connectivity-based models primarily rely on static representations, temporal dynamics models capture sequential patterns but do not explicitly integrate spatial relationships. MBBN addresses this gap by combining both spatiotemporal characteristics using its frequency-dividing encoder and spatial attention module. Given that MBBN is an extension of vanilla BERT with additional architectural enhancements, a direct comparison between the two provides a robust evaluation of these modifications. While MBBN demonstrated superior performance across several downstream tasks, including the ABCD ADHD classification task, with significant improvements in accuracy and AUC scores, not all tasks showed a statistically significant advantage. Detailed performance comparison is described in Supplementary Materials 5.

To ensure a fair evaluation, we conducted statistical tests (paired *t* tests) to compare the performance of MBBN with baseline models on each task. The *p* values for these comparisons indicated that, although MBBN did not outperform all baseline models in every metric, the differences were not statistically significant (*p* > 0.05) in cases where baseline models showed slightly better performance. These results suggest that MBBN encodes information at a level comparable to or better than existing models while providing the additional advantage of capturing spatiotemporal dynamics, which baseline models are unable to incorporate.

### Statistics and reproducibility

#### Statistical analysis

For binary classification tasks (e.g., sex, ADHD, ASD, MDD), we report the AUROC, balanced accuracy, sensitivity, specificity, and F1-score. The optimal classification threshold was determined on the validation set by maximizing the geometric mean (G-mean) of sensitivity and specificity, and this threshold was applied to the held-out test set. For regression tasks (e.g., fluid intelligence, MDD severity), we report the MAE, mean squared error (MSE), normalized MSE (NMSE), and coefficient of determination (*R*^2^). All metrics were computed using scikit-learn (v1.6.1). To assess robustness across data splits and initialization, experiments were repeated with three random seeds. Results are reported as mean  ± standard error of the mean (SEM) across seeds. Statistical significance of model comparisons was evaluated using paired or independent two-sample *t* tests where appropriate. All statistical tests were two-sided.

#### Data splitting and sample sizes

Data were split into training (70%), validation (15%), and test (15%) sets. For classification tasks, stratified sampling was used to preserve the class distribution in each split. For regression tasks, random splitting was applied. Subject-level splits were generated once per seed and saved to disk, ensuring identical splits across runs with the same seed. Subjects with missing target values or invalid imaging data (e.g., zero-variance ROIs) were excluded before splitting.

##### Reproducibility

Random seeds were fixed for PyTorch, NumPy, Python’s random module, and CUDA at the start of each experiment. The seed value is passed as a command-line argument and determines both the data split and model initialization. All hyperparameters and experiment configurations are logged in each experiment folder. Training was performed using PyTorch 2.6.0 with CUDA 12.4, and mixed-precision (AMP) was enabled for efficiency. The computational environment uses Python 3.10 with key dependencies: nibabel 5.3.2, nitime 0.11, scikit-learn 1.6.1, and pandas 2.3.2. A conda environment specification is provided in environment.sh for full reproducibility.

##### Software and data availability

The MBBN codebase and environment setup instructions are available in the project repository. Pre-computed data splits are stored in the splits/directory. fMRI data were preprocessed and parcellated according to the HCP-MMP1 (360 parcels) or Schaefer (400 parcels) atlases before model input.

### Reporting summary

Further information on research design is available in the Nature Portfolio Reporting Summary linked to this article.

## Discussion

Here we present MBBN, a novel deep learning framework that integrates biologically grounded principles with frequency-aware self-attention to model spatiotemporal brain dynamics. By leveraging scale-free and multifractal properties of neural signals, MBBN captures essential patterns of brain organization that are often overlooked by conventional transformer-based models. Additionally, MBBN employs communicability-based masking and frequency-aware self-attention to capture biologically meaningful connectivity patterns across multiple frequency ranges, enabling accurate and interpretable predictions of cognitive and clinical outcomes.

Unlike previous approaches that rely on broadband or static representations^5,6^, MBBN applies a frequency decomposition strategy to encode neural oscillations as discrete features, enabling a richer characterization of dynamic connectivity patterns. This frequency-resolved approach is particularly critical given that neural communication is mediated by distinct oscillatory dynamics across frequency bands, with high-frequency oscillations supporting local processing^39^ and low-frequency rhythms facilitating long-range integration^40^. Traditional broadband or narrowband analyses often obscure these nuanced patterns by averaging across spectrally diverse signals, potentially masking functionally specific effects. The MBBN framework overcomes these limitations by capturing such frequency-specific properties, allowing for a more precise and physiologically grounded understanding of brain network dynamics. Furthermore, our communicability-based^41,42^ masking method ensures that topological structures within brain networks are embedded into learned representations, improving both robustness and interpretability.

Importantly, MBBN-derived connectivity patterns complement rather than replicate traditional functional connectivity. While moderate anticorrelation existed at the broadband level, frequency-specific analyses revealed unique network dynamics (Supplementary Table 2), confirming that MBBN captures neurophysiologically distinct information critical for clinical interpretation.

The biological plausibility of MBBN is further supported by its incorporation of the scale-free property, characterized by a power-law exponent (*β*), which differentiates brain states and regions based on their temporal correlation profiles^2,43–47^. Variations in *β* values are associated with functional specialization and pathological alterations^16,17,48,49^, underscoring the importance of integrating these intrinsic properties into computational models. By incorporating communicability-based masking, MBBN systematically accounts for graph-structured brain data, addressing the limitations of random masking strategies^50,51^ and enhancing understanding of network-level interactions^41,52^.

When applied to graph-structured functional brain data, random masking fails to consider node importance and the non-Euclidean nature of the data^50,51^, leading to uncertainties in reconstructing masked information. MBBN overcomes these limitations through a communicability-based masking strategy, which quantifies information flow across all possible network paths and emphasizes the role of high-communicability hubs in shaping network dynamics^41,52^. By selectively masking these critical hubs and reconstructing their activity through interactions with surrounding nodes, MBBN learns structural dependencies intrinsic to neural systems, enhancing both interpretability and robustness. This approach establishes MBBN as a foundational framework for advancing neuroimaging analysis.

Across diverse cohorts-including UK Biobank, ABCD, and ABIDE-MBBN consistently demonstrated strong predictive performance, capturing neural organizational principles across age groups. Unlike many existing models that are validated only on narrow demographics, MBBN successfully generalized across developmental stages, revealing its potential as a neuroimaging tool with strong generalizability across child, adolescent, and adult cohorts.

Our frequency-specific analyses uncovered distinct connectivity disruptions in ADHD and ASD. In ADHD, we observed attenuated high-frequency connectivity in fronto-sensorimotor networks, which may underlie core symptoms such as inattention and executive dysfunction^53,54^. At lower frequencies, altered sensory and language processing circuits were evident^55–58^, and in the ultralow-frequency range, both hypo- and hyperconnectivity patterns suggested inefficient compensatory mechanisms, particularly involving the parahippocampal and cingulate regions^59–63^.

A striking finding was the frequency-dependent shift in Area OP2-3-VS (Opercular parts of Areas 2 and 3 and adjacent Ventral Somatosensory/Sylvian region) connectivity in ADHD. This region consistently emerged as a central node across different frequency bands, suggesting it may serve as a dynamic hub that potentially coordinates neural communication based on oscillatory states. Rather than subserving a fixed function, Area OP2-3-VS appears to assume context-dependent roles, possibly regulating cross-frequency information flow or facilitating inter-network switching-mechanisms that could be critical for coherent cognitive function. In ADHD, disruptions in these putative regulatory processes may contribute to frequency-specific connectivity alterations and the disorder’s heterogeneous symptomatology, underscoring the potential importance of spectral dynamics in characterizing functional hubs.

In ASD, high-frequency hubs involving orbitofrontal and somatosensory circuits were prominent, consistent with known deficits in emotional and social processin^64–67^. At low frequencies, the posterior frontopolar cortex emerged as a key integrative hub; disruptions here may impair the coordination of memory, decision-making, and multisensory integration essential for goal-directed behavior^68–71^. Interestingly, ultralow-frequency analyses revealed heightened connectivity between the temporo-parietal junction and prefrontal regions, possibly reflecting compensatory cognitive strategies or atypical resource allocation during social cognition tasks^72–75^.

Collectively, these results illustrate that MBBN not only outperforms prior models in predictive capacity but also provides interpretable biomarkers aligned with known neurodevelopmental patterns. The model’s clinical relevance is first established by its ability to replicate and validate previously identified neurobiological markers. For instance, in ADHD, our model identified attenuated high-frequency connectivity within fronto-sensorimotor networks, while in ASD, it highlighted disruptions in high-frequency hubs involving the orbitofrontal and somatosensory circuits, aligning directly with well-documented deficits in emotional processing and social cognition.

Beyond this important validation, the unique architecture of MBBN enables the generation of novel, testable hypotheses by revealing the frequency-specific nature of these disruptions. The first novel insight relates to ADHD, where we identified Area OP2-3-VS as a dynamic hub whose connectivity patterns shift depending on the oscillatory state. This leads us to propose that a central mechanism of the disorder may be a disruption in the coordination of information flow, orchestrated by this hub’s inability to flexibly manage frequency-dependent network communication. Secondly, our model offers a significant refinement to the hyperconnectivity theory in ASD. We discovered that the heightened connectivity between the temporo-parietal junction and prefrontal regions was not a broadband phenomenon but was predominantly observed in the ultralow-frequency band. This novel, frequency-specific finding suggests that atypical neural resource allocation in ASD is tied to specific, slow-timescale dynamics, providing a more precise biomarker than previously understood. By aligning transformer-based modeling with core neuroscientific principles, MBBN offers a scalable and biologically plausible framework for advancing our understanding of individualized brain dynamics.

While MBBN offers notable methodological advancements, we acknowledge several limitations that offer avenues for future work. First, the communicability-based masking is computationally demanding, and future iterations could investigate more efficient approximations like those based on polynomial expansions or random walks^42^. Furthermore, our frequency decomposition operates on standard, pre-filtered fMRI signals to ensure comparability with established research. Consequently, the model identifies meaningful sub-bands within this conventional spectral window, not from the complete raw signal, a scope that future work could expand upon. Second, a limitation pertains to the model’s modest performance on regression tasks. We attribute this primarily to the nature of the regression targets (e.g., scores from single questionnaires or behavioral tests), which have an inherently lower signal-to-noise ratio compared to the robust diagnostic labels used for classification. This data-level challenge is likely compounded by the intrinsic bias of transformers to learn non-smooth functions, which may be less optimal for capturing the subtle variations in continuous phenotypes^76^. Third, the spatial loss weight *λ* was tuned via grid-search. While a more principled approach would be a dynamic weighting scheme, our experiments with a gradient-norm-based method showed sensitivity to noisy initial gradients and did not consistently outperform a well-tuned fixed *λ*. Thus, developing more robust dynamic heuristics remains a key challenge for future work. Finally, while the model proved robust across cohorts with heterogeneous acquisition parameters, further validation on more diverse populations, including elderly and neurodegenerative cohorts, is required. While our evaluation shows that MBBN’s advantage is less pronounced for regression targets, we emphasize that our core contribution is methodological: a frequency-resolved, biologically grounded framework that can serve as a foundation for future models to better capture continuous outcomes. At the same time, MBBN exhibits strong and practically relevant performance on categorical phenotypes, achieving high AUROCs (e.g., sex classification in ABCD: 0.916; UKB: 0.980; ASD classification in ABIDE: 0.810). This balance clarifies our positioning: MBBN is a methodological advance that already delivers high utility for clinical differentiation tasks, while providing a principled basis for subsequent extensions aimed at improving regression performance.

Looking ahead, our primary goals are to enhance the framework’s accessibility and expand its scientific applications. To mitigate the significant computational cost of pretraining, we are releasing our trained models to the public, allowing researchers with limited infrastructure to bypass this intensive step and apply our framework directly via efficient fine-tuning. Furthermore, we plan to develop more lightweight versions of MBBN through architectural simplification and knowledge distillation, creating student models suitable for resource-constrained clinical settings. In addition, future research could explore architectural enhancements-—such as Smoothness Regularization or a Hybrid Architecture-to further improve regression performance, complementing the model’s strong classification capability. A key future direction is extending MBBN from resting-state to task-evoked fMRI. While the scale-free assumption for our frequency decomposition is most stable during rest, we hypothesize that the Multi-Band Attention mechanism is inherently well-suited to capture transient, task-driven neural dynamics. For instance, the model could learn to detect task-related power shifts in specific frequency bands-such as increased high-frequency activity in the frontoparietal network during a working memory task-to classify cognitive states and extract dynamic, task-evoked connectivity. Equally important will be validating MBBN’s applicability across diverse patient populations to move toward broader clinical utility. We are actively pursuing this line of research to broaden our framework’s applicability and provide deeper insights into the neural correlates of cognition.

In conclusion, MBBN establishes a new standard for interpretable, frequency-resolved brain modeling. By integrating neurophysiological principles with transformer architectures, it offers both predictive power and mechanistic insights, laying a solid foundation for future data-driven applications in clinical and developmental neuroscience.

## Supplementary information

Supplementary Material

Description of Additional Supplementary Files

Supplementary Data

Reporting Summary

Transparent Peer Review file

## Data Availability

The UKB, ABCD, and ABIDE datasets are publicly available to researchers.

## Code availability

The code is available on Github (https://github.com/Transconnectome/MBBN).
