# Supplementary material for: Learning brain dynamics across distinct scaling regimes reveals psychiatric signatures
Source: Commun Biol. 2026 May 8;9:963. doi: 10.1038/s42003-026-10011-7 (PMC13369815; doi:10.1038/s42003-026-10011-7)
Supplement: Supplementary file 1 — Supplementary Material [file 42003_2026_10011_MOESM1_ESM.pdf]

Pretraining

Communicability

Communicability encompasses not only the shortest paths but also other types of walks between nodes p and q within a network. A network can be represented as a graph $G = (V, E)$, where V represents nodes and E represents the edges connecting the nodes. Let us denote the number of nodes as n and the number of edges as m. The adjacency matrix of the graph is $A(G) = A$, where A_{ij} takes a value of 1 if nodes i and j are connected and 0 otherwise. The communicability between nodes p and q in the network, denoted as G_{pq}, incorporates eigenvalues and eigenvectors of the adjacency matrix. Let $\lambda_1 \geq \lambda_2 \geq \cdots \geq \lambda_n$ be the eigenvalues in non-increasing order, and let $\phi_j(p)$ represent the p-th element of the j-th orthonormal eigenvector corresponding to eigenvalue λ_j. Then, communicability is calculated as follows:

$$G_{pq} = \sum_{j=1}^{\infty} \phi_j(p)\phi_j(q)e^{\lambda_j} \tag{1}$$

Communicability provides a measure of how efficiently information flows between nodes by considering not just direct connections but also indirect pathways weighted by their importance. This property makes communicability particularly suitable for identifying high-influence nodes in brain networks, which are then used in our pretraining masking strategy.

Effective Pretraining: Finding the Optimal Number of Epochs

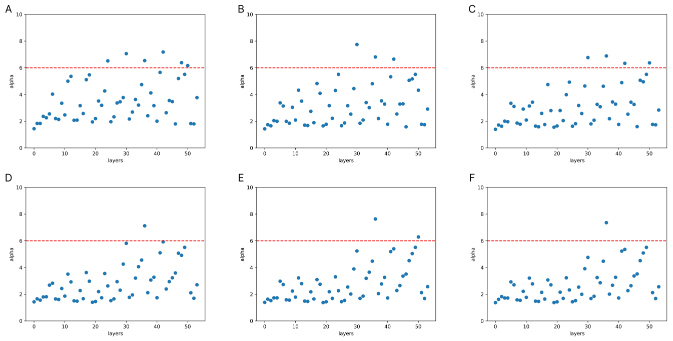

Supplementary Figure 1 Alpha Value Changes by Pretraining Epochs (HCPMMP1 Atlas, Epochs: 100-600)

Determining the optimal number of epochs for pretraining is crucial for creating an effective pretraining strategy. We utilized weightwatcher ([28]), a diagnostic tool based on traditional statistical learning theory and statistical mechanics, to identify well-trained models. Weightwatcher analyzes the norms of the weight matrix W and

parameters of power-law (PL) fit on the eigenvalues of W to assess training quality without requiring data.

According to the Heavy-Tailed Self-Regularization (HT-SR) Theory, lower PL exponent (α) values indicate stronger implicit self-regularization, leading to better generalization. Consistently low α values across layers allow effective correlation flow, enabling the gradual transformation of data correlations through each layer. As shown in previous work, layers with α values below 6 are deemed well-trained, while those exceeding 6 are considered poorly trained.

In the following figures, the x-axis represents the layers, and the y-axis represents the α values. The red dotted line marks the threshold of $\alpha = 6$. The number of layers with α values above 6 converges to a single layer after epoch 500 (Supplementary Figure 1). We hypothesize that the epoch at which the pattern of layers with α values exceeding 6 stabilizes marks the optimal pretraining epoch. In our monitoring process, we utilized WeightWatcher to analyze the pretrained model at intervals of 100 epochs. We identified the critical epoch n at which the number of layer with $\alpha > 6$ converges. Subsequently, we conducted a series of fine-tuning experiments at 10-epoch intervals between epochs $n - 100$ and n to empirically determine the optimal epoch for model performance. In HCPMMP1 atlas, n was 600.

Selecting these epochs ensures that most layers in the model achieve sufficient self-regularization, improving overall generalization performance during fine-tuning.

Interpretation of Network Properties

Measure	Modularity		Small-worldness Parameters					
	FC	Attention	FC			Attention		
			γ	λ	σ	γ	λ	σ
High frequency	0.0278	0.0576	4.7683	1.4239	3.3487	9.3960	0.9220	10.1913
Low frequency	0.0251	0.0701	4.8803	1.4140	3.4514	5.6272	0.9953	5.6539
Ultralow frequency	0.0350	0.0991	4.8600	1.4794	3.2850	8.3929	0.9229	9.0941
Raw (Non-dividing)	0.0630	< 0.0001	7.7804	1.6143	4.8197	5.9527	0.9254	6.4329

Supplementary Table 1 Comparison of Network Properties between FC and Attention Matrix

Our analysis of network properties provides strong evidence that frequency-specific attention matrices capture meaningful connectivity patterns that differ from traditional functional connectivity (FC).

- Modularity quantifies the strength of division of a network into modules or communities. It measures how well a network can be decomposed into distinct subgroups, with higher values indicating more distinct community structures where connections within communities are denser than connections between communities. Formally, it is defined as the fraction of edges that fall within given communities minus the expected fraction if edges were distributed at random while preserving node degrees.

- Small-worldness parameters characterize the topological properties of a network that exhibits both high clustering and short path lengths:
  - Gamma (γ): The clustering coefficient ratio comparing the observed network to a random network with the same number of nodes and edges. Higher values indicate greater local connectivity and clustering, reflecting more efficient local information processing.
  - Lambda (λ): The characteristic path length ratio comparing the observed network to a random network. Values closer to 1 indicate efficient global integration and information transfer across the network.
  - Sigma (σ): The small-worldness index calculated as γ/λ. Values significantly greater than 1 indicate that the network exhibits small-world properties, characterized by high local clustering and relatively short path lengths. Small-world networks are considered optimal for information processing, combining efficient local processing with global integration.

These network metrics provide quantitative measures for assessing the topological organization of brain networks and are widely used in neuroimaging studies to characterize functional and structural connectivity patterns. Several key observations can be made from Supplementary Table 1:

1. Frequency-dependent modularity: The attention matrices exhibit distinct modularity and small-worldness patterns across frequency bands (high, low, and ultralow frequencies). A clear trend of increasing modularity with decreasing frequency ($0.0576 \rightarrow 0.0701 \rightarrow 0.0991$) is observed. This pattern aligns with findings from [77], which demonstrated that resting-state BOLD signals display frequency-specific network architecture, where networks that are finely subdivided in lower frequency bands become integrated into fewer networks in higher frequency bands rather than reconfigured. Such evidence supports the observation that modularity values are higher in lower frequency bands, consistent with our results.
2. Contrasting patterns with FC: While FC exhibits its highest modularity in the raw (unfiltered) condition (0.0630), attention-based connectivity shows essentially no modularity in the raw condition (< 0.0001). This striking difference suggests that our attention mechanism captures frequency-specific network organization that is lost when analyzing the full frequency spectrum together.
3. Small-worldness characteristics: The small-worldness parameters (γ, λ, and σ) reveal distinct network architectures between FC and attention-based connectivity. Notably, attention matrices consistently show higher clustering (γ) and small-worldness (σ) values across frequency bands compared to FC, while maintaining more optimal path length properties (λ closer to 1). This indicates that attention-based connectivity may better preserve the small-world architecture that is characteristic of brain networks.
4. Frequency-specific information: Raw data without frequency decomposition fails to exhibit meaningful modularity in the attention matrix, whereas frequency-decomposed data reveals distinct modular structures. These findings suggest effective coupling between the spatial module and frequency-dividing strategy, enabling the extraction of neurobiologically relevant patterns. This observation underscores

the importance of frequency-specific analysis when employing attention-based connectivity measures to characterize brain network organization.

These findings support our claim that frequency-specific attention matrices represent a valid and interpretable new form of connectivity measure, capturing network properties that complement traditional FC measures.

Correlation Analysis Between FC and Attention-Based Connectivity

To further investigate the relationship between functional connectivity (FC) and our proposed attention-based connectivity measure, we conducted correlation analyses across different frequency bands. Spearman correlation measures the monotonic relationship between two variables by evaluating the rank order association, making it robust to outliers and nonlinear relationships. Kendall tau is a similar non-parametric measure that assesses the ordinal association between two variables by analyzing the number of concordant and discordant pairs, providing a measure of agreement in rankings. The results are presented in Table 2.

Frequency Band	Spearman Correlation	Kendall Tau
Raw (Non-dividing)	-0.6081	-0.4367
High frequency	-0.0952	-0.0700
Low frequency	-0.1459	-0.1072
Ultralow frequency	-0.1973	-0.1451

Supplementary Table 2 Correlation Analysis Between FC and Attention Matrix

The correlation analysis reveals several important insights:

1. Complementary information: The strong negative correlation between FC and attention-based connectivity in the raw condition (Spearman $\rho = -0.6081$, Kendall $\tau = -0.4367$) indicates that these two measures capture fundamentally different aspects of brain connectivity. This negative correlation suggests that our attention-based connectivity is not merely replicating traditional FC but providing complementary information.
2. Frequency-specific patterns: When examining frequency-specific bands, the correlations become considerably weaker (ranging from approximately -0.10 to -0.20), suggesting that each frequency band captures unique connectivity patterns.
3. Methodological validity: The consistent negative correlations across all frequency bands, albeit weaker in the frequency-specific analyses, indicate that our attention mechanism systematically extracts connectivity information that differs from traditional FC. This suggests that attention-based connectivity represents a valid new form of connectivity measure with its interpretable meaning.

These correlation findings, combined with the network property analysis, provide strong evidence that our frequency-specific attention-based connectivity measure captures meaningful and interpretable connectivity patterns that complement traditional FC analysis. The method does not simply maximize differences between frequencies but extracts biologically relevant connectivity information specific to each frequency band.

Test-Retest Reliability of Frequency-Specific Attention Matrices

To assess the temporal stability of our model's derived biomarkers, we performed a test-retest reliability analysis on the frequency-specific attention matrices. Due to the unavailability of UK Biobank test-retest data, we utilized the resting-state fMRI data from the Human Connectome Project (HCP) dataset, which includes two separate runs (run 1 and run 2) for each participant, serving as an excellent proxy for evaluating short-term reliability.

We fine-tuned the MBBN model on a sex classification task using the HCP dataset. After training, we extracted the subject-level attention matrices for each of the three frequency bands (high, low, ultralow) from both run 1 and run 2 of the test set. To evaluate the consistency of these matrices over time, we calculated the Intraclass Correlation Coefficient (ICC), using a two-way mixed-effects model for absolute agreement (ICC(A,1)). The analysis was conducted separately for male and female subjects to ensure reliability was not sex-dependent.

The results demonstrated high to excellent test-retest reliability across all conditions. For both male and female participants, the ICC values for the attention matrices in the high, low, and ultralow frequency bands were all greater than 0.95. This high level of temporal stability confirms that the frequency-specific connectivity patterns captured by MBBN are robust and consistent, strengthening their validity as reliable biomarkers for downstream clinical and cognitive prediction tasks.

Confounds Analysis

We assessed whether major potential confounds—age, sex, head motion (mean FD), and multi-site acquisition—could bias the reported effects in the ABCD cohort. We (i) compared group characteristics before and after propensity score matching (PSM) and (ii) tested associations between the learned knee frequencies (f_1, f_2) and each confound.

Confound analyses and propensity score matching (details)

We evaluated whether sex, age, head motion (mean FD), and site (22 acquisition locations) were associated with the learned knee frequencies (f_1, f_2). For sex (binary) and site (22 levels), factors were entered as categorical predictors; age and mean FD were modeled as continuous covariates. Linear models and AN(C)OVA were fit with site as a factor when appropriate. Multiplicity was controlled using false discovery rate (FDR; Benjamini–Hochberg), and we report adjusted p-values for all tests. To mitigate

residual confounding in downstream classification, we constructed a propensity score–matched subsample via 1:1 nearest-neighbor matching on sex, age, mean FD, and site (logistic propensity model; caliper = 0.2 SD of the logit; no replacement). Covariate balance was assessed using standardized mean differences (SMD), with $|\text{SMD}| < 0.10$ indicating acceptable balance; Love plots and hypothesis tests are provided for reference. The ADHD classification model was then re-estimated on the matched set, and performance was compared to the pre-matching baseline using a paired t-test.

Pre-matching group comparison

Sample sizes before matching were $N_{\text{Group 0}} = 3032$ and $N_{\text{Group 1}} = 2334$. Group differences indicated substantial imbalance for motion, sex, and site (Table 3).

Variable	Test statistic	p-value
Age	$t = -0.20$	$p = 0.8418$
Mean FD (rsfMRI)	$t = -11.92$	$p = 0.0000$
Sex	$\chi^2 = 226.50$	$p = 0.0000$
Site (22 levels)	$\chi^2 = 112.39$	$p = 0.0000$

Supplementary Table 3 Pre-matching group comparison (ABCD).

Propensity score matching (PSM) and post-matching balance

We constructed a matched subsample jointly balancing sex, age, mean FD, and site (1:1 nearest-neighbor; caliper = 0.2 SD on the logit; no replacement). Covariate balance was evaluated via standardized mean differences (SMD) and hypothesis tests (Table 4); SMD for all matched covariates was < 0.10. As enforced by the matching specification, sex and site achieved near-exact balance; age and motion showed no residual differences.

Variable	Test	p-value
Mean FD (rsfMRI)	two-sample t-test	0.9869
Age	two-sample t-test	0.4545
Sex	(balanced by design; SMD < 0.10)	—
Site (22 levels)	(balanced by design; SMD < 0.10)	—

Supplementary Table 4 Post-matching group comparison (ABCD matched subsample).

Associations between knee frequencies and confounds

We then tested associations between f_1/f_2 and each confound (Table 5). Sex was associated with f_1 only (not f_2), site effects were present for both knees, whereas age and head motion showed no significant associations. These results suggest a potential sex-linked physiological contribution at lower frequencies and indicate that age and motion are unlikely to drive the spectral knees.

Predictor	p-value for f_1	p-value for f_2
Sex (binary)	0.0001	0.8641
Age (months)	0.16564	0.5921
Mean FD (rsfMRI)	0.79716	0.48531
Site (22 levels; ANOVA)	0.0000	0.0000

Supplementary Table 5 Associations of knee frequencies with confounds (ABCD).

Remark on downstream analyses

In the matched subsample, ADHD classification performance numerically increased (AUROC 0.633 $\rightarrow$ 0.651); however, the difference was not statistically significant in our paired comparison ($p = 0.3742$). This indicates that the disorder effect is robust to demographic, motion, and multi-site factors under stringent covariate balancing.

Ablations

The performance of the model when provided with timeseries data of a single frequency range.

	ABCD sex classification	
Data	AUROC ↑	Accuracy ↑
Z-scored original signal	0.864 [0.851, 0.877]	0.777 [0.759, 0.795]
High Frequency	0.888 [0.866, 0.909]	0.797 [0.763, 0.832]
Low frequency	0.884 [0.870, 0.898]	0.784 [0.751, 0.817]
Ultra-low Frequency	0.870 [0.840, 0.898]	0.779 [0.739, 0.819]
Divided Frequencies	0.916 [0.903, 0.929]	0.844 [0.826, 0.861]

Supplementary Table 6 Results of BERT models trained with single frequency range in the timeseries data extracted by HCP-MMP1 atlas. Performance is reported as mean [95% Confidence Interval]

The core innovation of our model lies in its ability to encode and integrate information from distinct frequency bands. To deconstruct the sources of this performance

improvement, we conducted an ablation study to answer two key questions: (1) Is the simple act of filtering into any single band beneficial compared to using the raw signal? (2) Is there a synergistic benefit to integrating all three bands simultaneously?

First, we compared models trained on single frequency bands against a model trained on the unfiltered, Z-scored original signal. As shown in Supplementary Table 5, using any single frequency band yielded a notable performance improvement over the original broadband signal. For instance, the model using only the high-frequency band achieved an AUROC of 0.888, surpassing the 0.864 AUROC of the model using the original signal. This indicates that the process of isolating specific frequency ranges is beneficial in itself, likely by removing noise and emphasizing more informative oscillatory patterns.

Second, we analyzed the crucial impact of integrating all three frequency bands within our multi-view framework. The full model ('Divided Frequencies') achieved an AUROC of 0.916, significantly outperforming the best-performing single-band model (High Frequency, AUROC: 0.888). This result demonstrates a clear synergistic effect, where the model leverages complementary information from ultralow, low, and high frequency bands simultaneously. This confirms that the substantial performance gain of our framework is not merely an artifact of filtering, but a direct result of our multi-band modeling approach that integrates information across different neural timescales.

Treating fMRI signal as a sequence of discrete words.

Model	ABCD sex classification AUROC ↑	Accuracy ↑
MBBN with conv	0.835 [0.813, 0.858]	0.748 [0.745, 0.751]
MBBN without conv	0.916 [0.903, 0.929]	0.844 [0.826, 0.861]

Supplementary Table 7 Results of BERT models with a convolutional layer before BERT model. Performance is reported as mean [95% Confidence Interval].

Determining whether fMRI data should be treated as continuous variables, akin to speech, or discrete sequences, similar to natural language, is a critical design choice. Given the high sample rate of fMRI signals, we hypothesized that they resemble written sentences more closely than speech signals.

To evaluate this, we introduced convolutional layers before feeding the input into the model, inspired by speech recognition frameworks such as Wav2Vec [33]. Table 5 reveals that treating fMRI signals as discrete word sequences yields superior performance compared to processing them as continuous variables. This result highlights the effectiveness of viewing fMRI signals as structured sequences analogous to natural language.

Dividing Strategy	ABCD sex classification	
	AUROC ↑	Accuracy ↑
No division	0.864 [0.851, 0.877]	0.777 [0.759, 0.795]
Random 2 Freq	0.903 [0.891, 0.916]	0.818 [0.807, 0.829]
Lorentizan-based 2 Freq	0.903 [0.886, 0.920]	0.805 [0.773, 0.836]
Random 3 Freq	0.900 [0.883, 0.918]	0.803 [0.777, 0.828]
Wavelet & KNN	0.905 [0.886, 0.924]	0.811 [0.747, 0.875]
Canonical	0.911 [0.899, 0.923]	0.808 [0.757, 0.858]
Ours	0.916 [0.903, 0.929]	0.844 [0.826, 0.861]

Supplementary Table 8 Performance comparison of MBBN on the ABCD sex classification task using different frequency division strategies. Our proposed theory-driven approach ('Ours') is compared against baselines with no frequency division, random divisions, a wavelet-based adaptive method, and conventional fixed canonical bands. Performance is reported as mean [95% Confidence Interval].

Frequency-dividing strategies.

To validate our theory-driven frequency division strategy, we systematically compared its performance against several alternative approaches. As detailed in Supplementary Table 8, our proposed method ('Ours') achieved the highest AUROC (0.916) and Accuracy (0.844) on the ABCD sex classification task.

Notably, our approach outperformed not only baseline models using no frequency division but also other data-driven techniques, such as a wavelet-based method ('Wavelet & KNN'). Furthermore, it demonstrated a clear advantage over conventional, literature-based fixed bands ('Canonical'), underscoring the benefit of our individualized, scale-free approach. This comprehensive comparison confirms that defining three distinct frequency bands using our Lorentzian and spline-based method provides a more powerful and informative representation for predicting phenotypes from fMRI time-series data.

Sensitivity Analysis of Frequency Band Definitions

To assess the robustness of our model to minor variations in the data-driven frequency cutoffs, we conducted a sensitivity analysis. We systematically perturbed the empirically derived knee frequencies (f_1 and f_2) by ±5% and ±10% and re-evaluated the model's performance on the ABCD sex classification task. The four perturbation conditions were:

- Wider: The frequency band was widened by shifting f_1 down by 10% (or 5%) and f_2 up by 10% (or 5%).
- Narrower: The band was narrowed by shifting f_1 up and f_2 down.
- Shift Up: Both f_1 and f_2 were shifted up.
- Shift Down: Both f_1 and f_2 were shifted down.

The results, summarized in Supplementary Table 9, demonstrate that the model's performance is highly stable across all conditions. Even with a ±10% perturbation to the frequency boundaries, the mean AUROC remained within a tight range (0.902

to 0.908), showing no significant degradation compared to the performance with the original, unperturbed cutoffs. This analysis confirms that our framework is not dependent on the exact precision of the knee frequency detection and is robust to small variations in the spectral characteristics of the data.

	10 percent perturbation		5 percent perturbation	
Method	AUROC	Accuracy	AUROC	Accuracy
wider	0.908 [0.891, 0.925]	0.809 [0.733, 0.884]	0.904 [0.890, 0.918]	0.798 [0.744, 0.853]
narrower	0.904 [0.881, 0.927]	0.824 [0.803, 0.845]	0.900 [0.886, 0.914]	0.823 [0.792, 0.854]
shift up	0.905 [0.890, 0.919]	0.823 [0.796, 0.850]	0.899 [0.883, 0.916]	0.807 [0.797, 0.817]
shift down	0.902 [0.898, 0.907]	0.811 [0.794, 0.829]	0.901 [0.884, 0.918]	0.823 [0.797, 0.849]

Supplementary Table 9 Sensitivity analysis of frequency cutoffs on the ABCD sex classification task. The original knee frequencies (f_1, f_2) were perturbed by ±5% and ±10% across four conditions. Performance metrics are reported as mean [95% Confidence Interval] calculated from three random seeds.

Test–Retest Stability of Frequency Band Definitions

We estimated the data-driven knee frequencies (f_1, f_2) for each subject in the HCP resting-state fMRI LR runs (run-1 and run-2) using the same pipeline as in the main analyses. A paired two-sided test found no evidence of run effects (f_1: $p = 1.000$; f_2: $p = 0.9428$), supporting short-interval test–retest stability of the knee frequencies.

Spatial module and temporal module.

	ABCD sex classification	
Module	AUROC ↑	Accuracy ↑
Spatial	0.553 [0.512, 0.594]	0.540 [0.488, 0.590]
Temporal	0.909 [0.897, 0.922]	0.831 [0.797, 0.865]
Spatiotemporal (MBBN)	0.916 [0.903, 0.929]	0.844 [0.826, 0.861]

Supplementary Table 10 Results of spatial and temporal modules of MBBN in the timeseries data extracted by HCP-MMP1 atlas. Performance is reported as mean [95% Confidence Interval]

Table 10 illustrates the performance of individual spatial and temporal modules compared to the full MBBN spatiotemporal architecture on ABCD/UKB sex classification tasks. The results demonstrate that spatial and temporal modules interact synergistically through joint parameter updates during training. When used independently, both modules show diminished performance compared to the integrated spatiotemporal approach (MBBN). The spatial module achieves moderate performance, while the temporal module performs considerably worse on the ABCD dataset.

These findings suggest that the neural dynamics captured in fMRI data require both spatial and temporal processing to maximize predictive power. The superior performance of the integrated MBBN architecture implies that allowing these modules to interact through shared loss functions and simultaneous parameter updates creates representations that better capture the complex spatiotemporal patterns in brain activity. This emphasizes the importance of modeling both spatial and temporal dimensions of neural data rather than treating them as independent components.

Number of parameters and FLOPs

Module	AUROC ↑	Accuracy ↑	FLOPs	Number of Params
Vanilla BERT (FLOPs-matched)	0.886 [0.872, 0.900]	0.807 [0.791, 0.824]	70.489 B	29.894 M
Vanilla BERT (Params-matched)	0.864 [0.851, 0.877]	0.777 [0.759, 0.795]	23.498 B	10.051 M
MBBN	0.916 [0.903, 0.929]	0.844 [0.826, 0.861]	70.528 B	11.141 M

Supplementary Table 11 Performance of vanilla BERT with parameter count or FLOPs matched to MBBN levels. Experiments were conducted on the ABCD dataset, and the task was sex classification.

Table 11 demonstrates a fair comparison between MBBN and vanilla BERT models with matched computational complexity. Two variants of vanilla BERT were created: one matching MBBN's FLOPs (70.5B vs 70.5B) and another matching MBBN's parameter count (10.1M vs 11.1M). Despite having similar computational resources or parameter counts, MBBN significantly outperforms both Vanilla BERT variants in AUROC and accuracy. These results strongly suggest that MBBN's superior performance cannot be attributed simply to increased model capacity or computational complexity. Rather, the architectural innovations in MBBN appear to be responsible for its enhanced effectiveness compared to vanilla BERT baselines.

Comparison with other models

Performance comparison

The tables above present a comparison of various models, atlases, and metrics for different downstream tasks trained from scratch. In these tables, (CC) refers to correlation-based connectivity, and (TD) represents temporal dynamics. Specifically, (CC) denotes Pearson correlation computed on temporal dynamics. Across most tasks, MBBN (highlighted in color) generally outperforms or shows comparable performance to other baseline models.

For instance, in the ABCD depression classification task, the dataset's imbalance in the depression-HC ratio naturally leads to high regular accuracy. However,

Models (data form)	ABCD sex classification, HCP MMP1 atlas	
	AUROC ↑	Accuracy ↑
XGBOOST (CC)	0.785 [0.771, 0.799]	0.709 [0.701, 0.717]
BNT (CC)	0.872 [0.856, 0.889]	0.795 [0.773, 0.818]
BolT (TD)	0.886 [0.881, 0.891]	0.795 [0.790, 0.800]
vanilla BERT (TD)	0.864 [0.851, 0.877]	0.777 [0.759, 0.795]
MBBN (TD)	0.916 [0.903, 0.929]	0.844 [0.826, 0.861]

Supplementary Table 12 Results of models trained from scratch predicting biological phenotypes (ABCD sex). Atlas is HCP MMP 1. CC denotes correlation-based connectivity and TD denotes temporal dynamics.

Models (data form)	UKB sex classification, HCP MMP1 atlas	
	AUROC ↑	Accuracy ↑
XGBOOST (CC)	0.859 [0.851, 0.867]	0.768 [0.752, 0.784]
BNT (CC)	0.950 [0.939, 0.961]	0.864 [0.811, 0.917]
BolT (TD)	0.950 [0.949, 0.952]	0.878 [0.876, 0.880]
vanilla BERT (TD)	0.942 [0.901, 0.984]	0.876 [0.859, 0.894]
MBBN (TD)	0.980 [0.975, 0.986]	0.921 [0.903, 0.938]

Supplementary Table 13 Results of models trained from scratch predicting biological phenotypes (UKB sex). Atlas is HCP MMP 1. CC denotes correlation-based connectivity and TD denotes temporal dynamics.

Models	ABCD sex classification, Schaefer 400 atlas	
	AUROC ↑	Accuracy ↑
XGBOOST (CC)	0.812 [0.800, 0.824]	0.734 [0.726, 0.742]
BNT (CC)	0.920 [0.903, 0.937]	0.839 [0.802, 0.877]
BolT (TD)	0.911 [0.905, 0.917]	0.826 [0.816, 0.837]
vanilla BERT (TD)	0.901 [0.879, 0.924]	0.818 [0.767, 0.869]
MBBN (TD)	0.918 [0.913, 0.923]	0.831 [0.805, 0.857]

Supplementary Table 14 Results of models trained from scratch predicting biological phenotypes (ABCD sex). Atlas is Schaefer 400, 17 Networks. CC denotes correlation-based connectivity and TD denotes temporal dynamics.

Models	UKB sex classification, Schaefer 400 atlas	
	AUROC ↑	Accuracy ↑
XGBOOST (CC)	0.905 [0.891, 0.919]	0.884 [0.880, 0.888]
BNT (CC)	0.982 [0.980, 0.984]	0.934 [0.929, 0.939]
BolT (TD)	0.982 [0.981, 0.983]	0.932 [0.928, 0.935]
vanilla BERT (TD)	0.980 [0.977, 0.983]	0.925 [0.910, 0.940]
MBBN (TD)	0.987 [0.984, 0.990]	0.933 [0.908, 0.959]

Supplementary Table 15 Results of models trained from scratch predicting biological phenotypes (UKB sex). Atlas is Schaefer 400, 17 Networks. CC denotes correlation-based connectivity and TD denotes temporal dynamics.

Models (data form)	ABCD depression classification, HCP MMP1 atlas	
	AUROC ↑	Accuracy ↑
XGBOOST (CC)	0.548 [0.468, 0.627]	0.500 [0.500, 0.500]
BNT (CC)	0.546 [0.500, 0.591]	0.847 [0.830, 0.864]
BolT (TD)	0.588 [0.571, 0.605]	0.848 [0.844, 0.853]
vanilla BERT (TD)	0.546 [0.500, 0.592]	0.818 [0.661, 0.975]
MBBN (TD)	0.614 [0.554, 0.675]	0.610 [0.578, 0.643]

Supplementary Table 16 Results of models trained from scratch predicting ABCD depression, atlas is HCP-MMP1. CC denotes correlation-based connectivity and TD denotes temporal dynamics.

Models (data form)	UKB depression regression, HCP MMP1 atlas	
	MAE ↓	MSE ↓
XGBOOST (CC)	2.417 [2.341, 2.492]	12.342 [10.211, 13.685]
BNT (CC)	2.276 [2.231, 2.321]	13.989 [11.915, 16.063]
BolT (TD)	2.217 [2.151, 2.285]	12.729 [11.730, 14.189]
vanilla BERT (TD)	2.194 [2.076, 2.311]	12.751 [10.377, 15.124]
MBBN (TD)	2.210 [2.085, 2.334]	13.205 [10.296, 16.115]

Supplementary Table 17 Results of models trained from scratch predicting UKB depression, atlas is HCP-MMP1. CC denotes correlation-based connectivity and TD denotes temporal dynamics.

Models (data form)	ABCD depression classification, Schaefer 400 atlas	
	AUROC ↑	Accuracy ↑
XGBOOST (CC)	0.581 [0.545, 0.617]	0.834 [0.814, 0.854]
BNT (CC)	0.579 [0.525, 0.633]	0.842 [0.812, 0.872]
BolT (TD)	0.585 [0.551, 0.620]	0.844 [0.830, 0.858]
vanilla BERT (TD)	0.586 [0.464, 0.708]	0.846 [0.835, 0.857]
MBBN (TD)	0.641 [0.595, 0.687]	0.777 [0.744, 0.810]

Supplementary Table 18 Results of models trained from scratch predicting clinical outcomes (ABCD depression). Atlas is Schaefer 400, 17 Networks. CC denotes correlation-based connectivity, and TD denotes temporal dynamics.

MBBN demonstrates robustness against this imbalance by achieving more balanced performance metrics compared to other models (Table 24, 25, 26, 27).

Tables 24, 25, 26 and 27 present a comparison across models and metrics for various fine-tuning tasks. Here, MBBN is compared under different pretraining strategies:

- MBBN [from scratch]: Trained without pretraining.
- MBBN [low]: Pretrained with masking loss that targets nodes with low communicability.
- MBBN [random]: Pretrained with random masking loss.
- MBBN [high]: Pretrained with masking loss that targets nodes with high communicability.

Models (data form)	UKB depression regression, Schaefer 400 atlas	
	MAE ↓	MSE ↓
XGBOOST (CC)	2.408 [2.403, 2.413]	11.966 [11.801, 12.130]
BNT (CC)	2.329 [2.291, 2.366]	15.179 [6.873, 23.485]
BolT (TD)	2.201 [2.189, 2.212]	13.044 [12.127, 13.960]
vanilla BERT (TD)	2.230 [2.213, 2.247]	13.359 [11.903, 14.815]
MBBN (TD)	2.249 [2.234, 2.264]	14.486 [10.432, 16.540]

Supplementary Table 19 Results of models trained from scratch predicting clinical outcomes (UKB depression). Atlas is Schaefer 400, 17 Networks. CC denotes correlation-based connectivity, and TD denotes temporal dynamics.

Models (data form)	ABCD fluid intelligence regression, HCP MMP1 atlas	
	MAE ↓	MSE ↓
XGBOOST (CC)	0.720 [0.710, 0.729]	0.827 [0.805, 0.849]
BNT (CC)	0.763 [0.710, 0.816]	1.061 [0.810, 1.312]
BolT (TD)	0.728 [0.716, 0.741]	0.845 [0.813, 0.876]
vanilla BERT (TD)	0.699 [0.649, 0.748]	0.788 [0.680, 0.897]
MBBN (TD)	0.694 [0.640, 0.748]	0.774 [0.649, 0.899]

Supplementary Table 20 Results of models trained from scratch predicting ABCD fluid intelligence, atlas is HCPMMP1. CC denotes correlation-based connectivity and TD denotes temporal dynamics.

Models (data form)	UKB fluid intelligence regression, HCP MMP1 atlas	
	MAE ↓	MSE ↓
XGBOOST (CC)	1.609 [1.583, 1.636]	4.078 [3.817, 4.339]
BNT (CC)	1.581 [1.519, 1.644]	3.993 [1.840, 6.146]
BolT (TD)	1.589 [1.583, 1.595]	3.973 [3.955, 3.992]
vanilla BERT (TD)	1.597 [1.550, 1.644]	3.985 [3.666, 4.304]
MBBN (TD)	1.630 [1.611, 1.650]	4.225 [3.845, 4.606]

Supplementary Table 21 Results of models trained from scratch predicting UKB fluid intelligence, atlas is HCPMMP1. CC denotes correlation-based connectivity and TD denotes temporal dynamics.

MBBN [high], highlighted in bold text, consistently outperforms or matches the performance of other baseline models and pretraining strategies across various tasks. This result emphasizes the effectiveness of the high-communicability masking strategy in enhancing model generalization and fine-tuning performance.

FLOPs and number of parameters

The parameter count difference between our proposed MBBN model and the baseline models (Table 28) stems from our architectural design, which processes data through

Models (data form)	ABCD fluid intelligence regression, Schaefer 400 atlas	
	MAE ↓	MSE ↓
XGBOOST (CC)	0.779 [0.665, 0.777]	0.970 [0.938, 1.003]
BNT (CC)	0.781 [0.719, 0.842]	0.845 [0.584, 1.106]
BolT (TD)	0.718 [0.704, 0.731]	0.822 [0.773, 0.871]
vanilla BERT (TD)	0.721 [0.704, 0.731]	0.823 [0.705, 0.941]
MBBN (TD)	0.719 [0.680, 0.757]	0.828 [0.744, 0.913]

Supplementary Table 22 Results of models trained from scratch predicting cognitive outcomes (ABCD fluid intelligence). Atlas is Schaefer 400, 17 Networks. CC denotes correlation-based connectivity, and TD denotes temporal dynamics.

Models (data form)	UKB fluid intelligence regression, Schaefer 400 atlas	
	MAE ↓	MSE ↓
XGBOOST (CC)	1.600 [1.592, 1.607]	4.003 [3.928, 4.079]
BNT (CC)	1.552 [1.495, 1.609]	4.459 [-2.013, 10.931]
BolT (TD)	1.576 [1.559, 1.593]	3.919 [3.879, 3.958]
vanilla BERT (TD)	1.585 [1.526, 1.645]	3.985 [3.666, 4.304]
MBBN (TD)	1.628 [1.474, 1.782]	4.205 [3.348, 5.062]

Supplementary Table 23 Results of models trained from scratch predicting cognitive outcomes (UKB fluid intelligence). Atlas is Schaefer 400, 17 Networks. CC denotes correlation-based connectivity, and TD denotes temporal dynamics.

three separate streams. This design choice was intentional to capture complex patterns through specialized processing pathways. While this results in a higher parameter count, our ablation studies (Suplementary Materials 5) demonstrate that the performance improvements arise primarily from the model's architecture rather than simply from increased capacity.

Interpretability

In the following tables, two regions involved in connectivity (based on HCP-MMP1) are presented, along with whether the connectivity shows higher scores in the disorder group or the healthy control (HC) group. Additionally, corrected p-values and Cohen's d scores were rounded to three decimal places. Due to space limitations, only the top 100 connectivity pairs are displayed.

ABCD ADHD Classification, HCP MMP1 atlas		
Models (data form)	AUROC ↑	Accuracy ↑
XGBOOST (CC)	0.575 [0.559, 0.592]	0.542 [0.529, 0.556]
BNT (CC)	0.620 [0.545, 0.695]	0.593 [0.536, 0.650]
BolT (TD)	0.607 [0.603, 0.612]	0.597 [0.577, 0.614]
Vanilla BERT (TD)	0.606 [0.523, 0.690]	0.594 [0.552, 0.636]
MBBN [from scratch] (TD)	0.633 [0.606, 0.660]	0.593 [0.546, 0.640]
MBBN [random] (TD)	0.603 [0.587, 0.619]	0.589 [0.567, 0.611]
MBBN [low] (TD)	0.615 [0.584, 0.647]	0.602 [0.582, 0.620]
MBBN [high] (TD)	0.645 [0.623, 0.666]	0.601 [0.563, 0.639]

Supplementary Table 24 Finetuning results for ABCD ADHD classification using pre-trained BERT models with different pretraining losses (HCP-MMP1 atlas). CC denotes correlation-based connectivity, TD denotes temporal dynamics, and MBBN represents our proposed model. The [random], [low], and [high] variants indicate different pretraining strategies based on masking node communicability. Values in brackets represent the 95% confidence intervals.

ABIDE ASD Classification, HCP MMP1 atlas		
Models (data form)	AUROC ↑	Accuracy ↑
XGBOOST (CC)	0.573 [0.442, 0.704]	0.546 [0.407, 0.686]
BNT (CC)	0.691 [0.180, 1.202]	0.617 [0.427, 0.806]
BolT (TD)	0.632 [0.616, 0.648]	0.566 [0.557, 0.576]
Vanilla BERT (TD)	0.644 [0.373, 0.915]	0.625 [0.470, 0.780]
MBBN [from scratch] (TD)	0.780 [0.742, 0.818]	0.657 [0.544, 0.769]
MBBN [random] (TD)	0.601 [0.598, 0.604]	0.534 [0.510, 0.559]
MBBN [low] (TD)	0.583 [0.450, 0.715]	0.547 [0.518, 0.576]
MBBN [high] (TD)	0.810 [0.795, 0.825]	0.717 [0.694, 0.740]

Supplementary Table 25 Finetuning results for ABIDE ASD classification using pre-trained BERT models with different pretraining losses (HCP-MMP1 atlas). CC denotes correlation-based connectivity, TD denotes temporal dynamics, and MBBN represents our proposed model. The [random], [low], and [high] variants indicate different pretraining strategies based on masking node communicability. Values in brackets represent the 95% confidence intervals.

ABCD ADHD Classification, Schaefer 400 atlas		
Models (data form)	AUROC ↑	Accuracy ↑
XGBOOST (CC)	0.594 [0.570, 0.618]	0.564 [0.545, 0.583]
BNT (CC)	0.620 [0.566, 0.674]	0.599 [0.546, 0.653]
BolT (TD)	0.599 [0.570, 0.628]	0.589 [0.556, 0.622]
Vanilla BERT (TD)	0.604 [0.552, 0.656]	0.600 [0.577, 0.623]
MBBN [from scratch] (TD)	0.608 [0.560, 0.656]	0.570 [0.535, 0.606]
MBBN [random] (TD)	0.596 [0.553, 0.638]	0.593 [0.494, 0.691]
MBBN [low] (TD)	0.584 [0.483, 0.684]	0.574 [0.471, 0.678]
MBBN [high] (TD)	0.618 [0.560, 0.677]	0.580 [0.474, 0.685]

Supplementary Table 26 Finetuning results on ABCD ADHD classification (Schaefer 400 atlas). CC denotes correlation-based connectivity, TD denotes temporal dynamics, and MBBN represents our proposed model. The [random], [low], and [high] variants indicate different pretraining strategies based on masking node communicability.

ABIDE ASD Classification, Schaefer 400 atlas		
Models (data form)	AUROC ↑	Accuracy ↑
XGBOOST (CC)	0.660 [0.590, 0.730]	0.629 [0.538, 0.720]
BNT (CC)	0.715 [0.579, 0.850]	0.652 [0.416, 0.887]
BolT (TD)	0.608 [0.523, 0.693]	0.578 [0.494, 0.661]
Vanilla BERT (TD)	0.664 [0.213, 1.115]	0.667 [0.487, 0.846]
MBBN [from scratch] (TD)	0.800 [0.595, 1.004]	0.683 [0.407, 0.718]
MBBN [random] (TD)	0.696 [0.538, 0.854]	0.563 [0.294, 0.831]
MBBN [low] (TD)	0.500 [0.346, 0.654]	0.479 [0.300, 0.658]
MBBN [high] (TD)	0.822 [0.765, 0.879]	0.768 [0.731, 0.803]

Supplementary Table 27 Finetuning results on ABIDE ASD classification (Schaefer 400 atlas). CC denotes correlation-based connectivity, TD denotes temporal dynamics, and MBBN represents our proposed model. The [random], [low], and [high] variants indicate different pretraining strategies based on masking node communicability.

Models (data form)	Number of parameters	FLOPs
XGBOOST (CC)	6004	400
BNT (CC)	11,678,810	13,084,233,536
BolT (TD)	20,697,661	50,032,700,640
Vanilla BERT (TD)	22,280,137	69,848,980,256
MBBN (TD)	23,370,075	209,581,818,912

Supplementary Table 28 The table shows the number of parameters and FLOPs of baseline models used in this study and MBBN (our model).

Supplementary Table 29: Top connectivity features in high-frequency range of ABCD ADHD prediction.

Region 1	Region 2	Corrected p-value	Disorder/HC	Cohen's d
Area OP4-PV R	Area 10v R	HC	< 0.001	0.564
Primary Motor Cortex R	Area 10v R	HC	< 0.001	0.561
Primary Motor Cortex L	Area 10v R	HC	< 0.001	0.56
Medial Belt Complex R	Area 10v R	HC	< 0.001	0.559
Area OP1-SII R	Area 10v R	HC	< 0.001	0.559
Area PF Opercular R	Area 10v R	HC	< 0.001	0.558
Area 43 L	Area 10v R	HC	< 0.001	0.557
Area a24 R	Ventral Area 6 L	HC	< 0.001	0.554
Superior Temporal Visual Area R	Area 10v R	HC	< 0.001	0.554
Area Posterior 24 prime R	Area 10v R	HC	< 0.001	0.554
Area 1 R	Area 10v R	HC	< 0.001	0.553
PreSubiculum R	Area 10v R	HC	< 0.001	0.551
Anterior IntraParietal Area R	Area 10v R	HC	< 0.001	0.55
Area PFt R	Area 10v R	HC	< 0.001	0.549
Area posterior 24 L	Ventral Area 6 L	HC	< 0.001	0.548
Ventral Area 6 L	Area 10v R	HC	< 0.001	0.547
Area 2 R	Area 10v R	HC	< 0.001	0.546
Anterior Ventral Insular Area L	Area 10v R	HC	< 0.001	0.545
Area PFcm R	Area 10v R	HC	< 0.001	0.544
Frontal Opercular Area 3 L	Area 10v R	HC	< 0.001	0.543
Ventral IntraParietal Complex L	Area 10v R	HC	< 0.001	0.541
Area PFm Complex R	Area 10v R	HC	< 0.001	0.536
VentroMedial Visual Area 1 R	Area 10v R	HC	< 0.001	0.536
Area 7PC R	Area 10v R	HC	< 0.001	0.536

Continued on next page

Supplementary Table 29 – continued from previous page

Region 1	Region 2	Disorder/HC	Corrected p-value	Cohen's d
Area V3B R	Area 10v R	HC	< 0.001	0.535
Area posterior 24 L	Area 10v R	HC	< 0.001	0.535
RetroInsular Cortex R	Area 10v R	HC	< 0.001	0.535
Area TE1 anterior L	Ventral Area 6 L	HC	< 0.001	0.534
Ventral Area 24d R	Area 10v R	HC	< 0.001	0.534
Area 25 R	Ventral Area 6 L	HC	< 0.001	0.534
Area p32 prime R	Area 10v R	HC	< 0.001	0.534
Insular Granular Complex R	Area 10v R	HC	< 0.001	0.534
Area 43 R	Area 10v R	HC	< 0.001	0.531
Area 1 L	Area 10v R	HC	< 0.001	0.53
Area TA2 L	Area 10v R	HC	< 0.001	0.53
Primary Sensory Cortex R	Area 10v R	HC	< 0.001	0.53
Area PGi R	Area 10v R	HC	< 0.001	0.53
Para-Insular Area L	Area 10v R	HC	< 0.001	0.529
Frontal Eye Fields L	Area 10v R	HC	< 0.001	0.529
Area 46 R	Area 10v R	HC	< 0.001	0.528
Area OP2-3-VS R	Area 10v R	HC	< 0.001	0.527
Area 55b R	Area 10v R	HC	< 0.001	0.527
Area dorsal 32 R	Ventral Area 6 L	HC	< 0.001	0.527
VentroMedial Visual Area 2 R	Area 10v R	HC	< 0.001	0.526
Dorsal Transitional Visual Area R	Area 10v R	HC	< 0.001	0.526
Area dorsal 32 L	Area 11l R	HC	< 0.001	0.525
Frontal Opercular Area 2 R	Area 10v R	HC	< 0.001	0.525
Area OP2-3-VS L	Area 10v R	HC	< 0.001	0.524
Area 10r R	Ventral Area 6 L	HC	< 0.001	0.524
Area 10v L	Ventral Area 6 L	HC	< 0.001	0.524

Continued on next page

Supplementary Table 29 – continued from previous page

Region 1	Region 2	Disorder/HC	Corrected p-value	Cohen's d
Frontal Opercular Area 4 L	Ventral Area 6 L	HC	< 0.001	0.524
Rostral Area 6 L	Area 10v R	HC	< 0.001	0.523
Frontal Opercular Area 1 R	Area 10v R	HC	< 0.001	0.523
Area PF Complex R	Area 10v R	HC	< 0.001	0.522
Area STSv anterior L	Ventral Area 6 L	HC	< 0.001	0.521
Area s32 L	Ventral Area 6 L	HC	< 0.001	0.52
Area TemporoParietoOccipital Junction 1 L	Area 10v R	HC	< 0.001	0.52
Frontal Opercular Area 4 L	Area 10v R	HC	< 0.001	0.52
Area 13l R	Area 10v R	HC	< 0.001	0.52
Area 3a R	Area 10v R	HC	< 0.001	0.519
Area PGi R	Primary Motor Cortex R	HC	< 0.001	0.518
Dorsal Area 24d R	Area 10v R	HC	< 0.001	0.518
Area dorsal 32 L	Ventral Area 6 L	HC	< 0.001	0.518
Area Posterior 24 prime L	Area 10v R	HC	< 0.001	0.518
Medial Belt Complex R	Primary Motor Cortex R	HC	< 0.001	0.518
Area posterior 24 R	Ventral Area 6 L	HC	< 0.001	0.517
Area PF Opercular L	Area 10v R	HC	< 0.001	0.517
Area IntraParietal 2 R	Area 10v R	HC	< 0.001	0.517
Area 47m R	Area 10v R	HC	< 0.001	0.517
Area 25 L	Ventral Area 6 L	HC	< 0.001	0.517
RetroSplenial Complex R	Area 10v R	HC	< 0.001	0.516
Parieto-Occipital Sulcus Area 1 L	Area 10v R	HC	< 0.001	0.516
Sixth Visual Area R	Area 10v R	HC	< 0.001	0.516
Area Frontal Opercular 5 L	Area 11l R	HC	< 0.001	0.516
Orbital Frontal Complex L	Ventral Area 6 L	HC	< 0.001	0.515

Continued on next page

Supplementary Table 29 – continued from previous page

Region 1	Region 2	Disorder/HC	Corrected p-value	Cohen's d
ParaHippocampal Area 3 R	Area 10v R	HC	< 0.001	0.515
Area p32 R	Ventral Area 6 L	HC	< 0.001	0.514
Area 10d L	Ventral Area 6 L	HC	< 0.001	0.514
Area a24 L	Ventral Area 6 L	HC	< 0.001	0.514
Area 43 L	Ventral Area 6 L	HC	< 0.001	0.514
Frontal Opercular Area 3 R	Area 10v R	HC	< 0.001	0.513
Supplementary and Cingulate Eye Field R	Area 10v R	HC	< 0.001	0.513
Dorsal area 6 L	Area 10v R	HC	< 0.001	0.513
Area PFt L	Area 10v R	HC	< 0.001	0.513
Area V6A R	Area 10v R	HC	< 0.001	0.513
Insular Granular Complex L	Area 10v R	HC	< 0.001	0.513
Area PFcm L	Area 10v R	HC	< 0.001	0.513
Anterior 24 prime R	Area 10v R	HC	< 0.001	0.513
Anterior Ventral Insular Area L	Ventral Area 6 L	HC	< 0.001	0.513
ParaHippocampal Area 1 R	Area 10v R	HC	< 0.001	0.512
PeriSylvian Language Area L	Area 10v R	HC	< 0.001	0.511
RetroSplenial Complex R	Area Posterior Insular 1 L	HC	< 0.001	0.511
Area 9-46d R	Area 10v R	HC	< 0.001	0.511
Area posterior 24 L	Area 11l R	HC	< 0.001	0.511
VentroMedial Visual Area 3 R	Area 10v R	HC	< 0.001	0.511
Area 47s L	Area 11l R	HC	< 0.001	0.511
Parieto-Occipital Sulcus Area 2 R	Area 10v R	HC	< 0.001	0.51
Area TG dorsal L	Area 10v R	HC	< 0.001	0.51
Area 2 L	Area 10v R	HC	< 0.001	0.51

Continued on next page

Supplementary Table 29 – continued from previous page

Region 1	Region 2	Disorder/HC	Corrected p-value	Cohen's d
Supplementary and Cingulate Eye Field L	Area 10v R	HC	< 0.001	0.51

Supplementary Table 30: Top connectivity features in low frequency range of ABCD ADHD prediction

Region 1	Region 2	Disorder/HC	Corrected p-value	Cohen's d
PeriSylvian Language Area L	Area OP2-3-VS R	HC	< 0.001	0.441
Dorsal Area 24d L	Area OP2-3-VS R	HC	< 0.001	0.441
Area 31pd L	Auditory 4 Complex R	HC	< 0.001	0.44
Area STSv posterior R	Auditory 4 Complex R	HC	< 0.001	0.44
Area STSv posterior R	Area OP2-3-VS R	HC	< 0.001	0.439
Dorsal area 6 R	Area OP2-3-VS R	HC	< 0.001	0.435
Area TE1 Middle L	Frontal Opercular Area 2 R	HC	< 0.001	0.435
Area TE1 anterior R	Auditory 4 Complex R	HC	< 0.001	0.434
Area STSd anterior L	Area OP2-3-VS R	HC	< 0.001	0.433
Auditory 5 Complex R	Area OP2-3-VS R	HC	< 0.001	0.431
Superior Temporal Visual Area L	Insular Granular Complex L	HC	< 0.001	0.43
RetroInsular Cortex L	Area OP2-3-VS R	HC	< 0.001	0.429
Area TE1 anterior R	Area OP2-3-VS R	HC	< 0.001	0.429
Area STSd posterior L	Area OP2-3-VS R	HC	< 0.001	0.428
Area 23d L	Frontal Opercular Area 2 R	HC	< 0.001	0.424
Area TG dorsal L	Insular Granular Complex L	HC	< 0.001	0.423
Sixth Visual Area R	Area OP2-3-VS R	HC	< 0.001	0.423
PeriSylvian Language Area L	Insular Granular Complex L	HC	< 0.001	0.423
PeriSylvian Language Area L	Frontal Opercular Area 2 R	HC	< 0.001	0.422
Entorhinal Cortex R	Area OP2-3-VS R	HC	< 0.001	0.422
PeriSylvian Language Area L	Auditory 4 Complex R	HC	< 0.001	0.422
Area STSv anterior L	Area OP2-3-VS R	HC	< 0.001	0.421
Area s32 L	Frontal Opercular Area 2 R	HC	< 0.001	0.421
Area s32 L	Area OP2-3-VS R	HC	< 0.001	0.421

Continued on next page

Supplementary Table 30 – continued from previous page

Region 1	Region 2	Disorder/HC	Corrected p-value	Cohen's d
Area TE1 Middle L	Area OP2-3-VS R	HC	< 0.001	0.42
Area 8Av L	Insular Granular Complex L	HC	< 0.001	0.42
Area STSd anterior R	Area OP2-3-VS R	HC	< 0.001	0.418
Area PHT R	Area OP2-3-VS R	HC	< 0.001	0.417
VentroMedial Visual Area 3 L	Insular Granular Complex L	HC	< 0.001	0.417
Area V6A L	Area OP2-3-VS R	HC	< 0.001	0.417
Area 47l (47 lateral) R	Area OP2-3-VS R	HC	< 0.001	0.416
Dorsal area 6 L	Area OP2-3-VS R	HC	< 0.001	0.415
Area 8Ad L	Insular Granular Complex L	HC	< 0.001	0.414
Area 8C L	Insular Granular Complex L	HC	< 0.001	0.414
Area TE1 anterior L	Auditory 4 Complex R	HC	< 0.001	0.413
Area V6A L	Frontal Opercular Area 2 R	HC	< 0.001	0.413
Area TG dorsal L	Auditory 4 Complex R	HC	< 0.001	0.413
Auditory 5 Complex L	Area OP2-3-VS R	HC	< 0.001	0.412
Area 10d R	Frontal Opercular Area 2 R	HC	< 0.001	0.412
Area 47s R	Frontal Opercular Area 2 R	HC	< 0.001	0.411
Area TE1 Middle L	Auditory 4 Complex R	HC	< 0.001	0.411
Area TE1 posterior L	Insular Granular Complex L	HC	< 0.001	0.411
Hippocampus R	Auditory 4 Complex R	HC	< 0.001	0.411
Area TE1 Middle L	Insular Granular Complex L	HC	< 0.001	0.41
Area STSd posterior L	Auditory 4 Complex R	HC	< 0.001	0.41
Primary Auditory Cortex R	Auditory 4 Complex R	HC	< 0.001	0.41
Dorsal Area 24d L	Auditory 4 Complex R	HC	< 0.001	0.41
Area dorsal 32 L	Area OP2-3-VS R	HC	< 0.001	0.41
Area TE1 posterior R	Area OP2-3-VS R	HC	< 0.001	0.409
Dorsal area 6 R	Insular Granular Complex L	HC	< 0.001	0.409

Continued on next page

Supplementary Table 30 – continued from previous page

Region 1	Region 2	Disorder/HC	Corrected p-value	Cohen's d
Primary Motor Cortex R	Auditory 4 Complex R	HC	< 0.001	0.409
Area TemporoParietoOccipital Junction 1 R	Frontal Opercular Area 2 R	HC	< 0.001	0.409
Area TE1 anterior R	Frontal Opercular Area 2 R	HC	< 0.001	0.409
Area TE1 Middle R	Area OP2-3-VS R	HC	< 0.001	0.408
Dorsal area 6 R	Frontal Opercular Area 2 R	HC	< 0.001	0.408
Lateral Belt Complex R	Auditory 4 Complex R	HC	< 0.001	0.408
Area 31pd L	Insular Granular Complex L	HC	< 0.001	0.408
Superior 6-8 Transitional Area L	Insular Granular Complex L	HC	< 0.001	0.408
Lateral Belt Complex R	Area OP2-3-VS R	HC	< 0.001	0.407
Area TG dorsal R	Auditory 4 Complex R	HC	< 0.001	0.407
Area 31pd L	Area OP2-3-VS R	HC	< 0.001	0.407
Area dorsal 32 L	Frontal Opercular Area 2 R	HC	< 0.001	0.407
Area 10d R	Area OP2-3-VS R	HC	< 0.001	0.407
Area STSd anterior L	Insular Granular Complex L	HC	< 0.001	0.406
Area 8B Lateral L	Insular Granular Complex L	HC	< 0.001	0.405
Area 31pd R	Auditory 4 Complex R	HC	< 0.001	0.405
Area 8Ad L	Area OP2-3-VS R	HC	< 0.001	0.405
Pirform Cortex L	Area OP2-3-VS R	HC	< 0.001	0.405
Frontal Opercular Area 1 R	Area OP2-3-VS R	HC	< 0.001	0.405
Area TemporoParietoOccipital Junction 1 R	Auditory 4 Complex R	HC	< 0.001	0.405
Dorsal area 6 R	Medial Belt Complex R	HC	< 0.001	0.405
Area 8B Lateral L	Area OP2-3-VS R	HC	< 0.001	0.404
Area PGs L	Area OP2-3-VS R	HC	< 0.001	0.404

Continued on next page

Supplementary Table 30 – continued from previous page

Region 1	Region 2	Disorder/HC	Corrected p-value	Cohen's d
Area TemporoParietoOccipital Junction 1 R	Area OP2-3-VS R	HC	< 0.001	0.404
Area 6mp R	Auditory 4 Complex R	HC	< 0.001	0.403
Area IFJa R	Auditory 4 Complex R	HC	< 0.001	0.403
Area 45 L	Insular Granular Complex L	HC	< 0.001	0.403
Area 31pd R	Insular Granular Complex L	HC	< 0.001	0.403
Ventral IntraParietal Complex R	Area OP2-3-VS R	HC	< 0.001	0.403
Auditory 5 Complex L	Insular Granular Complex L	HC	< 0.001	0.403
Auditory 4 Complex L	Area OP2-3-VS R	HC	< 0.001	0.403
Area PFcm R	Auditory 4 Complex R	HC	< 0.001	0.403
Area 6 anterior L	Area OP2-3-VS R	HC	< 0.001	0.403
Area dorsal 32 L	Insular Granular Complex L	HC	< 0.001	0.403
Area 10d L	Area OP2-3-VS R	HC	< 0.001	0.403
Area 9 Posterior L	Insular Granular Complex L	HC	< 0.001	0.402
Middle Temporal Area R	Area OP2-3-VS R	HC	< 0.001	0.402
Area 8Ad L	Frontal Opercular Area 2 R	HC	< 0.001	0.402
Area TE1 Middle L	Posterior Insular Area 2 R	HC	< 0.001	0.401
Area STSd anterior R	Auditory 4 Complex R	HC	< 0.001	0.401
Area TG dorsal R	Frontal Opercular Area 2 R	HC	< 0.001	0.401
Area TE1 posterior R	Frontal Opercular Area 2 R	HC	< 0.001	0.401
Area 7m R	Auditory 4 Complex R	HC	< 0.001	0.401
Entorhinal Cortex R	Auditory 4 Complex R	HC	< 0.001	0.401
Area 47l (47 lateral) L	Area OP2-3-VS R	HC	< 0.001	0.4
PreCuneus Visual Area L	Insular Granular Complex L	HC	< 0.001	0.4
Area STGa R	Area OP2-3-VS R	HC	< 0.001	0.4
Superior Temporal Visual Area L	Area OP2-3-VS R	HC	< 0.001	0.4

Continued on next page

Supplementary Table 30 – continued from previous page

Region 1	Region 2	Disorder/HC	Corrected p-value	Cohen's d
Area TG dorsal L	Area OP2-3-VS R	HC	< 0.001	0.4
Medial Belt Complex R	Area OP2-3-VS R	HC	< 0.001	0.399

Supplementary Table 31: Top connectivity features in ultralow frequency range of ABCD ADHD prediction

Region 1	Region 2	Disorder/HC	Corrected p-value	Cohen's d
Medial Belt Complex R	Area PGp R	HC	< 0.001	0.373
Area dorsal 32 R	ParaHippocampal Area 1 R	ADHD	< 0.001	0.372
Area FST R	ParaHippocampal Area 1 R	ADHD	< 0.001	0.368
Area dorsal 32 R	Area OP2-3-VS R	ADHD	< 0.001	0.367
Medial Belt Complex R	Seventh Visual Area R	HC	< 0.001	0.363
Area PGi L	ParaHippocampal Area 1 R	ADHD	< 0.001	0.361
ParaHippocampal Area 3 R	Area TF R	HC	0.001	0.346
Area 47s R	Area PGp R	HC	0.001	0.344
Area 8BM R	Area OP2-3-VS R	ADHD	0.001	0.343
Area FST R	Area OP2-3-VS R	ADHD	0.001	0.343
Area 11l L	Area PGp R	HC	0.001	0.343
Area 8Av R	ParaHippocampal Area 1 R	ADHD	0.001	0.343
Area 11l L	Anterior IntraParietal Area L	HC	0.001	0.342
Area 47l (47 lateral) L	Anterior IntraParietal Area L	HC	0.001	0.341
Area PGi L	Area OP2-3-VS R	ADHD	0.001	0.339
Area 8BM R	ParaHippocampal Area 1 R	ADHD	0.001	0.339
Area FST R	Area Posterior Insular 1 L	ADHD	0.001	0.338
Area TemporoParietoOccipital Junction 2 L	ParaHippocampal Area 1 R	ADHD	0.001	0.337
Area 47s R	Anterior IntraParietal Area L	HC	0.001	0.336
Area OP4-PV R	Area PGp R	HC	0.001	0.336
Pirform Cortex R	Area PGp R	HC	0.001	0.336
Dorsal Transitional Visual Area R	ParaHippocampal Area 1 R	ADHD	0.001	0.335
Area dorsal 23 a+b R	ParaHippocampal Area 1 R	ADHD	0.001	0.334

Continued on next page

Supplementary Table 31 – continued from previous page

Region 1	Region 2	Corrected p-value	Disorder/HC	Cohen's d
Fourth Visual Area L	Area OP2-3-VS R	ADHD	0.001	0.334
Area STSv posterior R	Area 47s R	ADHD	0.001	0.333
Area Posterior Insular 1 L	Medial Superior Temporal Area L	HC	0.001	0.33
Area 47s R	Seventh Visual Area R	HC	0.001	0.33
Area STSv posterior R	Polar 10p R	ADHD	0.001	0.33
Area 11l L	Seventh Visual Area R	HC	0.001	0.33
Area OP4-PV R	Area TF R	HC	0.001	0.329
Medial Belt Complex R	Sixth Visual Area L	HC	0.001	0.328
Area STSv posterior R	Anterior Agranular Insula Complex R	ADHD	0.002	0.328
Area STSv posterior R	ParaHippocampal Area 1 R	ADHD	0.002	0.328
Area 5L R	ParaHippocampal Area 1 R	ADHD	0.002	0.328
Area IFSa R	Area OP2-3-VS R	ADHD	0.002	0.327
Medial Belt Complex R	Medial Superior Temporal Area L	HC	0.002	0.326
Area STGa R	Area TF R	HC	0.002	0.325
Area 11l L	Area 7PC L	HC	0.002	0.325
Area V3A L	Area OP2-3-VS R	ADHD	0.002	0.324
Area posterior 9-46v R	Area OP2-3-VS R	ADHD	0.002	0.324
Area anterior 9-46v R	Area OP2-3-VS R	ADHD	0.002	0.324
Fourth Visual Area R	Area 10v L	ADHD	0.002	0.323
Medial Belt Complex R	Anterior IntraParietal Area L	HC	0.002	0.323
Area STSv posterior R	Orbital Frontal Complex L	ADHD	0.002	0.323
Area PFm Complex L	Area OP2-3-VS R	ADHD	0.002	0.322
Area STSv posterior R	Area OP2-3-VS R	ADHD	0.002	0.322
Medial Area 7A R	ParaHippocampal Area 1 R	ADHD	0.002	0.321
Medial Belt Complex R	Area V3A L	HC	0.002	0.32
Area OP4-PV R	Medial Superior Temporal Area L	HC	0.002	0.32

Continued on next page

Supplementary Table 31 – continued from previous page

Region 1	Region 2	Corrected p-value	Disorder/HC	Cohen's d
Area STSv posterior R	Area 10v L	ADHD	0.002	0.32
Area 11l L	Fourth Visual Area L	HC	0.002	0.32
Area STSd posterior L	ParaHippocampal Area 1 R	ADHD	0.002	0.319
Area dorsal 32 R	Area Posterior 24 prime R	ADHD	0.002	0.319
Area dorsal 32 R	Hippocampus R	ADHD	0.002	0.319
Area Posterior Insular 1 L	Anterior IntraParietal Area L	HC	0.002	0.318
Area PFm Complex L	ParaHippocampal Area 1 R	ADHD	0.002	0.318
Third Visual Area R	Area OP2-3-VS R	ADHD	0.002	0.318
Area FST L	ParaHippocampal Area 1 R	ADHD	0.002	0.317
Area FST R	Pirform Cortex L	ADHD	0.002	0.317
Area FST R	Area p32 L	ADHD	0.002	0.317
Anterior IntraParietal Area L	ParaHippocampal Area 1 R	ADHD	0.002	0.316
Area 11l L	Ventral IntraParietal Complex L	HC	0.002	0.316
Area STSv posterior R	Area 47l (47 lateral) R	ADHD	0.002	0.316
Area STSd posterior L	Area OP2-3-VS R	ADHD	0.002	0.316
Area ventral 23 a+b R	ParaHippocampal Area 1 R	ADHD	0.002	0.315
Area STSv posterior R	Pirform Cortex L	ADHD	0.002	0.315
Medial Belt Complex R	Second Visual Area L	HC	0.002	0.315
Area Posterior Insular 1 L	Area PGp R	HC	0.002	0.315
Area 47l (47 lateral) L	Medial Superior Temporal Area L	HC	0.002	0.315
Area 8Av R	Area OP2-3-VS R	ADHD	0.002	0.314
Pirform Cortex R	Seventh Visual Area R	HC	0.002	0.314
Sixth Visual Area L	ParaHippocampal Area 1 R	ADHD	0.002	0.314
Area 43 L	Area OP2-3-VS R	ADHD	0.002	0.313
Area STSv posterior R	Area p32 L	ADHD	0.002	0.313
Area TG dorsal R	Area PGp R	HC	0.002	0.313

Continued on next page

Supplementary Table 31 – continued from previous page

Region 1	Region 2	Corrected p-value	Disorder/HC	Cohen's d
Medial Superior Temporal Area L	ParaHippocampal Area 1 R	ADHD	0.003	0.312
Area dorsal 32 R	Anterior Agranular Insula Complex R	ADHD	0.002	0.312
Area p32 R	ParaHippocampal Area 1 R	ADHD	0.003	0.312
Area PGi L	Hippocampus R	ADHD	0.003	0.312
Area STGa L	Anterior IntraParietal Area L	HC	0.002	0.312
Medial Area 7A R	Area OP2-3-VS R	ADHD	0.003	0.311
Dorsal area 6 R	Medial Superior Temporal Area L	HC	0.003	0.311
Area TE1 Middle R	ParaHippocampal Area 1 R	ADHD	0.003	0.311
Area p32 L	Medial Superior Temporal Area L	HC	0.003	0.31
IntraParietal Sulcus Area 1 R	ParaHippocampal Area 1 R	ADHD	0.003	0.31
Fusiform Face Complex R	ParaHippocampal Area 1 R	ADHD	0.003	0.309
Area 11l L	Medial Superior Temporal Area L	HC	0.003	0.309
Area 44 R	ParaHippocampal Area 1 R	ADHD	0.003	0.309
Area 23c R	ParaHippocampal Area 1 R	ADHD	0.003	0.309
Medial Belt Complex R	Area V6A R	HC	0.003	0.309
Parieto-Occipital Sulcus Area 1 R	Anterior IntraParietal Area L	HC	0.003	0.308
Medial Belt Complex R	Ventral IntraParietal Complex L	HC	0.003	0.308
Dorsal area 6 R	Seventh Visual Area R	HC	0.003	0.308
Area 47l (47 lateral) L	Area PGp R	HC	0.003	0.308
Area STSv posterior R	Area anterior 10p L	ADHD	0.003	0.307
Ventral Area 6 L	Medial Superior Temporal Area L	HC	0.003	0.306
Area TemporoParietoOccipital Junction 1 R	ParaHippocampal Area 1 R	ADHD	0.003	0.306
Pirform Cortex L	Area PGp R	HC	0.003	0.306
Area STSv posterior R	Area 47s L	ADHD	0.003	0.305
Parieto-Occipital Sulcus Area 2 L	ParaHippocampal Area 1 R	ADHD	0.003	0.305

Supplementary Table 32: Top connectivity features in high frequency range of ABIDE ASD prediction

Region 1	Region 2	Disorder/HC	Corrected p-value	Cohen's d
Area 2 L	Area 11l R	HC	0.002	2.232
Area PFcm L	Area 11l R	HC	0.003	2.191
RetroInsular Cortex L	Area 11l R	HC	0.003	2.162
Ventral Area 24d L	Area 11l R	HC	0.003	2.162
Area IntraParietal 2 L	Area 11l R	HC	0.003	2.149
Area Posterior 24 prime R	Area 11l R	HC	0.003	2.147
Area Posterior 24 prime L	Area 11l R	HC	0.003	2.147
Ventral Area 24d R	Area 11l R	HC	0.003	2.145
Area 1 L	Area 11l R	HC	0.003	2.141
Area 44 R	Area 11l R	HC	0.003	2.14
Primary Auditory Cortex L	Area 11l R	HC	0.003	2.127
Area dorsal 32 R	Area 11l R	HC	0.004	2.11
Area Frontal Opercular 5 L	Area 11l R	HC	0.004	2.106
Area anterior 32 prime L	Area 11l R	HC	0.004	2.104
Parieto-Occipital Sulcus Area 2 R	Area 11l R	HC	0.004	2.101
Anterior IntraParietal Area L	Area 11l R	HC	0.004	2.101
Anterior 24 prime R	Area 11l R	HC	0.004	2.099
Area 43 R	Area 11l R	HC	0.004	2.097
Orbital Frontal Complex R	Area 11l R	HC	0.004	2.091
Area OP4-PV R	Area 11l R	HC	0.004	2.09
Area 23d L	Area 11l R	HC	0.004	2.085
Frontal Opercular Area 3 L	Area 11l R	HC	0.004	2.083
Area PGi R	Area 11l R	HC	0.004	2.082
Dorsal Area 24d L	Area 11l R	HC	0.004	2.082

Continued on next page

Supplementary Table 32 – continued from previous page

Region 1	Region 2	Corrected p-value	Disorder/HC	Cohen's d
Primary Sensory Cortex L	Area 11l R	HC	0.004	2.08
Second Visual Area R	Sixth Visual Area L	ASD	0.004	2.075
Area PFt L	Area 11l R	HC	0.004	2.075
Area 7PC L	Area 11l R	HC	0.004	2.073
Area anterior 32 prime R	Area 11l R	HC	0.004	2.07
Area 33 prime L	Area 11l R	HC	0.004	2.067
Area OP2-3-VS L	Area 11l R	HC	0.004	2.066
Area posterior 10p R	Area 11l R	HC	0.004	2.064
Dorsal Transitional Visual Area R	Area 11l R	HC	0.004	2.064
Area ventral 23 a+b L	Area 11l R	HC	0.004	2.055
Area 23d R	Area 11l R	HC	0.004	2.054
Area 7m R	Area 11l R	HC	0.004	2.053
Area 10v R	Area 11l R	HC	0.004	2.05
Lateral Area 7A L	Area 11l R	HC	0.004	2.05
Area IntraParietal 1 L	Area 11l R	HC	0.004	2.049
Area Frontal Opercular 5 R	Area 11l R	HC	0.004	2.049
Anterior 24 prime L	Area 11l R	HC	0.004	2.049
Area posterior 9-46v L	Area 11l R	HC	0.004	2.046
Area p32 prime R	Area 11l R	HC	0.004	2.046
Area ventral 23 a+b R	Area 11l R	HC	0.004	2.045
Area posterior 24 L	Area 11l R	HC	0.004	2.045
Area dorsal 32 L	Area 11l R	HC	0.004	2.044
PeriSylvian Language Area L	Area 11l R	HC	0.004	2.044
Auditory 4 Complex R	Area 11l R	HC	0.004	2.044
Lateral Belt Complex R	Area 11l R	HC	0.004	2.043
Area 8BM R	Area 11l R	HC	0.004	2.043

Continued on next page

Supplementary Table 32 – continued from previous page

Region 1	Region 2	Corrected p-value	Disorder/HC	Cohen's d
Area PGs L	Area 11l R	HC	0.004	2.041
Area 10d R	Area 11l R	HC	0.004	2.04
Fourth Visual Area L	Sixth Visual Area L	ASD	0.005	2.039
Rostral Area 6 R	Area 11l R	HC	0.005	2.038
Area 33 prime R	Area 11l R	HC	0.005	2.035
Insular Granular Complex L	Area 11l R	HC	0.005	2.034
Ventral Area 6 R	Area 11l R	HC	0.005	2.034
Seventh Visual Area R	Sixth Visual Area L	ASD	0.005	2.032
Area dorsal 23 a+b L	Area 11l R	HC	0.005	2.027
Orbital Frontal Complex L	Area 11l R	HC	0.005	2.027
Area TE2 anterior L	Area 11l R	HC	0.005	2.027
Medial Belt Complex R	Area 11l R	HC	0.005	2.026
Area 45 R	Area 11l R	HC	0.005	2.025
Medial Area 7P R	Area 11l R	HC	0.005	2.023
Ventral Area 6 L	Area 11l R	HC	0.005	2.021
Dorsal Area 24d R	Area 11l R	HC	0.005	2.02
ProStriate Area L	Area 11l R	HC	0.005	2.02
Area OP1-SII L	Area 11l R	HC	0.005	2.02
Medial IntraParietal Area L	Area 11l R	HC	0.005	2.019
Area anterior 47r R	Area 11l R	HC	0.005	2.018
Superior 6-8 Transitional Area R	Area 11l R	HC	0.005	2.015
Area 8Ad R	Area 11l R	HC	0.005	2.012
Area IFSp L	Area 11l R	HC	0.005	2.012
Superior Temporal Visual Area L	Area 11l R	HC	0.005	2.011
Ventral IntraParietal Complex L	Area 11l R	HC	0.005	2.011
Area p32 prime L	Area 11l R	HC	0.005	2.01

Continued on next page

Supplementary Table 32 – continued from previous page

Region 1	Region 2	Corrected p-value	Disorder/HC	Cohen's d
PreSubiculum R	Sixth Visual Area L	ASD	0.005	2.007
Area 8B Lateral R	Area 11l R	HC	0.005	2.005
Area V6A L	Area 11l R	HC	0.005	2.004
Area IFJp L	Area 11l R	HC	0.005	2.004
Area Lateral IntraParietal ventral L	Area 11l R	HC	0.005	2.004
Area 8Av R	Area 11l R	HC	0.005	2.004
Area IFJa L	Area 11l R	HC	0.005	2.004
Parieto-Occipital Sulcus Area 2 L	Area 11l R	HC	0.005	2.003
Frontal Opercular Area 4 L	Area 11l R	HC	0.005	2.003
Area PF Opercular L	Area 11l R	HC	0.005	2.001
Area posterior 24 R	Area 11l R	HC	0.005	2.001
Area PFm Complex R	Area 11l R	HC	0.005	2.0
Area 5L R	Area 11l R	HC	0.005	2.0
Area 10v L	Area 11l R	HC	0.005	1.998
Area 5m ventral L	Area 11l R	HC	0.005	1.998
Anterior Ventral Insular Area L	Area 11l R	HC	0.005	1.996
Area posterior 9-46v R	Area 11l R	HC	0.005	1.994
Area PGp L	Area 11l R	HC	0.005	1.992
Frontal Opercular Area 2 L	Area 11l R	HC	0.005	1.991
Area PFm Complex L	Area 11l R	HC	0.005	1.99
Area Lateral IntraParietal dorsal L	Area 11l R	HC	0.005	1.988
Parieto-Occipital Sulcus Area 1 L	Area 11l R	HC	0.005	1.988
Area 31p ventral L	Area 11l R	HC	0.005	1.987
Area V6A R	Area 11l R	HC	0.005	1.987

Supplementary Table 33: Top connectivity features in low frequency range of ABIDE ASD prediction

Region 1	Region 2	Corrected p-value	Disorder/HC	Cohen's d
Frontal Opercular Area 3 L	Polar 10p R	HC	0.003	2.128
Frontal Opercular Area 2 L	Polar 10p R	HC	0.003	2.123
Area V3A R	Polar 10p R	HC	0.003	2.117
Area posterior 47r L	Polar 10p R	HC	0.004	2.109
IntraParietal Sulcus Area 1 L	Polar 10p R	HC	0.004	2.101
Area OP2-3-VS R	Polar 10p R	HC	0.004	2.1
Frontal Opercular Area 3 R	Polar 10p R	HC	0.004	2.096
Frontal Opercular Area 2 R	Polar 10p R	HC	0.004	2.095
Area 44 L	Polar 10p R	HC	0.004	2.093
Para-Insular Area R	Polar 10p R	HC	0.004	2.093
Eighth Visual Area R	Polar 10p R	HC	0.004	2.091
Ventral IntraParietal Complex R	Polar 10p R	HC	0.004	2.09
Area IFSp L	Polar 10p R	HC	0.004	2.087
Area anterior 9-46v L	Polar 10p R	HC	0.004	2.083
Area STGa R	Polar 10p R	HC	0.004	2.082
Medial IntraParietal Area L	Polar 10p R	HC	0.004	2.078
ParaHippocampal Area 1 R	Polar 10p R	HC	0.004	2.075
Rostral Area 6 R	Polar 10p R	HC	0.004	2.074
Area V3B L	Polar 10p R	HC	0.004	2.074
Pirform Cortex L	Polar 10p R	HC	0.004	2.074
Anterior 24 prime L	Polar 10p R	HC	0.004	2.073
Para-Insular Area L	Polar 10p R	HC	0.004	2.072
Area TF R	Polar 10p R	HC	0.004	2.071
Primary Auditory Cortex R	Polar 10p R	HC	0.004	2.071

Continued on next page

Supplementary Table 33 – continued from previous page

Region 1	Region 2	Disorder/HC	Corrected p-value	Cohen's d
Area PFt R	Polar 10p R	HC	0.004	2.069
Area V4t L	Polar 10p R	HC	0.004	2.069
Area OP1-SII R	Polar 10p R	HC	0.004	2.069
Area V3B R	Polar 10p R	HC	0.004	2.069
Pirform Cortex R	Polar 10p R	HC	0.004	2.068
Area IntraParietal 0 L	Polar 10p R	HC	0.004	2.067
Area Frontal Opercular 5 L	Polar 10p R	HC	0.004	2.064
Frontal Opercular Area 1 R	Polar 10p R	HC	0.004	2.064
Area 55b L	Polar 10p R	HC	0.004	2.064
Area OP2-3-VS L	Polar 10p R	HC	0.004	2.064
ProStriate Area R	Polar 10p R	HC	0.004	2.063
Area STSd posterior L	Polar 10p R	HC	0.004	2.062
Area IntraParietal 0 R	Polar 10p R	HC	0.004	2.062
Area 8B Lateral L	Polar 10p R	HC	0.004	2.061
Area STSd anterior L	Polar 10p R	HC	0.004	2.061
Lateral Belt Complex R	Polar 10p R	HC	0.004	2.061
Lateral Area 7P L	Polar 10p R	HC	0.004	2.061
IntraParietal Sulcus Area 1 R	Polar 10p R	HC	0.004	2.06
Area OP1-SII L	Polar 10p R	HC	0.004	2.06
Area 2 R	Polar 10p R	HC	0.004	2.059
Area IFJa R	Polar 10p R	HC	0.004	2.059
Ventral IntraParietal Complex L	Polar 10p R	HC	0.004	2.058
Area Posterior Insular 1 R	Polar 10p R	HC	0.004	2.058
Area 7PC R	Polar 10p R	HC	0.004	2.058
Area IFJp R	Polar 10p R	HC	0.004	2.057
Insular Granular Complex R	Polar 10p R	HC	0.004	2.057

Continued on next page

Supplementary Table 33 – continued from previous page

Region 1	Region 2	Disorder/HC	Corrected p-value	Cohen's d
Area IFSa L	Polar 10p R	HC	0.004	2.057
Dorsal Transitional Visual Area L	Polar 10p R	HC	0.004	2.056
Posterior Insular Area 2 R	Polar 10p R	HC	0.004	2.055
Area 33 prime R	Polar 10p R	HC	0.004	2.055
Frontal Opercular Area 4 L	Polar 10p R	HC	0.004	2.054
Area Lateral IntraParietal ventral R	Polar 10p R	HC	0.004	2.053
Area 8BM L	Polar 10p R	HC	0.004	2.053
Ventral Visual Complex R	Polar 10p R	HC	0.004	2.053
Middle Insular Area L	Polar 10p R	HC	0.004	2.053
Area 9 Middle R	Polar 10p R	HC	0.004	2.052
Insular Granular Complex L	Polar 10p R	HC	0.004	2.051
Sixth Visual Area R	Polar 10p R	HC	0.004	2.051
Lateral Area 7A R	Polar 10p R	HC	0.004	2.051
Ventral Visual Complex L	Polar 10p R	HC	0.004	2.05
Area TG dorsal R	Polar 10p R	HC	0.004	2.05
Frontal Opercular Area 4 R	Polar 10p R	HC	0.004	2.05
Anterior IntraParietal Area R	Polar 10p R	HC	0.004	2.05
Hippocampus R	Polar 10p R	HC	0.004	2.05
PreSubiculum L	Polar 10p R	HC	0.004	2.05
Area anterior 10p L	Polar 10p R	HC	0.004	2.048
Area IFJp L	Polar 10p R	HC	0.004	2.047
Seventh Visual Area R	Polar 10p R	HC	0.004	2.047
VentroMedial Visual Area 2 R	Polar 10p R	HC	0.004	2.046
Eighth Visual Area L	Polar 10p R	HC	0.004	2.045
Area TA2 R	Polar 10p R	HC	0.004	2.044
Fourth Visual Area R	Polar 10p R	HC	0.004	2.044

Continued on next page

Supplementary Table 33 – continued from previous page

Region 1	Region 2	Disorder/HC	Corrected p-value	Cohen's d
Area PFcm R	Polar 10p R	HC	0.004	2.044
Area PFm Complex R	Polar 10p R	HC	0.004	2.044
Area PFm Complex L	Polar 10p R	HC	0.004	2.044
Area TE1 anterior R	Polar 10p R	HC	0.004	2.043
Posterior Insular Area 2 L	Polar 10p R	HC	0.004	2.043
Area 8Ad L	Polar 10p R	HC	0.004	2.043
Area dorsal 32 R	Polar 10p R	HC	0.004	2.042
Area V4t R	Polar 10p R	HC	0.004	2.042
Frontal Eye Fields L	Polar 10p R	HC	0.004	2.041
Posterior InferoTemporal complex L	Polar 10p R	HC	0.004	2.041
Area Lateral IntraParietal dorsal R	Polar 10p R	HC	0.004	2.041
Area PF Complex R	Polar 10p R	HC	0.004	2.04
Area 9 Middle L	Polar 10p R	HC	0.004	2.04
Area STSd anterior R	Polar 10p R	HC	0.004	2.04
Area 43 L	Polar 10p R	HC	0.004	2.04
VentroMedial Visual Area 3 L	Polar 10p R	HC	0.004	2.04
Area 8Av L	Polar 10p R	HC	0.004	2.039
Area 6mp L	Polar 10p R	HC	0.004	2.039
Area TE2 anterior R	Polar 10p R	HC	0.004	2.039
Area 1 L	Polar 10p R	HC	0.004	2.037
Area V6A L	Polar 10p R	HC	0.005	2.037
ParaBelt Complex R	Polar 10p R	HC	0.005	2.037
Area 44 R	Polar 10p R	HC	0.005	2.036
VentroMedial Visual Area 3 R	Polar 10p R	HC	0.005	2.036

Supplementary Table 34: Top connectivity features in ultralow frequency range of ABIDE ASD prediction

Region 1	Region 2	Disorder/HC	Corrected p-value	Cohen's d
Area PHT L	Area TemporoParietoOccipital Junction 3 L	ASD	< 0.001	5.637
Area 9 Posterior R	Area TemporoParietoOccipital Junction 3 L	ASD	< 0.001	5.334
Area 8Ad R	Area TemporoParietoOccipital Junction 3 L	ASD	< 0.001	3.99
Area 8B Lateral L	Area TemporoParietoOccipital Junction 3 L	ASD	< 0.001	3.835
Orbital Frontal Complex L	Area TF R	ASD	< 0.001	3.767
Superior Frontal Language Area L	Area TemporoParietoOccipital Junction 3 L	ASD	< 0.001	3.566
Superior 6-8 Transitional Area L	Area TemporoParietoOccipital Junction 3 L	ASD	< 0.001	3.566
Area TE2 posterior L	Area Posterior 24 prime R	ASD	< 0.001	3.549
RetroInsular Cortex R	Area anterior 32 prime L	ASD	< 0.001	3.54
Frontal Opercular Area 2 L	Ventral IntraParietal Complex R	ASD	< 0.001	3.499
Fourth Visual Area L	Area anterior 32 prime L	ASD	< 0.001	3.494
Lateral Area 7A R	Area TemporoParietoOccipital Junction 3 L	ASD	< 0.001	3.38
Frontal Eye Fields L	Area TemporoParietoOccipital Junction 3 L	ASD	< 0.001	3.355
Area V3B R	Area 9-46d L	ASD	0.002	3.339
Medial Area 7P L	Area anterior 32 prime L	ASD	0.002	3.328
Fourth Visual Area L	Area PFm Complex L	ASD	0.002	3.295

Continued on next page

Supplementary Table 34 – continued from previous page

Region 1	Region 2	Corrected p-value	Disorder/HC	Cohen's d
Sixth Visual Area R	Area TemporoParietoOccipital Junction 3 L	ASD	< 0.001	3.252
Area posterior 9-46v R	Area anterior 32 prime L	ASD	< 0.001	3.201
Second Visual Area R	Area TemporoParietoOccipital Junction 3 L	ASD	< 0.001	3.176
Area 8BM R	Area TemporoParietoOccipital Junction 3 L	ASD	< 0.001	3.173
Area STSd posterior L	Area TemporoParietoOccipital Junction 3 L	ASD	< 0.001	3.169
Second Visual Area R	Area 23d R	ASD	< 0.001	3.143
IntraParietal Sulcus Area 1 R	Area 23d R	ASD	< 0.001	3.128
Lateral Area 7P L	RetroInsular Cortex L	ASD	< 0.001	3.08
Lateral Area 7P R	RetroInsular Cortex L	ASD	< 0.001	3.076
Medial Area 7P L	Primary Sensory Cortex R	ASD	< 0.001	3.064
Second Visual Area L	Area anterior 32 prime L	ASD	< 0.001	3.024
Second Visual Area L	Area PFm Complex L	ASD	< 0.001	3.012
Area posterior 9-46v L	RetroInsular Cortex L	ASD	< 0.001	3.009
Area TE2 posterior L	Area 5m R	ASD	< 0.001	2.983
Area 5m L	Area anterior 32 prime L	ASD	< 0.001	2.966
Area PH L	Area anterior 32 prime L	ASD	< 0.001	2.963
Area 2 R	Area anterior 32 prime L	ASD	< 0.001	2.962
Area V3B R	Area anterior 32 prime L	ASD	< 0.001	2.95
Area 9 Middle L	Area TemporoParietoOccipital Junction 3 L	ASD	< 0.001	2.928
Area 31a R	Area TemporoParietoOccipital Junction 3 L	ASD	< 0.001	2.921

Continued on next page

Supplementary Table 34 – continued from previous page

Region 1	Region 2	Corrected p-value	Disorder/HC	Cohen's d
Perirhinal Ectorhinal Cortex R	Area TemporoParietoOccipital Junction 3 L	ASD	< 0.001	2.919
Area 6m anterior R	Area TemporoParietoOccipital Junction 3 L	ASD	< 0.001	2.913
Area PH L	Area PFm Complex L	ASD	< 0.001	2.878
Ventral Area 6 R	Area TemporoParietoOccipital Junction 3 L	ASD	< 0.001	2.867
Parieto-Occipital Sulcus Area 1 L	Medial Superior Temporal Area R	ASD	< 0.001	2.859
Ventral Visual Complex R	Area anterior 32 prime L	ASD	< 0.001	2.859
Area anterior 32 prime R	Area 23d R	ASD	< 0.001	2.844
Area TemporoParietoOccipital Junction 3 R	Area anterior 32 prime L	ASD	< 0.001	2.838
ParaHippocampal Area 2 R	Area anterior 32 prime L	ASD	< 0.001	2.821
Area 9-46d R	Area TemporoParietoOccipital Junction 3 L	ASD	< 0.001	2.82
Area V3B R	Area Posterior 24 prime R	ASD	< 0.001	2.801
Area PHT L	Area dorsal 23 a+b L	ASD	< 0.001	2.801
Area 47m L	Area Lateral Occipital 1 L	ASD	< 0.001	2.796
Ventral IntraParietal Complex L	Medial Superior Temporal Area L	ASD	< 0.001	2.796
Area IntraParietal 2 R	Area anterior 32 prime L	ASD	< 0.001	2.789
Area STSv posterior L	Area 23d R	ASD	0.007	2.781
VentroMedial Visual Area 1 L	Primary Sensory Cortex R	ASD	< 0.001	2.775
Area posterior 24 L	Area TemporoParietoOccipital Junction 3 L	ASD	< 0.001	2.774
Medial Area 7A R	Primary Sensory Cortex R	ASD	< 0.001	2.767
Primary Motor Cortex R	Area anterior 32 prime L	ASD	0.001	2.762

Continued on next page

Supplementary Table 34 – continued from previous page

Region 1	Region 2	Corrected p-value	Disorder/HC	Cohen's d
Medial Area 7A L	Primary Sensory Cortex R	ASD	0.001	2.753
Medial Area 7P L	Area PFm Complex L	ASD	< 0.001	2.751
Dorsal Transitional Visual Area L	Medial Superior Temporal Area R	ASD	0.001	2.744
Area Lateral Occipital 2 R	RetroInsular Cortex L	ASD	0.001	2.736
Area Lateral Occipital 2 R	Medial Superior Temporal Area R	ASD	0.001	2.734
Area dorsal 32 L	Area TemporoParietoOccipital Junction 3 L	ASD	0.001	2.723
Area TG Ventral L	RetroInsular Cortex L	ASD	0.001	2.711
Area Lateral IntraParietal dorsal R	Area anterior 32 prime L	ASD	0.001	2.696
Primary Visual Cortex R	Area 23d R	ASD	0.001	2.688
Area p32 R	Medial Superior Temporal Area R	ASD	0.005	2.685
Area STSv posterior L	Area PFm Complex L	ASD	0.005	2.683
Area PFt R	Area TemporoParietoOccipital Junction 3 L	ASD	0.001	2.681
Area 31a R	Area PFm Complex L	ASD	0.001	2.672
Area TemporoParietoOccipital Junction 3 R	Area PFm Complex L	ASD	0.001	2.669
Dorsal Transitional Visual Area R	Area TemporoParietoOccipital Junction 3 L	ASD	0.001	2.658
Frontal Opercular Area 3 L	Area 8Av L	ASD	0.001	2.645
Anterior IntraParietal Area R	Area anterior 32 prime L	ASD	0.001	2.627
Dorsal Transitional Visual Area R	Area PFm Complex L	ASD	0.001	2.62
Posterior Insular Area 2 R	Area anterior 32 prime L	ASD	0.001	2.618
Primary Visual Cortex L	Area 23d R	ASD	0.001	2.615
Medial Area 7A L	Area anterior 32 prime L	ASD	0.001	2.608
Primary Visual Cortex L	Area PFm Complex L	ASD	0.001	2.606

Continued on next page

Supplementary Table 34 – continued from previous page

Region 1	Region 2	Corrected p-value	Disorder/HC	Cohen's d
Area 8Av L	Primary Sensory Cortex R	ASD	0.001	2.599
Area TE2 posterior L	RetroInsular Cortex L	ASD	0.001	2.583
Area Lateral Occipital 2 R	Area dorsal 23 a+b L	ASD	0.001	2.58
RetroInsular Cortex R	Area PFm Complex L	ASD	0.001	2.578
Area 1 R	Area TemporoParietoOccipital Junction 3 L	ASD	0.001	2.576
Primary Sensory Cortex R	Area TemporoParietoOccipital Junction 3 L	ASD	0.001	2.572
Area STSv anterior L	Area 6 anterior L	ASD	0.001	2.57
Area V3B R	Medial Belt Complex L	ASD	0.001	2.568
Seventh Visual Area L	Medial Superior Temporal Area L	ASD	0.003	2.565
Area V3A R	Ventral IntraParietal Complex R	ASD	0.001	2.565
Area STGa L	Area 23d R	ASD	0.001	2.558
Area 2 L	Ventral IntraParietal Complex R	ASD	0.001	2.55
Para-Insular Area R	Area 23d R	ASD	0.001	2.548
Inferior 6-8 Transitional Area L	RetroInsular Cortex L	ASD	0.001	2.548
Seventh Visual Area L	Area PFm Complex L	ASD	0.001	2.546
Dorsal area 6 R	Area 9-46d L	ASD	0.001	2.541
Area 8BM L	Area TemporoParietoOccipital Junction 3 L	ASD	0.001	2.536
Area TE2 posterior L	Area Posterior 24 prime L	ASD	0.001	2.535
Area 52 L	Area TemporoParietoOccipital Junction 3 L	ASD	0.001	2.533
Area dorsal 32 R	Area TemporoParietoOccipital Junction 3 L	ASD	0.001	2.532
Ventral Area 6 R	Area 23d R	ASD	0.001	2.53

Continued on next page

Supplementary Table 34 – continued from previous page

Region 1	Region 2	Corrected p-value	Disorder/HC	Cohen's d
Area 9 Posterior L	Area TemporoParietoOccipital Junction 3 L	ASD	0.001	2.53

References

[1] Taghia, J., Cai, W., Ryali, S., Kochalka, J., Nicholas, J., Chen, T., Menon, V.: Uncovering hidden brain state dynamics that regulate performance and decision-making during cognition. Nature communications **9**(1), 2505 (2018)

[2] He, B.J., Zempel, J.M., Snyder, A.Z., Raichle, M.E.: The temporal structures and functional significance of scale-free brain activity. Neuron **66**(3), 353–369 (2010)

[3] Sendi, M.S., Fu, Z., Harnett, N.G., Rooij, S.J., Vergara, V., Pizzagalli, D.A., Daskalakis, N.P., House, S.L., Beaudoin, F.L., An, X., et al.: Brain dynamics reflecting an intra-network brain state are associated with increased post-traumatic stress symptoms in the early aftermath of trauma. Nature Mental Health, 1–14 (2025)

[4] Breakspear, M.: Dynamic models of large-scale brain activity. Nature neuroscience **20**(3), 340–352 (2017)

[5] Kan, X., Dai, W., Cui, H., Zhang, Z., Guo, Y., Yang, C.: Brain network transformer. Advances in Neural Information Processing Systems **35**, 25586–25599 (2022)

[6] Bedel, H.A., Sivgin, I., Dalmaz, O., Dar, S.U., Çukur, T.: Bolt: Fused window transformers for fmri time series analysis. Medical image analysis **88**, 102841 (2023)

[7] Wang, C., Subramaniam, V., Yaari, A.U., Kreiman, G., Katz, B., Cases, I., Barbu, A.: Brainbert: Self-supervised representation learning for intracranial recordings. arXiv preprint arXiv:2302.14367 (2023)

[8] Ortega Caro, J., Oliveira Fonseca, A.H., Averill, C., Rizvi, S.A., Rosati, M., Cross, J.L., Mittal, P., Zappala, E., Levine, D., Dhodapkar, R.M., et al.: Brainlm: A foundation model for brain activity recordings. bioRxiv, 2023–09 (2023)

[9] Stanley, H.E., Meakin, P.: Multifractal phenomena in physics and chemistry. Nature **335**(6189), 405–409 (1988)

[10] Falconer, K.: Fractal Geometry: Mathematical Foundations and Applications, 3rd edn. John Wiley & Sons, Chichester, UK (2013)

[11] Salat, H., Murcio, R., Arcaute, E.: Multifractal methodology. Physica A: Statistical Mechanics and its Applications **473**, 467–487 (2017)

[12] Guidolin, D., Tortorella, C., De Caro, R., Agnati, L.F.: In: Di Ieva, A. (ed.) A Self-Similarity Logic May Shape the Organization of the Nervous System, pp. 203–225. Springer, Cham (2024). https://doi.org/10.1007/978-3-031-47606-8_10 . https://doi.org/10.1007/978-3-031-47606-8_10

[13] Ciuciu, P., Abry, P., Rabrait, C., Wendt, H.: Log wavelet leaders cumulant based multifractal analysis of evi fmri time series: evidence of scaling in ongoing and evoked brain activity. IEEE Journal of Selected Topics in Signal Processing **2**(6), 929–943 (2008)

[14] Smit, D.J., Geus, E.J., Nieuwenhuijzen, M.E., Beijsterveldt, C.E., Baal, G.C.M., Mansvelder, H.D., Boomsma, D.I., Linkenkaer-Hansen, K.: Scale-free modulation of resting-state neuronal oscillations reflects prolonged brain maturation in humans. Journal of Neuroscience **31**(37), 13128–13136 (2011)

[15] Tagliazucchi, E., Wegner, F., Morzelewski, A., Brodbeck, V., Jahnke, K., Laufs, H.: Breakdown of long-range temporal dependence in default mode and attention networks during deep sleep. Proceedings of the National Academy of Sciences **110**(38), 15419–15424 (2013)

[16] Tolkunov, D., Rubin, D., Mujica-Parodi, L.R.: Power spectrum scale invariance quantifies limbic dysregulation in trait anxious adults using fmri: adapting methods optimized for characterizing autonomic dysregulation to neural dynamic time series. Neuroimage **50**(1), 72–80 (2010)

[17] Maxim, V., Şendur, L., Fadili, J., Suckling, J., Gould, R., Howard, R., Bullmore, E.: Fractional gaussian noise, functional mri and alzheimer's disease. Neuroimage **25**(1), 141–158 (2005)

[18] Achard, S., Salvador, R., Whitcher, B., Suckling, J., Bullmore, E.: A resilient, low-frequency, small-world human brain functional network with highly connected association cortical hubs. Journal of Neuroscience **26**(1), 63–72 (2006)

[19] Sasai, S., Koike, T., Sugawara, S.K., Hamano, Y.H., Sumiya, M., Okazaki, S., Takahashi, H.K., Taga, G., Sadato, N.: Frequency-specific task modulation of human brain functional networks: A fast fmri study. NeuroImage **224**, 117375 (2021)

[20] Hagler Jr, D.J., Hatton, S., Cornejo, M.D., Makowski, C., Fair, D.A., Dick, A.S., Sutherland, M.T., Casey, B., Barch, D.M., Harms, M.P., *et al.*: Image processing and analysis methods for the adolescent brain cognitive development study. Neuroimage **202**, 116091 (2019)

[21] Marek, S., Tervo-Clemmens, B., Calabro, F.J., Montez, D.F., Kay, B.P., Hatoum, A.S., Donohue, M.R., Foran, W., Miller, R.L., Hendrickson, T.J., *et al.*: Reproducible brain-wide association studies require thousands of individuals. Nature **603**(7902), 654–660 (2022)

[22] Ingalhalikar, M., Shinde, S., Karmarkar, A., Rajan, A., Rangaprakash, D., Deshpande, G.: Functional connectivity-based prediction of autism on site harmonized abide dataset. IEEE transactions on biomedical engineering **68**(12), 3628–3637 (2021)

[23] Esteban, O., Markiewicz, C.J., Blair, R.W., Moodie, C.A., Isik, A.I., Erramuzpe, A., Kent, J.D., Goncalves, M., DuPre, E., Snyder, M., *et al.*: fmriprep: a robust preprocessing pipeline for functional mri. Nature methods **16**(1), 111–116 (2019)

[24] Behzadi, Y., Restom, K., Liau, J., Liu, T.T.: A component based noise correction method (compcor) for bold and perfusion based fmri. Neuroimage **37**(1), 90–101 (2007)

[25] Glasser, M.F., Coalson, T.S., Robinson, E.C., Hacker, C.D., Harwell, J., Yacoub, E., Ugurbil, K., Andersson, J., Beckmann, C.F., Jenkinson, M., *et al.*: A multi-modal parcellation of human cerebral cortex. Nature **536**(7615), 171–178 (2016)

[26] Schaefer, A., Kong, R., Gordon, E.M., Laumann, T.O., Zuo, X.-N., Holmes, A.J., Eickhoff, S.B., Yeo, B.T.: Local-global parcellation of the human cerebral cortex from intrinsic functional connectivity mri. Cerebral cortex **28**(9), 3095–3114 (2018)

[27] Cordova, M.M., Antovich, D.M., Ryabinin, P., Neighbor, C., Mooney, M.A., Dieckmann, N.F., Miranda-Dominguez, O., Nagel, B.J., Fair, D.A., Nigg, J.T.: Attention-deficit/hyperactivity disorder: restricted phenotypes prevalence, comorbidity, and polygenic risk sensitivity in the abcd baseline cohort. Journal of the American Academy of Child & Adolescent Psychiatry **61**(10), 1273–1284 (2022)

[28] Martin, C.H., Peng, T., Mahoney, M.W.: Predicting trends in the quality of state-of-the-art neural networks without access to training or testing data. Nature Communications **12**(1), 4122 (2021)

[29] Zuo, X.-N., Di Martino, A., Kelly, C., Shehzad, Z.E., Gee, D.G., Klein, D.F., Castellanos, F.X., Biswal, B.B., Milham, M.P.: The oscillating brain: complex and reliable. Neuroimage **49**(2), 1432–1445 (2010)

[30] Coifman, R.R., Wickerhauser, M.V.: Entropy-based algorithms for best basis selection. IEEE Transactions on information theory **38**(2), 713–718 (1992)

[31] Shim, W.H., Baek, K., Kim, J.K., Chae, Y., Suh, J.-Y., Rosen, B.R., Jeong, J., Kim, Y.R.: Frequency distribution of causal connectivity in rat sensorimotor network: resting-state fmri analyses. Journal of Neurophysiology **109**(1), 238–248 (2013)

[32] Devlin, J., Chang, M.-W., Lee, K., Toutanova, K.: Bert: Pre-training of deep bidirectional transformers for language understanding. arXiv preprint arXiv:1810.04805 (2018)

[33] Baevski, A., Zhou, Y., Mohamed, A., Auli, M.: wav2vec 2.0: A framework for self-supervised learning of speech representations. Advances in neural information processing systems **33**, 12449–12460 (2020)

[34] Mohanty, R., Sethares, W.A., Nair, V.A., Prabhakaran, V.: Rethinking measures of functional connectivity via feature extraction. Scientific reports **10**(1), 1298 (2020)

[35] Vaswani, A.: Attention is all you need. Advances in Neural Information Processing Systems (2017)

[36] Selvaraju, R.R., Cogswell, M., Das, A., Vedantam, R., Parikh, D., Batra, D.: Grad-cam: Visual explanations from deep networks via gradient-based localization. In: Proceedings of the IEEE International Conference on Computer Vision, pp. 618–626 (2017)

[37] Cohen, J.: Statistical Power Analysis for the Behavioral Sciences, 2nd edn. routledge, New York, NY (2013)

[38] Chen, T., He, T., Benesty, M., Khotilovich, V., Tang, Y., Cho, H., Chen, K., Mitchell, R., Cano, I., Zhou, T., *et al.*: Xgboost: extreme gradient boosting. R package version 0.4-2 **1**(4), 1–4 (2015)

[39] Kucewicz, M.T., Cimbalnik, J., Garcia-Salinas, J.S., Brazdil, M., Worrell, G.A.: High frequency oscillations in human memory and cognition: a neurophysiological substrate of engrams? Brain **147**(9), 2966–2982 (2024)

[40] Costa, G.N., Schaum, M., Duarte, J.V., Martins, R., Duarte, I.C., Castelhano, J., Wibral, M., Castelo-Branco, M.: Distinct oscillatory patterns differentiate between segregation and integration processes in perceptual grouping. Human Brain Mapping **45**(12), 26779 (2024)

[41] Estrada, E., Hatano, N.: Communicability in complex networks. Physical Review E **77**(3), 036111 (2008)

[42] Estrada, E., Hatano, N., Benzi, M.: The physics of communicability in complex networks. Physics reports **514**(3), 89–119 (2012)

[43] Bullmore, E., Long, C., Suckling, J., Fadili, J., Calvert, G., Zelaya, F., Carpenter, T.A., Brammer, M.: Colored noise and computational inference in neurophysiological (fmri) time series analysis: resampling methods in time and wavelet domains. Human brain mapping **12**(2), 61–78 (2001)

[44] Fox, M.D., Raichle, M.E.: Spontaneous fluctuations in brain activity observed with functional magnetic resonance imaging. Nature reviews neuroscience **8**(9), 700–711 (2007)

[45] He, B.J.: Scale-free properties of the functional magnetic resonance imaging signal during rest and task. Journal of Neuroscience **31**(39), 13786–13795 (2011)

[46] Mandelbrot, B.B., Van Ness, J.W.: Fractional brownian motions, fractional noises

and applications. SIAM review **10**(4), 422–437 (1968)

[47] Eke, A., Herman, P., Kocsis, L., Kozak, L.: Fractal characterization of complexity in temporal physiological signals. Physiological measurement **23**(1), 1 (2002)

[48] Radulescu, A.R., Rubin, D., Strey, H.H., Mujica-Parodi, L.R.: Power spectrum scale invariance identifies prefrontal dysregulation in paranoid schizophrenia. Human brain mapping **33**(7), 1582–1593 (2012)

[49] Cha, J., DeDora, D., Nedic, S., Ide, J., Greenberg, T., Hajcak, G., Mujica-Parodi, L.R.: Clinically anxious individuals show disrupted feedback between inferior frontal gyrus and prefrontal-limbic control circuit. Journal of Neuroscience **36**(17), 4708–4718 (2016)

[50] Tu, W., Liao, Q., Zhou, S., Peng, X., Ma, C., Liu, Z., Liu, X., Cai, Z., He, K.: Rare: Robust masked graph autoencoder. IEEE Transactions on Knowledge and Data Engineering (2023)

[51] Liu, C., Wang, Y., Zhan, Y., Ma, X., Tao, D., Wu, J., Hu, W.: Where to mask: Structure-guided masking for graph masked autoencoders. arXiv preprint arXiv:2404.15806 (2024)

[52] Harush, U., Barzel, B.: Dynamic patterns of information flow in complex networks. Nature communications **8**(1), 2181 (2017)

[53] Burgess, P.W., Wu, H.: Rostral prefrontal cortex (brodmann area 10). Principles of frontal lobe function, 524–544 (2013)

[54] Roth, R.M., Saykin, A.J.: Executive dysfunction in attention-deficit/hyperactivity disorder: cognitive and neuroimaging findings. Psychiatric Clinics **27**(1), 83–96 (2004)

[55] Baker, C.M., Burks, J.D., Briggs, R.G., Conner, A.K., Glenn, C.A., Robbins, J.M., Sheets, J.R., Sali, G., McCoy, T.M., Battiste, J.D., *et al.*: A connectomic atlas of the human cerebrum—chapter 5: The insula and opercular cortex. Operative Neurosurgery **15**(suppl_1), 175–244 (2018)

[56] Peterson, D., Reddy, V., Hamel, R.: Neuroanatomy, auditory pathway. StatPearls. StatPearls Publishing Copyright (2023)

[57] Ito, T., Tiede, M., Ostry, D.J.: Somatosensory function in speech perception. Proceedings of the National Academy of Sciences **106**(4), 1245–1248 (2009)

[58] Conant, D., Bouchard, K.E., Chang, E.F.: Speech map in the human ventral sensory-motor cortex. Current opinion in neurobiology **24**, 63–67 (2014)

[59] LaFlamme, E.M., Waguespack, H.F., Forcelli, P.A., Malkova, L.: The parahippocampal cortex and its functional connection with the hippocampus are

critical for nonnavigational spatial memory in macaques. Cerebral Cortex **31**(4), 2251–2267 (2021)

[60] Bohbot, V.D., Kalina, M., Stepankova, K., Spackova, N., Petrides, M., Nadel, L.: Spatial memory deficits in patients with lesions to the right hippocampus and to the right parahippocampal cortex. Neuropsychologia **36**(11), 1217–1238 (1998)

[61] Skodzik, T., Holling, H., Pedersen, A.: Long-term memory performance in adult adhd: A meta-analysis. Journal of attention disorders **21**(4), 267–283 (2017)

[62] Steinberg, E.A., Drabick, D.A.: A developmental psychopathology perspective on adhd and comorbid conditions: The role of emotion regulation. Child Psychiatry & Human Development **46**, 951–966 (2015)

[63] Bunford, N., Evans, S.W., Wymbs, F.: Adhd and emotion dysregulation among children and adolescents. Clinical child and family psychology review **18**, 185–217 (2015)

[64] Rolls, E.T., Cheng, W., Feng, J.: The orbitofrontal cortex: reward, emotion and depression. Brain communications **2**(2), 196 (2020)

[65] Case-Smith, J., Weaver, L.L., Fristad, M.A.: A systematic review of sensory processing interventions for children with autism spectrum disorders. Autism **19**(2), 133–148 (2015)

[66] Cibralic, S., Kohlhoff, J., Wallace, N., McMahon, C., Eapen, V.: A systematic review of emotion regulation in children with autism spectrum disorder. Research in Autism Spectrum Disorders **68**, 101422 (2019)

[67] Velikonja, T., Fett, A.-K., Velthorst, E.: Patterns of nonsocial and social cognitive functioning in adults with autism spectrum disorder: A systematic review and meta-analysis. JAMA psychiatry **76**(2), 135–151 (2019)

[68] Baker, C.M., Burks, J.D., Briggs, R.G., Conner, A.K., Glenn, C.A., Morgan, J.P., Stafford, J., Sali, G., McCoy, T.M., Battiste, J.D., *et al.*: A connectomic atlas of the human cerebrum—chapter 2: The lateral frontal lobe. Operative Neurosurgery **15**(suppl_1), 10–74 (2018)

[69] Law, C.-K., Kolling, N., Chan, C.C., Chau, B.K.: Frontopolar cortex represents complex features and decision value during choice between environments. Cell reports **42**(6) (2023)

[70] Ferrucci, L., Ceccarelli, F., Londei, F., Arena, G., Elyasizad, L., Nougaret, S., Genovesio, A.: Reward monitoring in the frontopolar cortex of macaques. Scientific Reports **15**(1), 1–14 (2025)

[71] Polónyiová, K., Kruyt, J., Ostatníková, D.: To the roots of theory of mind deficits

in autism spectrum disorder: a narrative review. Review Journal of Autism and Developmental Disorders, 1–5 (2024)

[72] De Benedictis, A., Duffau, H., Paradiso, B., Grandi, E., Balbi, S., Granieri, E., Colarusso, E., Chioffi, F., Marras, C.E., Sarubbo, S.: Anatomo-functional study of the temporo-parieto-occipital region: dissection, tractographic and brain mapping evidence from a neurosurgical perspective. Journal of anatomy **225**(2), 132–151 (2014)

[73] Seghatol-Eslami, V.C., Maximo, J.O., Ammons, C.J., Libero, L.E., Kana, R.K.: Hyperconnectivity of social brain networks in autism during action-intention judgment. Neuropsychologia **137**, 107303 (2020)

[74] Supekar, K., Uddin, L.Q., Khouzam, A., Phillips, J., Gaillard, W.D., Kenworthy, L.E., Yerys, B.E., Vaidya, C.J., Menon, V.: Brain hyperconnectivity in children with autism and its links to social deficits. Cell reports **5**(3), 738–747 (2013)

[75] Chita-Tegmark, M.: Social attention in asd: A review and meta-analysis of eye-tracking studies. Research in developmental disabilities **48**, 79–93 (2016)

[76] Grinsztajn, L., Oyallon, E., Varoquaux, G.: Why do tree-based models still outperform deep learning on typical tabular data? Advances in neural information processing systems **35**, 507–520 (2022)

[77] Kajimura, S., Margulies, D., Smallwood, J.: Frequency-specific brain network architecture in resting-state fmri. Scientific Reports **13**(1), 2964 (2023)
