# Supplementary material for: Learning brain dynamics across distinct scaling regimes reveals psychiatric signatures
Source: Commun Biol. 2026 May 8;9:963. doi: 10.1038/s42003-026-10011-7 (PMC13369815; doi:10.1038/s42003-026-10011-7)
Supplement: Supplementary file 2 — Description of Additional Supplementary Files [file 42003_2026_10011_MOESM2_ESM.docx]

**Description of Additional Supplementary Files**

**File name:** Supplementary Data

**Description:** Raw performance metrics for drawing figures and fill in the tables.
