# Supplementary material for: Learning brain dynamics across distinct scaling regimes reveals psychiatric signatures
Source: Commun Biol. 2026 May 8;9:963. doi: 10.1038/s42003-026-10011-7 (PMC13369815; doi:10.1038/s42003-026-10011-7)
Supplement: Supplementary file 4 — Reporting Summary [file 42003_2026_10011_MOESM4_ESM.pdf]

Corresponding author(s): COMMSBIO-25-5843C

Last updated by author(s): Mar 13, 2026

Reporting Summary

Nature Portfolio wishes to improve the reproducibility of the work that we publish. This form provides structure for consistency and transparency in reporting. For further information on Nature Portfolio policies, see our Editorial Policies and the Editorial Policy Checklist.

Statistics

For all statistical analyses, confirm that the following items are present in the figure legend, table legend, main text, or Methods section.

n/a	Confirmed	
☐	☒	The exact sample size (n) for each experimental group/condition, given as a discrete number and unit of measurement
☐	☒	A statement on whether measurements were taken from distinct samples or whether the same sample was measured repeatedly
☐	☒	The statistical test(s) used AND whether they are one- or two-sided<br>*Only common tests should be described solely by name; describe more complex techniques in the Methods section.*
☐	☒	A description of all covariates tested
☐	☒	A description of any assumptions or corrections, such as tests of normality and adjustment for multiple comparisons
☐	☒	A full description of the statistical parameters including central tendency (e.g. means) or other basic estimates (e.g. regression coefficient) AND variation (e.g. standard deviation) or associated estimates of uncertainty (e.g. confidence intervals)
☐	☒	For null hypothesis testing, the test statistic (e.g. F, t, r) with confidence intervals, effect sizes, degrees of freedom and P value noted<br>*Give P values as exact values whenever suitable.*
☒	☐	For Bayesian analysis, information on the choice of priors and Markov chain Monte Carlo settings
☒	☐	For hierarchical and complex designs, identification of the appropriate level for tests and full reporting of outcomes
☐	☒	Estimates of effect sizes (e.g. Cohen's d, Pearson's r), indicating how they were calculated

Our web collection on statistics for biologists contains articles on many of the points above.

Software and code

Policy information about availability of computer code

Data collection	The UKB, ABCD, and ABIDE datasets are publicly available to researchers
Data analysis	We used our own code (github repository : https://github.com/Transconnectome/MBBN).

For manuscripts utilizing custom algorithms or software that are central to the research but not yet described in published literature, software must be made available to editors and reviewers. We strongly encourage code deposition in a community repository (e.g. GitHub). See the Nature Portfolio guidelines for submitting code & software for further information.

Data

Policy information about availability of data

All manuscripts must include a data availability statement. This statement should provide the following information, where applicable:

- Accession codes, unique identifiers, or web links for publicly available datasets
- A description of any restrictions on data availability
- For clinical datasets or third party data, please ensure that the statement adheres to our policy

The UK Biobank (UKB) dataset is available via application at https://www.ukbiobank.ac.uk/. The Adolescent Brain Cognitive Development (ABCD) Study dataset is available through the NIMH Data Archive (NDA) at https://nda.nih.gov/. The Autism Brain Imaging Data Exchange (ABIDE) dataset is available at http://fcon_1000.projects.nitrc.org/indi/abide/. All datasets require registration and/or data use agreements for access.

Research involving human participants, their data, or biological material

Policy information about studies with human participants or human data. See also policy information about sex, gender (identity/presentation), and sexual orientation and race, ethnicity and racism.

Reporting on sex and gender	Sex was used as a biological variable and was binary-classified (male/female) based on the metadata provided by each consortium. Sex information in UKB was determined at recruitment; in ABCD and ABIDE, it was recorded by parent report. Gender was not separately assessed in this study. Sex-disaggregated sample sizes are reported in Table 2 (UKB: 21,556M/19,143F; ABCD: 4,635M/4,198F; ABIDE: 114M/27F). Sex classification was included as a downstream prediction task; no sex- or gender-based analyses of clinical outcomes were performed beyond controlling for sex as a covariate in confound analyses.
Reporting on race, ethnicity, or other socially relevant groupings	Race and ethnicity were not used as variables in this study. No socially constructed categorization variables were included in the analyses. Potential confounding from demographic heterogeneity was addressed through propensity score matching on sex, age, head motion, and acquisition site.
Population characteristics	See above. Demographic and clinical characteristics are detailed in Table 2, including age, sex, and disorder/control distributions for each dataset and task-specific subset.
Recruitment	All three datasets are large-scale, publicly available consortium studies with their own established recruitment protocols. UKB recruited ~500,000 UK adults aged 40–69 through the National Health Service. ABCD recruited ~11,800 U.S. children aged 9–10 across 21 sites. ABIDE aggregated existing resting-state fMRI data from 17 international sites. Site-related biases were addressed through propensity score matching and inclusion of site as a covariate.
Ethics oversight	This study used de-identified, publicly available datasets. UKB received ethical approval from the NHS National Research Ethics Service (Ref: 11/NW/0382). ABCD received centralized IRB approval from the University of California, San Diego. ABIDE data were collected under local IRB approval at each contributing site, with all data fully anonymized before public release.

Note that full information on the approval of the study protocol must also be provided in the manuscript.

Field-specific reporting

Please select the one below that is the best fit for your research. If you are not sure, read the appropriate sections before making your selection.

☒ Life sciences ☐ Behavioural & social sciences ☐ Ecological, evolutionary & environmental sciences

For a reference copy of the document with all sections, see nature.com/documents/nr-reporting-summary-flat.pdf

Life sciences study design

All studies must disclose on these points even when the disclosure is negative.

Sample size	No a priori sample size calculation was performed. Sample sizes were determined by the availability of quality-controlled resting-state fMRI data in each publicly available consortium dataset after applying standardized exclusion criteria (UKB: N=40,699; ABCD: N=8,833; ABIDE: N=141). These large-scale datasets are well-established benchmarks in the neuroimaging community, and the UKB and ABCD cohorts substantially exceed the minimum sample sizes recommended for reproducible brain-wide association studies (Marek et al., Nature, 2022).
Data exclusions	Data exclusions were pre-established and applied uniformly across datasets. Subjects were excluded if: (1) preprocessing failed due to spatial incompatibility or empty brain masks (UKB: 3,242; ABCD: 2,198; ABIDE: 161), (2) atlas-based ROI extraction produced invalid signals, defined as all-zero time series in one or more ROIs (UKB: 86; ABCD: 567; ABIDE: 773), or (3) required metadata (sex or diagnosis) were missing (UKB: 7; ABCD: 6; ABIDE: 0). For task-specific analyses, participants with missing or invalid assessment scores were further excluded via listwise deletion. Full attrition details are provided in Figure 4.
Replication	All experiments were repeated across three random seeds, and results are reported as mean ± SEM. Key findings were validated across two independent brain atlases (HCP-MMP1 and Schaefer 400). The frequency threshold stability was confirmed via 1,000-iteration bootstrap resampling and HCP test–retest analysis. All attempts at replication were successful.
Randomization	This is an observational study using pre-existing clinical diagnoses; participants were not experimentally allocated to groups. Disorder and control groups were defined by established clinical criteria (e.g., CBCL T-score >65 for ADHD). Covariates including sex, age, head motion, and acquisition site were controlled through propensity score matching. Data were split into training (70%), validation (15%), and test (15%) sets using stratified sampling to preserve class distributions.
Blinding	Blinding was not relevant to this study as all analyses were conducted using automated computational pipelines with pre-defined hyperparameters and evaluation metrics. Group labels were used only for model training and evaluation; model selection was based on validation set performance, and all reported results are from held-out test sets not used during model development.

Reporting for specific materials, systems and methods

We require information from authors about some types of materials, experimental systems and methods used in many studies. Here, indicate whether each material, system or method listed is relevant to your study. If you are not sure if a list item applies to your research, read the appropriate section before selecting a response.

Materials & experimental systems

n/a	Involved in the study
☒	☐ Antibodies
☒	☐ Eukaryotic cell lines
☒	☐ Palaeontology and archaeology
☒	☐ Animals and other organisms
☐	☒ Clinical data
☒	☐ Dual use research of concern
☒	☐ Plants

Methods

n/a	Involved in the study
☒	☐ ChIP-seq
☒	☐ Flow cytometry
☐	☒ MRI-based neuroimaging

Clinical data

Policy information about clinical studies

All manuscripts should comply with the ICMJE guidelines for publication of clinical research and a completed CONSORT checklist must be included with all submissions.

Clinical trial registration: Not applicable. This study is a retrospective analysis of existing publicly available datasets, not a clinical trial.

Study protocol: Not applicable. This is not a clinical trial. The full computational methodology and analysis pipeline are described in the Methods section of the manuscript, and all code is publicly available at https://github.com/Transconnectome/MBBN.

Data collection: This study used three publicly available, previously collected datasets. UKB: resting-state fMRI collected across multiple imaging centers in the UK (2014–ongoing; TR=0.735s). ABCD: baseline year-one resting-state fMRI collected across 21 sites in the United States (2016–2018; TR=0.8s). ABIDE: resting-state fMRI aggregated from 17 international sites (2006–2012; TR=1.5–3.0s). Detailed acquisition parameters are reported in Table 3. No new data were collected for this study.

Outcomes: Primary outcome measures were pre-defined before model training: AUROC for classification tasks (sex, ADHD, ASD, depression diagnosis) and MAE for regression tasks (fluid intelligence, depression severity). Secondary outcomes included frequency-specific connectivity patterns identified through GradCAM-based attribution analysis. The classification threshold was pre-determined on the validation set by maximizing the geometric mean of sensitivity and specificity, then applied to the held-out test set.

Plants

Seed stocks: *Report on the source of all seed stocks or other plant material used. If applicable, state the seed stock centre and catalogue number. If plant specimens were collected from the field, describe the collection location, date and sampling procedures.*

Novel plant genotypes: *Describe the methods by which all novel plant genotypes were produced. This includes those generated by transgenic approaches, gene editing, chemical/radiation-based mutagenesis and hybridization. For transgenic lines, describe the transformation method, the number of independent lines analyzed and the generation upon which experiments were performed. For gene-edited lines, describe the editor used, the endogenous sequence targeted for editing, the targeting guide RNA sequence (if applicable) and how the editor was applied.*

Authentication: *Describe any authentication procedures for each seed stock used or novel genotype generated. Describe any experiments used to assess the effect of a mutation and, where applicable, how potential secondary effects (e.g. second site T-DNA insertions, mosiacism, off-target gene editing) were examined.*

Magnetic resonance imaging

Experimental design

Design type: Resting state.

Design specifications: All datasets used resting-state fMRI with a single continuous scan per session. UKB: 490 volumes (6 min). ABCD: 368 volumes per run (5 min; baseline year-one data used). ABIDE: variable across sites (128–280 volumes; 5–10 min). The first 20 volumes were discarded from all datasets to minimize scanner instability effects.

Behavioral performance measures: Not applicable. All data were acquired during resting state with no behavioral task. Clinical and cognitive measures used as prediction targets (CBCL T-scores for ADHD, PHQ-9 for depression, NIH Toolbox Fluid Intelligence) were collected separately by each consortium outside the scanner.

Acquisition

Imaging type(s)	Functional (resting-state BOLD fMRI).
Field strength	UKB: 3T (Siemens Skyra). ABCD: 3T (Siemens Prisma, GE 750, Philips). ABIDE: 3T (various scanners across 17 sites).
Sequence & imaging parameters	UKB: gradient echo EPI, TR=0.735s, TE=39ms, voxel size 2.4×2.4×2.4mm³, multiband factor 8. ABCD: gradient echo EPI, TR=0.8s, TE=30ms, voxel size 2.4×2.4×2.4mm³, multiband factor 6. ABIDE: gradient echo EPI, TR=1.5–3.0s, TE=various, voxel size=various across sites. Full parameters are summarized in Table 3.
Area of acquisition	Whole brain.

Diffusion MRI ☐ Used ☒ Not used

Preprocessing

Preprocessing software	fMRIPrep (Esteban et al., 2019) was used for skull-stripping, slice-timing correction, susceptibility distortion correction, and spatial normalization. ROI time series were extracted using Nilearn. Frequency decomposition was performed using nitime (FIR filters) and custom Boxcar filters. Software versions: Python 3.10, PyTorch 2.6.0, nibabel 5.3.2, nitime 0.11, scikit-learn 1.6.1.
Normalization	Nonlinear spatial normalization to MNI standard space was performed by fMRIPrep using ANTs SyN registration.
Normalization template	MNI152NLin2009cAsym (MNI space).
Noise and artifact removal	White matter signals and head motion artifacts were removed using component-based noise correction (CompCor; Behzadi et al., 2007). The first 20 volumes of each time series were discarded to minimize T1 equilibration effects. Dataset-specific bandpass filters were applied: ABCD 0.009–0.08 Hz, UKB 0.008–0.1 Hz, ABIDE 0.01–0.1 Hz (FIR).
Volume censoring	No explicit volume censoring (scrubbing) was applied. Head motion was controlled by CompCor denoising and by including mean framewise displacement (FD) as a covariate in confound analyses and propensity score matching."

Statistical modeling & inference

Model type and settings	Predictive deep learning model (MBBN): a transformer-based architecture combining a shared BERT temporal encoder (8 hidden layers, 8 or 12 attention heads) with frequency-specific spatial attention modules. Training used cross-entropy loss for classification and L1 loss for regression, combined with a spatial regularization loss. Hyperparameters: learning rates [1e-5, 1e-2], optimizers (Adam, AdamW, RMSprop), weight decay [1e-3, 1e-2]. Group-level statistical comparisons used two-sample t-tests with FDR correction.
Effect(s) tested	The primary effects tested were prediction of clinical diagnosis (ADHD, ASD, depression), biological sex, and cognitive ability (fluid intelligence) from resting-state fMRI. Secondary analyses tested group differences (disorder vs. healthy controls) in frequency-specific attention-derived connectivity patterns using t-tests. No factorial designs were used.

Specify type of analysis: ☐ Whole brain ☒ ROI-based ☐ Both

Anatomical location(s): HCP-MMP1 360 parcels, Schaefer 400 parcels

Statistic type for inference (See Eklund et al. 2016)	ROI-based. All analyses were conducted on parcellated time series extracted from predefined atlases (HCP-MMP1: 360 ROIs; Schaefer: 400 ROIs). No voxel-wise or cluster-wise inference was performed.
Correction	False Discovery Rate (FDR) correction using the Benjamini-Hochberg method was applied to all group-level connectivity comparisons (FDR-corrected p < 0.05). Effect sizes were quantified using Cohen's d.

Models & analysis

n/a	Involved in the study
☐	☒ Functional and/or effective connectivity
☐	☒ Graph analysis
☐	☒ Multivariate modeling or predictive analysis

Functional and/or effective connectivity	Two connectivity measures were used: (1) Pearson correlation between ROI time series for traditional functional connectivity (used as input for baseline models and for computing communicability in pretraining), and (2) self-attention-weighted connectivity derived from MBBN's spatial attention module, which captures nonlinear, dynamic inter-regional dependencies optimized for downstream prediction tasks. Correlation and complementarity between the two measures were assessed using Spearman correlation and Kendall tau (Supplementary Table 2).
Graph analysis	Communicability was computed on binarized functional connectivity graphs derived from Pearson correlation matrices (subject-level). Communicability scores were used to rank node importance for the

pretraining masking strategy. Network properties (modularity, small-worldness parameters γ, λ, σ) were computed at the group level on both traditional FC matrices and attention-derived connectivity matrices across three frequency bands, using weighted graphs (Supplementary Table 1).

Multivariate modeling and predictive analysis

Independent variables: frequency-decomposed ROI time series (ultralow, low, high bands) extracted from HCP-MMP1 (360 ROIs) or Schaefer (400 ROIs) atlases. No additional feature extraction or dimension reduction was applied; raw parcellated time series were input directly to the model. Model: MBBN, a transformer-based architecture with shared BERT temporal encoder and frequency-specific spatial attention modules, pretrained with communicability-based masking on UKB and fine-tuned on target datasets. Training: 70/15/15 train/validation/test split with stratified sampling; 3 random seeds; gradient clipping (norm=1); AMP; AdamW optimizer. Evaluation metrics: AUROC, balanced accuracy, sensitivity, specificity, and F1-score for classification; MAE, MSE, NMSE, and R^2 for regression. Model selection was based on validation set performance.
