# Supplementary material for: Learning brain dynamics across distinct scaling regimes reveals psychiatric signatures
Source: Commun Biol. 2026 May 8;9:963. doi: 10.1038/s42003-026-10011-7 (PMC13369815; doi:10.1038/s42003-026-10011-7)
Supplement: Supplementary file 5 — Transparent Peer Review file [file 42003_2026_10011_MOESM5_ESM.pdf]

nature portfolio

Peer Review File

Frequency-specific attention reveals spatiotemporal brain dynamics in fMRI

Corresponding Author: Professor Jiook Cha

This manuscript has been previously submitted at another journal. This document only contains information relating to versions considered at Communications Biology.

This file contains all reviewer reports in order by version, followed by all author rebuttals in order by version.

Version 0:

Reviewer comments:

Reviewer #1

(Remarks to the Author)
The authors introduce Multi-Band Brain Net (MBBN), a novel transformer-based model for analyzing frequency-specific spatiotemporal fMRI data. The work is methodologically sophisticated and demonstrates strong classification performance in large-scale neuroimaging datasets, particularly for ADHD and ASD. However, the manuscript requires significant revisions to address conceptual clarity, methodological rigor, and broader applicability before it can be considered for publication.
Major concerns:
1. While MBBN achieves notable gains in classification tasks (e.g., sex, ADHD, ASD), its performance in regression (e.g., fluid intelligence, depression) is marginal or even inferior to some baselines (e.g., fluid intelligence in UKB). It would be better to:
discuss why MBBN underperforms in regression contexts; clarify whether architectural components favor categorical rather than continuous targets and consider architectural modifications or analyses better suited for continuous variables.
2. The manuscript relies heavily on Lorentzian and spline-based knee detection to define frequency bands (f1, f2), yet:
These thresholds are data-driven but not cross-validated across subjects or datasets.
There is no evidence of their physiological stability across time or populations.
The bands do not align with canonical neurophysiological frequencies (e.g., delta, theta).
Authors should validate whether these frequency divisions are consistent, interpretable, and generalizable. Alternatively, consider data-driven or adaptive band definitions (e.g., wavelet-based, learned filters).
3. The model interprets attention matrices as direct analogs to functional connectivity. However, recent work cautions against interpreting transformer attention weights as meaningful dependencies. It would be better to acknowledge the limitations of this interpretation and include a cautionary discussion on the use of attention as connectivity, or empirically validate this assumption.
4. While the Grad-CAM-inspired analysis is innovative, it is unclear:
How stable or consistent these highlighted connections are across subjects.
Whether these findings replicate known biomarkers or are novel (and testable).
Minor concerns:
5. The reported AUROC gains are large in some cases (e.g., 52.5%), but statistical significance is only implied. Include formal significance testing (p-values, confidence intervals) for performance comparisons.
6. The manuscript makes strong claims about "lifespan-inclusive" or "precision psychiatry" applications. However, no elderly or neurodegenerative datasets were used. Such claims should be moderated unless validated with such cohorts.
7. Many terms (e.g., “CLS token,” “communicability masking,” “spatial loss”) are introduced without sufficient explanation. For Communications Biology, a broader audience explanation is needed.
8. The model uses multiple transformer modules and custom losses. Pretraining spans 1000 epochs. The authors should discuss: model efficiency and feasibility for labs without high-end computing resources. And possible simplifications or distillations of the model for practical use.

Reviewer #2

(Remarks to the Author)
In this paper, the authors introduce Multi-Band Brain Net (MBBN), the first transformer framework to model fMRI data as biologically grounded, scale-free, frequency-specific spatiotemporal dynamics: it decomposes each time-series into ultralow, low and high bands via Lorentzian-plus-spline fitting, feeds the bands through a shared temporal BERT and frequency-specific multi-head spatial attention, and pretrains by masking high-communicability nodes to learn network hubs. Trained on 49 k UKB/ABCD/ABIDE subjects, MBBN sets new state-of-the-art in sex, ADHD and ASD classification (up to 52 % AUROC gain) and reveals disorder biomarkers—ADHD shows attenuated high-frequency fronto-sensorimotor and compensatory ultralow parahippocampal connectivity, while ASD exhibits ultralow temporo-parietal hyperconnectivity and low-frequency frontopolar disruption. Ablation studies confirm that both frequency division and communicability masking are essential, attention-derived connectivity captures complementary information to Pearson FC, and the model generalizes across ages and atlases, providing an open-source, interpretable foundation for precision psychiatry.

comments:
1. How sensitive are the frequencies f_1 and f_2 to age, sex, head motion, and multi-site acquisition differences? Could you provide an age-matched subsample analysis or motion-corrected re-estimation to ensure that the reported disorder effects are not driven by these confounds?
2. The paper reports test-set AUROCs but not test-retest reliability. Could you compute the intraclass correlation (ICC) or Pearson r of the frequency-specific attention matrices for the 40 UKB subjects with repeat scans to demonstrate temporal stability of the biomarkers?
3.The spatial loss λ is tuned per dataset (1–100). Can you provide a principled heuristic—perhaps based on the ratio of task loss gradient norms to spatial loss gradient norms—to set λ automatically, and does this heuristic generalize to new cohorts without grid-search?
4.the current work uses resting-state data. How would MBBN handle task-evoked fMRI where the power-law assumption may be transiently violated?

Reviewer #3

(Remarks to the Author)
In this study, the authors underscore the significance of the brain's nonlinear dynamics in the context of cognitive neuroscience and, accordingly, propose a Multiband Brain Network (MBBN) framework to examine frequency-specific brain connectivity using functional magnetic resonance imaging (fMRI) data. The proposed method decomposes fMRI signals into three distinct frequency bands and integrates information across these bands to enhance performance in classifying various brain disorders and predicting cognitive and mental health outcomes. Overall, the manuscript presents an interesting concept with potentially impactful results. However, the paper suffers from issues in organization and clarity, particularly in the presentation of results. Several substantial weaknesses are evident, including an overly inclusive, "kitchen sink" methodological approach; vague or poorly defined modules within the proposed framework; insufficient justification for the selection of evaluation metrics; and a lack of clarity regarding the selection and preprocessing of data from different datasets. Below, I outline several major concerns and suggestions for improvement.

1. Task Selection Criteria
A primary concern is the lack of clarity regarding the selection criteria for tasks used in the analyses. For instance, in the ABCD dataset, depressive symptom scores are available and could be utilized for regression analyses, yet these are not addressed. Similarly, in the ABIDE dataset, clinically relevant behavioral metrics such as the Social Responsiveness Scale (SRS) and Autism Diagnostic Observation Schedule (ADOS) scores are available, but their exclusion is neither justified nor explained.
2. Presentation of Results
The results section is poorly structured, requiring readers to frequently cross-reference earlier sections to understand the definitions and findings. Critical information—such as the specific fluid intelligence metric used and the handling of missing (NaN) assessments—is not clearly introduced or described, which significantly hampers interpretability.
3. Limited Quantitative Results
The quantitative findings are limited to Table 1 and Figures 2 and 3, which is insufficient for a study of this scope. Moreover, Table 1 is not cited or discussed in the main text, and no statistical comparisons (e.g., significance testing) are provided for the differences observed in Figure 2. This omission raises concerns regarding the robustness and interpretability of the reported results.
4. Inadequate Visualization of Connectivity Alterations
Figure 3 attempts to illustrate connectivity alterations associated with ASD and ADHD; however, it is not effective in conveying spatial information. Despite the accompanying textual description, the figure lacks anatomical clarity and does not allow readers to easily localize the abnormalities. Furthermore, it is unclear why only ASD and ADHD are shown, while other regression outcomes are omitted without explanation.
5. Interpretation of Improvement Rates
The manuscript emphasizes improvement rates as a key performance metric. However, in large-scale datasets such as UKB and ABCD, effect sizes in regression tasks are known to be small (e.g., Cohen's d ≈ 0.2). An apparent 10% improvement may correspond to a trivial increase in correlation (e.g., from 0.10 to 0.11), which has limited clinical or practical significance. The authors should provide a more nuanced discussion of these limitations.
6. Inconsistent Preprocessing and Filtering
Given that the proposed framework aims to capture frequency-specific information, it is surprising that temporal filtering was applied during preprocessing. More critically, the filtering parameters differ across datasets, which is methodologically

inconsistent and undermines the comparability of results across datasets.
7. Lack of Correction for Imaging Artifacts and Temporal Resolution Discrepancies
The authors do not address whether distortion correction was applied for the UKB and ABCD datasets. Furthermore, the ABIDE dataset has a significantly different temporal resolution compared to UKB and ABCD. The potential influence of repetition time (TR) and other acquisition parameters on the proposed method is neither explored nor discussed.
8. Insufficient Dataset Description and Selection Criteria
The manuscript lacks a clear description of the datasets used. The reported sample sizes differ from those in previous studies, yet no rationale is provided for these discrepancies. For example, it is unclear whether the ABCD analysis uses baseline data, and why approximately 2,000 participants appear to have been excluded. For the ABIDE dataset, it is not specified whether ABIDE-I or ABIDE-II was used, whether data from a single site was selected, or what criteria guided these decisions.

Version 1:

Reviewer comments:

Reviewer #1

(Remarks to the Author)
Thanks authors.

Reviewer #2

(Remarks to the Author)
The authors had solved my comments. The Manuscript can be accepted by this journal.

Reviewer #3

(Remarks to the Author)
My primary concern pertains to the reported sample sizes and the transparency of the subject selection procedures. The revised manuscript continues to exhibit substantial inconsistencies and a lack of clarity. For instance, the authors state vthat the UK Biobank (UKB) dataset, which includes 41,283 participants, was used for pretraining due to its large sample size and broad demographic representation. This extensive dataset enabled the model to capture generalizable brain dynamics patterns. The Adolescent Brain Cognitive Development (ABCD) dataset, consisting of 4,527 participants (including individuals with ADHD and healthy controls), and the Autism Brain Imaging Data Exchange (ABIDE) dataset, which includes 141 participants (comprising individuals with ASD and healthy controls), were employed for fine-tuning to capture disorder-specific patterns. However, these figures do not align with the values reported in Table 2. Moreover, Table 2 appears to indicate that the depression and ADHD cohorts exceed the size of the healthy control cohorts—particularly for depression—which is counterintuitive and requires justification. In addition, it is unclear why the total ABCD sample size reported elsewhere (e.g., 8,833) does not reconcile with the subset counts for depression and ADHD; what categories or exclusions account for the discrepancy? Finally, the reported ABIDE sample of 141 subjects is difficult to reconcile with the known size of ABIDE I (i.e., >1,100 subjects). The manuscript does not specify the inclusion/exclusion criteria that would remove over 90% of the available data.
To address these issues, the authors should provide a detailed, stepwise account of participant selection and attrition for each dataset, ideally as both a flowchart and a corresponding textual description. At a minimum, the following should be reported for each cohort (UKB, ABCD, ABIDE):
• Initial sample size available.
• Sequential exclusion criteria with counts at each step (e.g., head motion thresholds; image quality/spatial artifact metrics; missing demographic or clinical data; scan acquisition deviations; preprocessing failures; diagnostic adjudication and comorbidity exclusions).
• Final analytic sample size, stratified by diagnostic group and healthy controls.
• Rationale and thresholds for all quality-control criteria, with references to established standards where applicable.
• Consistency checks ensuring that numbers in the main text match those in tables and supplementary materials.
An example of the expected level of detail (illustrative only) is as follows: “ABCD initial N ≈ 11,000; excluded N = XX for excessive motion (framewise displacement > 0.3 mm or > 20% scrubbed volumes), N = XX for spatial quality failures (e.g., low tSNR, registration failure), N = XX for missing or unreliable diagnostic phenotypes, N = XX for incomplete covariates (age, sex, site), N = XX due to protocol deviations, yielding final N = XXXX (ADHD = XXX; depression = XXX; healthy controls = XXX).” Similar details should be provided for UKB and ABIDE, including a justification for the drastic reduction of ABIDE I to 141 subjects, with explicit thresholds and site-level considerations.
Such reporting aligns with expectations in high-impact journals and is essential for reproducibility and independent verification of the findings.
The authors also need to carefully double-check the manuscript to make sure the overall quality or at least consistency.

Open Access This Peer Review File is licensed under a Creative Commons Attribution 4.0 International License, which permits use, sharing, adaptation, distribution and reproduction in any medium or format, as long as you give appropriate credit to the original author(s) and the source, provide a link to the Creative Commons license, and indicate if changes were made.
In cases where reviewers are anonymous, credit should be given to 'Anonymous Referee' and the source.
The images or other third party material in this Peer Review File are included in the article's Creative Commons license, unless indicated otherwise in a credit line to the material. If material is not included in the article's Creative Commons license and your intended use is not permitted by statutory regulation or exceeds the permitted use, you will need to obtain permission directly from the copyright holder.
To view a copy of this license, visit https://creativecommons.org/licenses/by/4.0/

Dear Reviewer 1,

We would like to express our sincere gratitude for your constructive and detailed feedback on our manuscript. Your insightful comments have been invaluable in helping us to improve the quality of the paper. Below, we provide our detailed responses.

Major Concerns

---

1. Comment about regression performance

- **Reviewer's Comment:** While MBBN achieves notable gains in classification tasks (e.g., sex, ADHD, ASD), its performance in regression (e.g., fluid intelligence, depression) is marginal or even inferior to some baselines (e.g., fluid intelligence in UKB). It would be better to: discuss why MBBN underperforms in regression contexts; clarify whether architectural components favor categorical rather than continuous targets and consider architectural modifications or analyses better suited for continuous variables.

- **Our Response:** We sincerely thank the reviewer for this insightful and important comment. We agree that our manuscript required a more nuanced analysis of the model's regression performance, and your feedback has been invaluable in strengthening our Discussion section.

  To address your points, we have revised the Discussion to provide a comprehensive, two-part explanation for this limitation:

  - **Primary Reason (Label Quality):** We first clarify that the regression targets were derived from inherently noisier, single-modality measures (e.g., PHQ-9 questionnaire, NIH Toolbox scores). This is in contrast to the biologically grounded (e.g., sex) or multifaceted labels (e.g., K-SADS diagnoses) used for classification.

  - **Compounding Factor (Model Bias):** We then explain that this data-level challenge is likely compounded by the intrinsic bias of transformers. As the literature suggests, their architecture is highly effective for learning clear classification boundaries but can be less optimal for approximating the smooth functions required for noisy, continuous variables.

  Based on this analysis, we also now propose future architectural enhancements (e.g., Smoothness Regularization, Hybrid Architectures) to better address continuous targets. We are grateful for your feedback, which has led to a much clearer and more thorough discussion of our model's specific strengths and limitations.

- **Revised Manuscript**:
    - To address the reviewer’s concern about the model’s regression performance, we have revised the Discussion section to provide a more comprehensive analysis of this limitation. We now explain that the performance difference is likely due primarily to the inherently noisier nature of the regression labels (derived from single measures) compared to the robust diagnostic labels used for classification. We further discuss how this data-level challenge is compounded by the transformer's intrinsic bias and suggest future architectural improvements, such as Smoothness Regularization or a Hybrid Architecture.
        - [Discussion Section Paragraph 12] “*Second, a limitation pertains to the model’s modest performance on regression tasks. We attribute this primarily to the nature of the regression targets (e.g., scores from single questionnaires or behavioral tests), which may have lower signal-to-noise ratio or reliability compared to the more robust diagnostic labels (e.g., KSADS) or biologically grounded label (e.g., sex)used for classification. This data-level challenge is likely compounded by the intrinsic bias of transformers to learn non-smooth functions, which may be less optimal for capturing the subtle variations in continuous phenotypes”*
        - [Discussion Section Paragraph 13] *“In addition, future research could explore architectural enhancements---such as Smoothness Regularization or a Hybrid Architecture—to further improve regression performance, complementing the model’s strong classification capability.”*

---

2. Comment about validation of frequency-dividing

- **Reviewer's Comment**: The manuscript relies heavily on Lorentzian and spline-based knee detection to define frequency bands (f1, f2), yet: These thresholds are data-driven but not cross-validated across subjects or datasets. There is no evidence of their physiological stability across time or populations. The bands do not align with canonical neurophysiological frequencies (e.g., delta, theta). Authors should validate whether these frequency divisions are consistent, interpretable, and generalizable. Alternatively, consider data-driven or adaptive band definitions (e.g., wavelet-based, learned filters).
- **Our Response & Revised Texts**
    - We sincerely thank the reviewer for their thoughtful and constructive feedback. We found the comments to be invaluable and have thoroughly revised the manuscript in response. The seven main revisions, which we believe substantially strengthen the paper's methodological rigor, are summarized below, followed by a detailed point-by-point response.
        - **(A)** We conducted new bootstrap, test-retest, and perturbation analyses to empirically confirm the subject-level robustness and stability of our method.
        - **(B)** We demonstrated that cross-dataset differences in our frequency bands are explained by acquisition physics (TR/Nyquist), with the relative band structure remaining consistent.
        - **(C)** We performed a sensitivity analysis showing that model performance is robust and stable even when frequency boundaries are perturbed by up to ±10%.
        - **(D)** We confirmed the high test-retest reliability of our frequency thresholds, showing they are physiologically stable across different scanning sessions for the same individuals.
        - **(E)** We benchmarked our data-driven approach against conventional fixed fMRI bands and showed it achieves superior predictive performance.
        - **(F)** As requested, we implemented an alternative adaptive method (k-means clustering) and showed our knee-based approach performed favorably.
        - **(G)** We used a large-scale, 1,000-iteration bootstrapping procedure to validate that our method is reproducible, generalizable, and stable at the population level.
    - **(A) Subject-level robustness via bootstrap resampling.** We thank the reviewer for this critical point regarding the stability and generalizability of our data-driven frequency bands. To empirically address this concern, we have

performed a series of new validation experiments and incorporated the results into the manuscript. Specifically, we conducted:

1. Bootstrap Resampling: To assess population stability, we ran a comprehensive bootstrap analysis across subject subgroups.
2. Test-Retest Analysis: To evaluate temporal stability, we conducted a test-retest reliability analysis using the HCP dataset.
3. Boundary Perturbation Analysis: To confirm the robustness of our method, we performed a new sensitivity analysis by systematically perturbing the frequency boundaries.

These newly added analyses consistently demonstrate that our learned knee frequencies are stable across populations, time, and minor parameter variations.

- [Methods section 3.4.4 Paragraph 2] *"In addition to the TR/Nyquist effects summarized in Table 4, we verified that the learned knees are robust along three axes: population, measurement time, and boundary shifts. First, bootstrap resampling across subgroups revealed no significant differences in f1 or f2 (p = 0.6541 and p = 0.8956). Second, an HCP test–retest analysis (run–1 vs. run–2) showed no significant shifts (p = 1.000 for f1; p = 0.9428 for f2). Third, perturbing each cutoff by ±5–10% yielded a broad performance plateau centered at the learned boundaries (peak AUROC = 0.916), indicating insensitivity to small boundary changes. Full procedures and results are provided in Supplementary Materials F.4, F.5."*

○ **(B) Cross-dataset differences are explained by acquisition physics (TR/Nyquist).** Given that the UKB, ABCD, and ABIDE cohorts feature different acquisition parameters, it is crucial to verify that our frequency-division method produces consistent and interpretable results despite these variations. We therefore analyzed the relationship between the empirically derived knee frequencies and the primary acquisition parameter governing the spectral range, the repetition time (TR).

○ [Methods section 3.4.4 Paragraph 1] *"Table 4 reports the dataset-wise knee frequencies (f1, f2; mean ± s.d.), together with the acquisition repetition time (TR) and the corresponding Nyquist limit (Nyquist = 1/(2·TR)). As expected, absolute knee values differ across datasets because the analyzable frequency range is physically bounded by TR: ABCD (TR = 0.735 s; Nyquist = 0.6803 Hz) and UKB (TR = 0.8 s; Nyquist = 0.625 Hz) exhibit higher f1/f2 than ABIDE, which spans longer TRs (1.5–3.0 s; Nyquist = 0.1667–0.3333 Hz). Importantly, when expressed relative to the Nyquist limit, the knees occupy comparable portions of the spectrum across datasets—f1 ≈ 5–10% and f2 ≈ 9–18% of Nyquist. This pattern indicates that our data-driven procedure adapts to each dataset's spectral support while preserving a consistent relative band structure, in line with the TR/Nyquist constraints of fMRI acquisitions."*

○ **(C) Sensitivity analysis shows an accuracy plateau around the learned boundaries.** To verify that our model's performance is not critically dependent on the exact values of the frequency cutoffs, we conducted a sensitivity analysis. We systematically tested the model's robustness by introducing minor perturbations to the empirically-derived knee frequencies (f1 and f2)

○ [Supplementary Materials F.4 Whole Paragraphs] *"To assess the robustness of our model to minor variations in the data-driven frequency cutoffs, we conducted a sensitivity analysis. We systematically perturbed the empirically derived knee frequencies (f1 and f2) by ±5% and ±10% and re-evaluated the model's performance on the ABCD sex classification task. The four perturbation conditions were:*

• Wider: The frequency band was widened by shifting f1 down by 10% (or 5%) and f2 up by 10% (or 5%).

• Narrower: The band was narrowed by shifting f1 up and f2 down.

• Shift Up: Both f1 and f2 were shifted up.

• Shift Down: Both f1 and f2 were shifted down.

The results, summarized in Supplementary Table F9, demonstrate that the model's performance is highly stable across all conditions. Even with a ±10% perturbation to the frequency boundaries, the mean AUROC remained within a tight range (0.902 to 0.908), showing no significant degradation compared to the performance with the original, unperturbed cutoffs. This analysis confirms that our framework is not dependent on the exact precision of the knee frequency detection and is robust to small variations in the spectral characteristics of the data."

○ **(D) Test–retest (physiological) stability across sessions.** In addition to assessing robustness, we evaluated the test-retest reliability of our method to ensure that the derived knee frequencies represent stable individual traits rather than session-dependent noise. To this end, we examined the temporal consistency of f1 and f2 for the same subjects across two independent fMRI runs.

○ [Supplementary Materials F.5 Whole Paragraphs] *"We estimated the data-driven knee frequencies (f1, f2) for each subject in the HCP resting-state fMRI LR runs (run-1 and run-2) using the same pipeline as in the main analyses. A paired two-sided test found no evidence of run effects (f1: $p = 1.000$; f2: $p = 0.9428$), supporting short-interval test–retest stability of the knee frequencies."*

- **(E) Relation to canonical EEG bands and prior fMRI banding.** To situate our physiologically-grounded, data-driven approach within the context of established practices, we benchmarked its performance against several alternative frequency division strategies. These included both conventional, literature-based fixed fMRI bands (e.g., Slow-5: 0.01–0.027 Hz; Slow-4: 0.027–0.073 Hz).

- [Method section 3.4.6, paragraph 1, 2] *"Our frequency decomposition is based on a physiologically grounded, data-driven characterization of the scale-free fMRI spectrum: we fit Lorentzian components and locate spectral knees with a spline procedure to obtain dataset-specific cutoffs (f1, f2). We compared this approach with two widely used alternatives. First, a fixed-band scheme (Slow-5: 0.01–0.027 Hz; Slow-4: 0.027–0.073 Hz) ([29]) yielded a modest but consistent drop in performance for the same tasks (AUROC = 0.911 vs. 0.916 with our knees; Supplementary Materials F.3)."*

- [Supplementary Materials F.3, paragraph 1, 2] *"To validate our theory-driven frequency division strategy, we systematically compared its performance against several alternative approaches. As detailed in Supplementary Table F8, our proposed method ('Ours') achieved the highest AUROC (0.916) and Accuracy (0.844) on the ABCD sex classification task. Notably, our approach outperformed not only baseline models using no frequency division but also other data-driven techniques, such as a wavelet-based method ('Wavelet & KNN'). Furthermore, it demonstrated a clear advantage over conventional, literature-based fixed bands ('Canonical'), underscoring the benefit of our individually tailored approach. This comprehensive comparison confirms that defining three distinct frequency bands using our Lorentzian and spline-based method provides a more powerful and informative representation for predicting phenotypes from fMRI time-series data."*

- **(F) Adaptive alternatives (as requested): power-weighted K-means + wavelet.** In addition to comparing our model against predefined fixed bands, we benchmarked it against an alternative adaptive data-driven approach. This second comparison tests our knee-based method against a different empirical strategy that uses spectral clustering to define its frequency boundaries.

- [Method section 3.4.6, paragraph 1, 3] *"Our frequency decomposition is based on a physiologically grounded, data-driven characterization of the scale-free fMRI spectrum: we fit Lorentzian components and locate spectral*

knees with a spline procedure to obtain dataset-specific cutoffs (f1, f2). We compared this approach with two widely used alternatives. Second, we implemented an adaptive alternative approach that (i) computes the Welch power spectral density, (ii) clusters frequencies into three groups via power-weighted k-means, (iii) uses the midpoints between adjacent clusters as two cutoffs, and (iv) performs wavelet-based decomposition using those data-driven boundaries ([30]). This adaptive variant achieved AUROC = 0.905, broadly consistent yet modestly below our knee-based method (Supplementary Materials F.3)."

- [Supplementary Materials F.3, paragraph 1, 2] *"To validate our theory-driven frequency division strategy, we systematically compared its performance against several alternative approaches. As detailed in Supplementary Table F8, our proposed method ('Ours') achieved the highest AUROC (0.916) and Accuracy (0.844) on the ABCD sex classification task. Notably, our approach outperformed not only baseline models using no frequency division but also other data-driven techniques, such as a wavelet-based method ('Wavelet & KNN'). Furthermore, it demonstrated a clear advantage over conventional, literature-based fixed bands ('Canonical'), underscoring the benefit of our individualized, scale-free approach. This comprehensive comparison confirms that defining three distinct frequency bands using our Lorentzian and spline-based method provides a more powerful and informative representation for predicting phenotypes from fMRI time-series data."*

- **(G) Large-N subsample vs full-cohort validation.** To confirm that our data-driven frequency thresholds are stable properties of the population and not artifacts of our specific subject sample, we performed a rigorous non-parametric bootstrapping analysis. This procedure was designed to test the stability and reproducibility of the threshold distributions (f1 and f2) when estimated from random subsamples of the cohort.

- [Method section 3.4.5, whole paragraph] *"To rigorously evaluate the stability and reproducibility of our data-driven frequency thresholds (f1 and f2), we performed a non-parametric bootstrapping procedure with 1,000 iterations. In each iteration, we randomly sub-sampled 80% of the subjects from the original cohort and re-calculated the subject-specific f1 and f2 distributions. We then assessed the stability of both the central tendency and the overall distributional shape. The central tendencies of the thresholds demonstrated exceptional stability; the distribution of differences between the sampled means and the original population means was tightly centered at zero with negligible deviation for both f1 and f2. Furthermore, to assess the stability of the entire distributional shape, we performed a two-sample Kolmogorov-Smirnov test for each iteration. The resulting p-values were overwhelmingly non-significant ($p > 0.05$ in over 95% of iterations for both*

thresholds), indicating that the distributions derived from the sub-samples were not statistically different from the original population's distribution. Taken together, these results confirm that our method for defining frequency bands is robust, reproducible, and generalizable across subjects, thus validating its use for the primary analyses in this study."

---

3. Comment about analysis of attention matrices

- **Reviewer's Comment:** The model interprets attention matrices as direct analogs to functional connectivity. However, recent work cautions against interpreting transformer attention weights as meaningful dependencies. It would be better to acknowledge the limitations of this interpretation and include a cautionary discussion on the use of attention as connectivity, or empirically validate this assumption.

- **Our Response**: We sincerely thank the reviewer for this crucial comment. We agree that interpreting transformer attention as a direct analog to functional connectivity (FC) requires careful validation, and we appreciate the caution. Following this valuable feedback, we have revised the manuscript to clarify our position. We now propose that MBBN's attention matrices are not intended to replicate FC, but rather represent a **new, qualitatively different connectivity metric** that is optimized for predictive tasks. Our rationale, which we have incorporated into the revised Methods 3.8.1, is that traditional FC is a static, linear measure that captures all temporal correlations (including non-informative noise), whereas our attention is a dynamic, non-linear weighting optimized to capture only **task-salient** interactions. This distinction is not just theoretical. As now detailed in the revised manuscript and supported by our analyses in **Supplementary Appendices B and C**, the two metrics exhibit fundamentally different topological properties and capture distinct, complementary information, especially following our frequency decomposition. We have updated Methods 3.8.1 to reflect this stronger, validated position, framing our attention matrix as a novel and meaningful connectivity metric in its own right, rather than just an interpretable feature validated against FC.

- **Revised Manuscript**:

  [Methods section 3.8.1, paragraph 3]

  "Crucially, we propose these attention patterns represent a novel, task-optimized connectivity metric that is qualitatively distinct from traditional FC. While traditional FC captures static, linear correlations—which may include non-informative physiological noise—MBBN's attention is a dynamic, non-linear weighting learned to solve a specific predictive task. Therefore, it is optimized to selectively capture inter-regional interactions that are salient for the prediction, while learning to suppress non-informative connections"

  [Methods section 3.8.1, paragraph 4]

"Therefore, the goal of this analysis is two-fold. First, to establish that these task-optimized attention matrices represent a new and valid connectivity metric that is distinct from traditional FC. Second, to validate that this new metric captures neurobiologically meaningful and clinically relevant biomarkers. This fundamental distinction from FC is empirically supported by our supplementary analyses:

• The two metrics show distinct topological properties. In the raw signal, traditional FC exhibits clear modularity, whereas the attention matrix shows virtually none. However, after our frequency decomposition, robust modularity emerges only in the attention matrices (Supplementary Table B1).

• The two metrics capture complementary, frequency-specific information. While they show a strong negative correlation in the raw signal, this correlation becomes markedly weaker in the frequency-specific bands (Supplementary Table C2)."

---

4. Comment about uncertainty of analysis of Grad-CAM

- **Reviewer's Comment**: While the Grad-CAM-inspired analysis is innovative, it is unclear: How stable or consistent these highlighted connections are across subjects. Whether these findings replicate known biomarkers or are novel (and testable).
- **Our Response**: We thank the reviewer for this insightful comment, which has helped us strengthen the validation of our model's interpretability. We agree that assessing the stability of the identified connections and clarifying their novelty are crucial.
    a. **Stability of Highlighted Connections:** To address the concern about stability, we conducted a new bootstrapping analysis. We generated 100 random subsamples of subjects and calculated the Jaccard Index for the top-100 discriminative connections identified in each subsample. The results showed excellent stability, with mean Jaccard indices of 0.940 (High-band), 0.900 (Low-band), and 0.920 (Ultralow-band). This confirms that the biomarkers identified by MBBN are highly consistent and robust across subjects. We have added a new subsection, "3.10.1. Stability and Consistency of Interpretability Analysis," to the Methods section detailing this procedure and its results.

    b. **Replication vs. Novelty of Findings:** To clarify the context of our findings, we have substantially revised the Discussion section (Section 4). We now explicitly delineate how our results both validate previously established neurobiological markers (e.g., attenuated high-frequency fronto-sensorimotor connectivity in ADHD; orbitofrontal disruptions in ASD) and, more importantly, generate novel, testable hypotheses. Specifically, we highlight two key novel insights derived from our frequency-specific approach: (1) the identification of Area OP2-3-VS as a potential dynamic hub in ADHD whose function shifts with oscillatory state, and (2) the discovery that hyperconnectivity in ASD is predominantly an ultralow-frequency phenomenon, offering a more precise biomarker than previously understood. These revisions now make the novel contributions of our work much clearer.

- **Revised Manuscript**:

  In response to the reviewer's valuable feedback, we have made the following key revisions to the manuscript:

  - [Method section 3.10.1, whole paragraph] *"To assess the stability and consistency of the connections identified by our GradCAM-inspired approach, we performed a rigorous bootstrapping analysis. We created 100 subsamples by randomly selecting 100 subjects from the test set for both the ADHD and ASD classification tasks. For each subsample, we repeated the group comparison and identified the top 100 most discriminative connections based on effect size (Cohen's d). The consistency of the identified connections across these subsamples was quantified using the Jaccard Index, which measures the similarity between sets. The analysis revealed a high degree of stability for the top-ranked connections in all frequency bands:*

    • High-frequency band: Jaccard Index = 0.940 ± 0.101

    • Low-frequency band: Jaccard Index = 0.900 ± 0.078

    • Ultralow-frequency band: Jaccard Index = 0.920 ± 0.035

    These high Jaccard Index values, consistently above 0.9, indicate that the discriminative connectivity patterns highlighted by our model are highly robust and not driven by a specific subset of subjects. This confirms that the identified biomarkers are stable features of the clinical groups."

  - [Discussion paragraph 11] *"Beyond this important validation, the unique architecture of MBBN enables the generation of novel, testable hypotheses by revealing the frequency-specific nature of these disruptions. The first novel insight relates to ADHD, where we identified Area OP2-3-VS as a dynamic hub whose connectivity patterns shift depending on the oscillatory state. This leads us to propose that a central mechanism of the disorder may be a disruption in the coordination of information flow, orchestrated by this hub's inability to flexibly manage frequency-dependent network communication. Secondly, our model offers a significant refinement to the hyperconnectivity theory in ASD. We discovered that the heightened connectivity between the temporo-parietal junction and prefrontal regions was not a broadband phenomenon but was predominantly observed in the ultralow-frequency band. This novel, frequency-specific finding suggests that atypical neural resource allocation in ASD is tied to specific, slow-timescale dynamics, providing a more precise biomarker than previously understood. By aligning transformer-based modeling with core neuroscientific principles, MBBN offers a scalable and biologically plausible framework for advancing our understanding of individualized brain dynamics."*

Minor Concerns

---

5. Comment about lack of statistical significance

- **Reviewer's Comment**: The reported AUROC gains are large in some cases (e.g., 52.5%), but statistical significance is only implied. Include formal significance testing (p-values, confidence intervals) for performance comparisons.
- **Our Response**: As per your suggestion, we have now incorporated statistical significance testing for all performance comparison results to provide a more rigorous evaluation.
- **Revised Manuscript**:
  - [Table 2 and 3 of main text, All tables of Supplementary Materials F and G] *For all performance comparison metrics (e.g., AUROC, Accuracy), we have added 95% confidence intervals. In cases where there is a statistically significant difference between models, we have now explicitly reported the p-value.*

---

6. Comment about word usage of 'Lifespan' and 'Precision psychiatry'

- **Reviewer's Comment**: The manuscript makes strong claims about "lifespan-inclusive" or "precision psychiatry" applications. However, no elderly or neurodegenerative datasets were used. Such claims should be moderated unless validated with such cohorts.
- **Our Response**: We thank the reviewer for this important and constructive feedback. We agree that our original claims regarding "lifespan-inclusive" applications and "precision psychiatry" were too broad for the scope of the cohorts used in our study. We acknowledge that the absence of elderly or neurodegenerative disease datasets limits the generalizability of our findings. Accordingly, we have carefully revised the manuscript to moderate our claims and explicitly state these limitations. The specific changes are as follows:
- **Revised Manuscript**:
  - [Abstract] *"By integrating scale-aware neural dynamics with deep learning, MBBN delivers more accurate and interpretable biomarkers, opening avenues for developing more precise psychiatric biomarkers and advancing developmental neuroscience."*
  - [Discussion paragraph 6] *"Unlike many existing models that are validated only on narrow demographics, MBBN successfully generalized across developmental stages, revealing its potential as a neuroimaging tool with strong generalizability across child, adolescent, and adult cohorts."*

- [Discussion paragraph 12] *"Finally, while the model proved robust across cohorts with heterogeneous acquisition parameters, further validation on more diverse populations, including elderly and neurodegenerative cohorts,*
- *is required."*
- [Discussion paragraph 13] *"Equally important will be validating MBBN's applicability across diverse patient populations to move toward broader clinical utility."*
- [Discussion paragraph 14] *"By integrating neurophysiological principles with transformer architectures, it offers both predictive power and mechanistic insights, laying a solid foundation for future data-driven applications in clinical and developmental neuroscience."*

---

7. Comment about insufficient explanation of terminologies

- **Reviewer's Comment**: Many terms (e.g., "CLS token," "communicability masking," "spatial loss") are introduced without sufficient explanation. For Communications Biology, a broader audience explanation is needed.
- **Our Response**: We sincerely thank the reviewer for this excellent suggestion to improve the manuscript's accessibility for a broader audience. We agree that several technical terms were not sufficiently explained for readers who may not be experts in deep learning or network science. To address this, we have undertaken a comprehensive revision of several method sections to enhance clarity:
- **Revised Manuscript**:
  - [Method 3.7.2 paragraph 3 : CLS token] *"To derive a single, task-relevant representation from the entire fMRI time series, we utilize the special [CLS] token, a standard component of the BERT architecture. This token is prepended to the input sequence and is designed to aggregate information across all time points during the model's encoding process. Conceptually, the [CLS] token functions like an executive summary of a detailed report. While the model processes the entire sequence (the full report), it learns to distill the most critical information for the downstream classification task into the final hidden state of this single token. The classifier then uses this condensed summary vector to make its prediction (e.g., diagnosing ADHD), providing an efficient and powerful mechanism for sequence-level classification."*
  - [Method 3.8.2 paragraph 1 : spatial loss] *"Conceptually, this loss function can be analogized to a rule that rewards a cartographer for creating three different, specialized maps of a city (e.g., a traffic map, a zoning map, and a topographical map) from the same satellite data, rather than three nearly identical general maps."*

- [Method 3.9.1 paragraph 2 : communicability masking] *“Conceptually, our masking strategy can be analogized to understanding a social network by temporarily hiding its most central 'influencers' and inferring their roles based on the activity of their connections. By masking these influential nodes during pretraining, we force the model to learn their functional roles through contextual inference, similar to masked language modeling but tailored to brain network topology.”*

---

8. Comment about efficiency of the model

- **Reviewer’s Comment**: The model uses multiple transformer modules and custom losses. Pretraining spans 1000 epochs. The authors should discuss: model efficiency and feasibility for labs without high-end computing resources. And possible simplifications or distillations of the model for practical use.
- **Our Response**: We thank the reviewer for this very practical and important suggestion regarding the model's efficiency and accessibility. We agree that this is a crucial point for translating our work into a widely usable tool. Accordingly, we have expanded the final paragraphs of our Discussion section to thoroughly address these considerations.
- **Revised Manuscript**:
    - [Discussion paragraph 13] *“To mitigate the significant computational cost of pretraining, we are releasing our trained models to the public, allowing researchers with limited infrastructure to bypass this intensive step and apply our framework directly via efficient fine-tuning. Furthermore, we plan to develop more lightweight versions of MBBN through architectural simplification and knowledge distillation, creating student models suitable for resource-constrained clinical settings.”*

General Revision

During final proofreading, we discovered a calculation error in the percentage improvement reported for ASD classification. The improvement was originally stated as approximately 50%, but the corrected value is approximately 40%. Importantly, the corrected numbers remain consistent with our main claim of superior performance over all baselines and do not alter the overall ranking or qualitative conclusions of the study.

References

Grinsztajn, L., Oyallon, E., & Varoquaux, G. (2022). Why do tree-based models still outperform deep learning on typical tabular data?. *Advances in neural information processing systems*, *35*, 507-520.

Dear Reviewer 2,

We sincerely thank you for your in-depth analysis and constructive feedback on our manuscript. The points you have raised are crucial for enhancing the robustness and interpretability of our model, and we believe they are key to elevating the quality of our paper to the next level. We provide below our detailed responses and the corresponding revisions for all the issues you have identified.

1. Comment about robustness of frequency-dividing

- **Reviewer's Comment**: How sensitive are the frequencies f_1 and f_2 to age, sex, head motion, and multi-site acquisition differences? Could you provide an age-matched subsample analysis or motion-corrected re-estimation to ensure that the reported disorder effects are not driven by these confounds?
- **Our Response**: We thank the reviewer for this excellent suggestion to rigorously test for potential confounding effects. To address this, we performed a two-part analysis using the ABCD cohort, with full details now included in the main text (Results 2.3) and Supplementary Materials (Section E). First, we directly tested for associations between the knee frequencies (f_1, f_2) and confounds. We found that age and head motion were not significantly associated, while sex and acquisition sites showed some expected effects. Second, to ensure our disorder effects were not driven by these factors, we constructed a propensity score-matched subsample to balance all covariates. On this stringently controlled dataset, the ADHD classification performance remained robust and even slightly increased, confirming that the disorder signal persists after accounting for these confounds. We believe these new analyses substantially strengthen our findings, and we are grateful for the recommendation.
- **Revised Manuscript**:
    - [Method section 3.5 whole paragraph] *"Associations between knee frequencies (f1, f2) and sex, age, head motion (mean FD), and site (22 levels) were assessed using regression/AN(C)OVA with site modeled as a factor; multiplicity-adjusted p-values are reported in Supplementary Materials E. For sensitivity, we performed 1:1 nearest-neighbor propensity score matching (logistic model; covariates: sex, age, mean FD, site; caliper = 0.2 SD of the logit; no replacement) to create a confound-balanced subsample. Balance was confirmed by standardized mean differences < 0.10 for all matched covariates. Classification AUROC was then compared before and after matching using a paired t-test."*
    - [Result section 2.3 whole paragraph] *"Using ABCD, we tested associations between knee frequencies and major confounds. Sex was associated with f1 only (not f2), suggesting a potential sex-linked physiological contribution at lower frequencies, whereas site effects were present for both knees (expected under 22-site acquisition heterogeneity). By contrast, age and head*

motion (mean FD) were not significantly related to either knee. To ensure that disorder effects were not driven by these factors, we constructed a propensity score–matched subsample jointly balancing sex, age, motion, and site; covariates were well balanced (standardized mean differences < 0.10). After propensity score matching, AUROC increased from 0.633 to 0.651; however, the difference did not reach statistical significance under our paired comparison (p = 0.3742). Full association tables and balance diagnostics are provided in the Supplementary Materials E."

- [Supplementary section E whole sections] *"We assessed whether major potential confounds—age, sex, head motion (mean FD), and multi-site acquisition—could bias the reported effects in the ABCD cohort. We (i) compared group characteristics before and after propensity score matching (PSM) and (ii) tested associations between the learned knee frequencies (f1, f2) and each confound."*

---

2. Comment about test-retest reliability of biomarkers

- **Reviewer's Comment**: The paper reports test-set AUROCs but not test-retest reliability. Could you compute the intraclass correlation (ICC) or Pearson r of the frequency-specific attention matrices for the 40 UKB subjects with repeat scans to demonstrate temporal stability of the biomarkers?
- **Our Response**: We thank the reviewer for this valuable suggestion to assess the temporal stability of our biomarkers. While we did not have access to the UKB repeat scans, we have now performed a test-retest reliability analysis using the Human Connectome Project (HCP) dataset as a well-established proxy. The results demonstrated excellent reliability, with the Intraclass Correlation Coefficient (ICC) for the frequency-specific attention matrices consistently exceeding 0.95. This confirms that our derived biomarkers are highly stable over time. We have added a detailed account of this analysis in a new section of the Supplementary Materials (Appendix D). Your feedback has been instrumental in strengthening our manuscript.
- **Revised Manuscript**:
  - [Supplementary Materials D whole paragraphs] *"To assess the temporal stability of our model's derived biomarkers, we performed a test-retest reliability analysis on the frequency-specific attention matrices. Due to the unavailability of UK Biobank test-retest data, we utilized the resting-state fMRI data from the Human Connectome Project (HCP) dataset, which includes two separate runs (run 1 and run 2) for each participant, serving as an excellent proxy for evaluating short-term reliability.*

    We fine-tuned the MBBN model on a sex classification task using the HCP dataset. After training, we extracted the subject-level attention matrices for each of the three frequency bands (high, low, ultralow) from both run 1 and run 2 of the test set. To evaluate the consistency of these matrices over time,

we calculated the Intraclass Correlation Coefficient (ICC), using a two-way mixed-effects model for absolute agreement (ICC(A,1)). The analysis was conducted separately for male and female subjects to ensure reliability was not sex-dependent.

The results demonstrated high to excellent test-retest reliability across all conditions. For both male and female participants, the ICC values for the attention matrices in the high, low, and ultralow frequency bands were all greater than 0.95. This high level of temporal stability confirms that the frequency-specific connectivity patterns captured by MBBN are robust and consistent, strengthening their validity as reliable biomarkers for downstream clinical and cognitive prediction tasks."

---

3. Comment of hyperparameter (λ) setting of spatial loss

- **Reviewer's Comment**: The spatial loss λ is tuned per dataset (1–100). Can you provide a principled heuristic—perhaps based on the ratio of task loss gradient norms to spatial loss gradient norms—to set λ automatically, and does this heuristic generalize to new cohorts without grid-search?
- **Our Response**: We thank the reviewer for this expert-level suggestion. The idea of using a principled heuristic like gradient norm balancing to automatically set λ is an excellent one for improving generalizability. Following your recommendation, we implemented and tested such a dynamic weighting scheme. Our empirical results, however, showed that this method was highly sensitive to noisy gradients in the early stages of training. As a result, it did not consistently outperform a well-tuned fixed λ in our experiments. We have now added a sentence to the Discussion section to explicitly mention this valuable experiment and its outcome. We appreciate you pushing us to test this advanced technique, as it has added important context to our methodological choices.
- **Revised Manuscript**:
    - [Discussion paragraph 12] *"Third, the spatial loss weight λ was tuned via grid-search. While a more principled approach would be a dynamic weighting scheme, our experiments with a gradient-norm-based method showed sensitivity to noisy initial gradients and did not consistently outperform a well-tuned fixed λ. "*

---

4. Comment about task-evoked fMRI data

- **Reviewer's Comment**: The current work uses resting-state data. How would MBBN handle task-evoked fMRI where the power-law assumption may be transiently violated?

- **Our Response**: We thank the reviewer for this excellent and forward-looking question. It is a crucial point regarding the generalizability of our framework to task-evoked fMRI, and we have expanded our Discussion section to address it. We agree that the scale-free assumption for our *frequency decomposition* is most stable at rest. However, we hypothesize that the model's core Multi-Band Attention mechanism is inherently well-suited to capture the transient, task-driven neural dynamics you describe. For instance, the model could learn to classify cognitive states by detecting task-related power shifts within specific frequency bands. We are grateful for this insightful question, which has helped us clarify the future scope and potential of our framework in the manuscript.
- **Revised Manuscript**:
  - [Discussion paragraph 13] *"While the scale-free assumption for our frequency decomposition is most stable during rest, we hypothesize that the Multi-Band Attention mechanism is inherently well-suited to capture transient, task-driven neural dynamics. For instance, the model could learn to detect task-related power shifts in specific frequency bands—such as increased high-frequency activity in the frontoparietal network during a working memory task—to classify cognitive states and extract dynamic, task-evoked connectivity."*

General Revision

During final proofreading, we discovered a calculation error in the percentage improvement reported for ASD classification. The improvement was originally stated as approximately 50%, but the corrected value is approximately 40%. Importantly, the corrected numbers remain consistent with our main claim of superior performance over all baselines and do not alter the overall ranking or qualitative conclusions of the study.

Reviewer 3

Dear Reviewer 3,

Thank you very much for your insightful review and constructive criticism of our manuscript. The issues you pointed out regarding the overall organization, clarity, and methodological rigor were significant weaknesses in our paper, and addressing them has greatly improved the quality of our research. Based on your feedback, we have substantially revised the manuscript. Please find our detailed responses and a description of the corresponding revisions for each of your points below.

1. Comment about selection of tasks

- **Reviewer's Comment**: A primary concern is the lack of clarity regarding the selection criteria for tasks used in the analyses. For instance, in the ABCD dataset, depressive symptom scores are available and could be utilized for regression analyses, yet these are not addressed. Similarly, in the ABIDE dataset, clinically relevant behavioral metrics such as the Social Responsiveness Scale (SRS) and Autism Diagnostic Observation Schedule (ADOS) scores are available, but their exclusion is neither justified nor explained.
- **Our Response**: We thank the reviewer for this important point regarding the clarity of our task selection criteria. We agree this was not sufficiently detailed and have now added a subsection to the Methods (Section 3.2) to provide our explicit rationale. In this new section, we explain that for the ABCD dataset, our goal was to test the model's utility in a categorical diagnostic context (disorder vs. controls), which aligns with common clinical applications. For the ABIDE dataset, we prioritized the primary diagnostic label over continuous metrics like SRS/ADOS to ensure maximum data standardization and reliability, as behavioral scores can have significant inter-site variability across the consortium. We believe this clarification significantly improves the manuscript, and we thank you for the suggestion.
- **Revised Manuscript**:
    - [Method section 3.2 paragraph 7, 8, 9] *"Our selection of downstream prediction tasks was guided by the prioritization of well-established phenotypes that ensure large sample sizes, minimal missing data, and high clinical relevance for classification frameworks.*

      For the ABCD dataset, we focused on a binary classification of depression (disorder vs. controls) rather than regression on continuous symptom scores. This choice was motivated by the goal of testing the model's utility in a categorical diagnostic context, which aligns with common clinical applications and provides a clear, robust target for classification models.

      For the ABIDE dataset, we similarly prioritized the primary diagnostic label (ASD vs. typically developing controls) over continuous behavioral metrics such as the Social Responsiveness Scale (SRS) or Autism Diagnostic Observation Schedule (ADOS). This decision was driven by the need for maximum data standardization and reliability. The primary diagnosis is the most consistently harmonized variable across the many different data

collection sites within the ABIDE consortium, whereas behavioral scores can be subject to significant inter-site variability in administration and scoring."

---

2. Comments about structure of result section

- **Reviewer's Comment**: The results section is poorly structured, requiring readers to frequently cross-reference earlier sections to understand the definitions and findings. Critical information—such as the specific fluid intelligence metric used and the handling of missing (NaN) assessments—is not clearly introduced or described, which significantly hampers interpretability.

- **Our Response**: We sincerely thank the reviewer for this invaluable feedback on the structure and clarity of the Results section. We agree that the original presentation was not self-contained and required excessive cross-referencing. To address these concerns, we have made two major improvements. First, we have added a new introductory subsection, 'Experimental Setup and Downstream Tasks' (now Section 2.1), at the beginning of the Results. This section now explicitly defines all predictive tasks—clarifying, for instance, that Fluid Intelligence scores are derived from the NIH Toolbox and specifying the PHQ-9 and CBCL metrics used. It also clearly states our procedure for handling missing assessments (listwise exclusion) and the evaluation metrics (AUROC/MAE) for all analyses. Second, as you suggested, we have reorganized the subsequent subsections to follow a more logical narrative, guiding the reader from the general performance overview to the specific neurobiological interpretations. We are confident that these changes make the Results section significantly more interpretable and self-contained. Thank you again for this constructive guidance.

- **Revised Manuscript**:

    - [Method section 2.1, whole paragraphs] *"To rigorously evaluate the performance and generalizability of MBBN, we designed a diverse set of predictive tasks spanning biological, cognitive, and clinical domains across three large-scale cohorts (UKB, ABCD, and ABIDE).*

      The tasks were defined as follows. For biological phenotypes, we performed sex classification. For cognitive function, we predicted Fluid Intelligence scores derived from the NIH Toolbox. In the clinical domain, we predicted a variety of psychiatric outcomes: (1) depression diagnosis in the ABCD dataset (classification), (2) Patient Health Questionnaire-9 (PHQ-9) depression scores in the UKB dataset (regression), (3) ADHD diagnosis based on a Child Behavior Checklist (CBCL) T-score exceeding 65 in the ABCD dataset (classification), and (4) Autism Spectrum Disorder (ASD) diagnosis in the ABIDE dataset (classification).

      For each downstream task, participants with missing or invalid assessment scores were excluded from the analysis (listwise exclusion). Model performance was evaluated using the Area Under the Receiver Operating

Characteristic curve (AUROC) for all classification tasks and the Mean Absolute Error (MAE) for all regression tasks."

  - Improved Narrative Flow: The subsequent subsections are now organized to guide the reader logically from the general performance overview to the specific contributions of our pretraining strategy, and finally to the interpretable neurobiological findings.

---

3. Comment about limitation of quantitative findings

- **Reviewer's Comment**: The quantitative findings are limited to Table 1 and Figures 2 and 3, which is insufficient for a study of this scope. Moreover, Table 1 is not cited or discussed in the main text, and no statistical comparisons (e.g., significance testing) are provided for the differences observed in Figure 2. This omission raises concerns regarding the robustness and interpretability of the reported results.

- **Our Response**: We fully agree with the reviewer's comment and acknowledge that the lack of detailed quantitative results and statistical comparisons was a significant weakness. To thoroughly address this, we have made several key improvements to the manuscript.

  1. Statistical Significance Testing: We have now performed statistical comparisons for all performance results. As shown in the revised Figure 2 and Table 1, we have added p-values from paired t-tests to formally evaluate the differences between MBBN and baseline models.

  2. Robustness and Precision: To better illustrate the robustness of our findings, we have incorporated 95% confidence intervals for all reported metrics throughout the main text and Supplementary Materials (e.g., Appendices F and G) .

  3. Citation and Discussion: We have corrected the oversight regarding Table 1, which is now explicitly cited and discussed in the main text of the Results section (Section 2.2).

  We are confident that these extensive additions have substantially improved the quantitative rigor and interpretability of our study. We are very grateful for your feedback, which prompted these critical improvements.

- **Revised Manuscript**:

  - [Result section 2.2, paragraph 1] *"The primary results presented in Figure 2 A-F and Table 1 focus on experiments conducted with the HCP-MMP1 atlas; corresponding analyses using the Schaefer atlas are detailed in the Supplementary Materials G.1."*

  - [Table 2 and 3 of main text, All tables of Supplementary Materials F and G] *We have now included statistical significance tests (p-values) for all*

performance comparison results presented in Figure 2. Furthermore, we have incorporated 95% confidence intervals to reinforce the robustness of our findings.

---

4. Comment about clarity of visualization

- **Reviewer's Comment**: Figure 3 attempts to illustrate connectivity alterations associated with ASD and ADHD; however, it is not effective in conveying spatial information. Despite the accompanying textual description, the figure lacks anatomical clarity and does not allow readers to easily localize the abnormalities. Furthermore, it is unclear why only ASD and ADHD are shown, while other regression outcomes are omitted without explanation.

- **Our Response**: Thank you for your constructive feedback. We agree that the original Figure 3 lacked sufficient anatomical clarity and that our rationale for focusing only on ASD and ADHD was not adequately explained. We have addressed both of these points in the revised manuscript.

    1. Improving Anatomical Clarity: We have substantially revised Figure 3 to enhance its interpretability. Key hub regions are now explicitly labeled within the brain images, and the visualization has been updated for easier and more precise localization of connectivity alterations. The corresponding anatomical names are also now clearly stated in the figure caption.

    2. Justifying Task Selection: To clarify our focus, we have added a new sentence to the text (now in Section 2.5). We explain that we chose to highlight ADHD and ASD because they are the disorders for which our model achieved its highest classification performance, thus providing the most clear and effective demonstration of its interpretability.

    We are confident that these changes make our neurobiological findings much easier to follow and interpret. We thank you for your guidance.

- **Revised Manuscript**:

    - [Figure 3] We have substantially revised Figure 3 to enhance its anatomical clarity. Key hub regions are now explicitly labeled within the figure. The visualization technique has been updated to ensure that brain areas with significant connectivity alterations are easily and precisely identifiable. Furthermore, the corresponding anatomical names of these hub regions are now clearly stated in the figure caption and the main text.

    - [Method section 2.5 paragraph 1] *"Figure 3 focuses specifically on ADHD and ASD because these are the representative psychiatric disorders for which our model achieved the highest classification performance, thereby providing a clear and effective demonstration of the model's interpretability."*

---

5. Comment about improvement rates

- **Reviewer's Comment**: The manuscript emphasizes improvement rates as a key performance metric. However, in large-scale datasets such as UKB and ABCD, effect sizes in regression tasks are known to be small (e.g., Cohen's d ≈ 0.2). An apparent 10% improvement may correspond to a trivial increase in correlation (e.g., from 0.10 to 0.11), which has limited clinical or practical significance. The authors should provide a more nuanced discussion of these limitations.

- **Our Response**: We thank the reviewer for this thoughtful and important point regarding the interpretation of practical significance. We fully agree that relative improvement rates must be contextualized, especially in domains where effect sizes for regression tasks are known to be modest. To address this, we have added a new paragraph to our Discussion (Paragraph 13) that provides a more nuanced positioning of our contributions.

  In this revision, we now explicitly:

  - Acknowledge that MBBN's advantage is less pronounced for regression targets and frame our core contribution as methodological: a new framework providing a foundation for future models.
  - Contrast this with the model's strong and practically relevant performance on categorical phenotypes, highlighting the high AUROCs achieved in clinical differentiation tasks (e.g., 0.980 for UKB sex classification, 0.810 for ABIDE ASD classification).
  - Clarify our overall positioning: MBBN is a methodological advance that delivers immediate high utility for classification, while providing a principled basis for future work aimed at improving regression performance.

  We believe this revised discussion now more accurately frames the significance of our results. We are grateful for your feedback, which has helped us to better articulate the specific strengths and current limitations of our framework.

- **Revised Manuscript**:

  - We would like to clarify that our manuscript already contained a discussion on the potential source of this limitation. As the reviewer correctly identified this as a critical point, we have further emphasized in our Discussion section (*"Second, a key limitation pertains to the model's modest performance on regression tasks, which may arise from the intrinsic bias of transformers that tend to learn non-smooth functions [76]"*)
  - [Discussion paragraph 13] *"While our evaluation shows that MBBN's advantage is less pronounced for regression targets, we emphasize that our core contribution is methodological: a frequency-resolved, biologically grounded framework that can serve as a foundation for future models to better capture continuous outcomes. At the same time, MBBN exhibits strong and practically relevant performance on categorical phenotypes, achieving*

high AUROCs (e.g., sex classification in ABCD: 0.916; UKB: 0.980; ASD classification in ABIDE: 0.810). This balance clarifies our positioning: MBBN is a methodological advance that already delivers high utility for clinical differentiation tasks, while providing a principled basis for subsequent extensions aimed at improving regression performance."

---

6. Comment about inconsistency between preprocessing and filtering

- **Reviewer's Comment**: Given that the proposed framework aims to capture frequency-specific information, it is surprising that temporal filtering was applied during preprocessing. More critically, the filtering parameters differ across datasets, which is methodologically inconsistent and undermines the comparability of results across datasets.

- **Our Response** : We thank the reviewer for raising this important point. We have now clarified our rationale for using pre-filtered data in the Methods section (3.1). Our decision to use the consortium-recommended preprocessing pipelines was a deliberate choice to benchmark MBBN's performance on the most widely used and validated versions of these datasets. This ensures our results are comparable and relevant to the broader field. We agree with the reviewer that this is a key aspect of our methodology, and we have now added a statement to the Discussion section clarifying that our framework was designed to identify novel frequency-specific patterns *within* these standard preprocessed signals, rather than from the raw unfiltered data. Crucially, our results show that this approach is highly effective. As detailed in our ablation study (Supplementary Table F8), MBBN's data-driven approach to frequency decomposition provides a significant performance advantage over methods using coarser, fixed bands on the same filtered data. This demonstrates the model's strength in revealing nuanced spatiotemporal dynamics that are missed by other methods, even within a standard preprocessing framework.

- **Revised Manuscript**:

    - [Method section 3.1, paragraph 1] *"The ABCD, UKB, and ABIDE datasets underwent dataset-specific preprocessing pipelines to honor the official protocols and best practices established by each respective consortium. In ABCD, a conservative bandpass filter (0.009-0.08 Hz) was applied following consortium protocols for adolescent populations ([20]). UKB employed a broader filter (0.008-0.1 Hz) optimized for adult populations to minimize heart rate variability effects ([21]). ABIDE used finite impulse response filtering (0.01-0.1 Hz) specifically designed for multi-site ASD data integration ([22])."*

    - *[Discussion section, paragraph 13] "Furthermore, our frequency decomposition operates on standard, pre-filtered fMRI signals to ensure comparability with established research. Consequently, the model identifies meaningful sub-bands within this conventional spectral*

window, not from the complete raw signal, a scope that future work could expand upon."

---

7. Comment about distortion correction and TR

- **Reviewer's Comment**: The authors do not address whether distortion correction was applied for the UKB and ABCD datasets. Furthermore, the ABIDE dataset has a significantly different temporal resolution compared to UKB and ABCD. The potential influence of repetition time (TR) and other acquisition parameters on the proposed method is neither explored nor discussed.

- **Our Response**: We thank the reviewer for raising these important points about our methodological details. We have now revised the manuscript to provide greater clarity on both preprocessing and the handling of heterogeneous acquisition parameters.

  1. Distortion Correction: To address your first point, we have clarified in the Methods section (Subsection 3.1) that susceptibility distortion correction was indeed applied to all datasets as part of our fMRIPrep pipeline.

  2. Acquisition Parameter Heterogeneity: To address your second point, we have made two additions. First, we included Table 3 in the Methods section to transparently list the different acquisition parameters (including TR) for each cohort. Second, we added a sentence to the Discussion section acknowledging that our model proved robust across these heterogeneous parameters, while also noting that further validation is a valuable direction for future work.

  We are grateful for your feedback, which has helped us to significantly improve the transparency and completeness of our methodological description.

- **Revised Manuscript**:

  - [Method section 3.1, paragraph 2] *"The following preprocessing procedures were applied to each participant's resting-state BOLD data: First, skull-stripping, slice-timing correction, susceptibility distortion correction, and spatial normalization to MNI space were conducted using fMRIPrep ([23]). Subsequently, head motion artifacts and signals from white matter were removed using component-based noise correction (CompCor) ([24])."*

  - [Method section 3.2, Table 3] *"Demographic and diagnosis distribution details and neuroimaging parameter information for the three datasets (UKB, ABCD, ABIDE) are summarized in Table 2, 3."*

  - [Discussion paragraph 12] *"Finally, while the model proved robust across cohorts with heterogeneous acquisition parameters, further validation on more diverse populations, including elderly and neurodegenerative cohorts, is required."*

8. Comment about dataset explanation and limitations on selection criteria

- **Reviewer's Comment**: The manuscript lacks a clear description of the datasets used. The reported sample sizes differ from those in previous studies, yet no rationale is provided for these discrepancies. For example, it is unclear whether the ABCD analysis uses baseline data, and why approximately 2,000 participants appear to have been excluded. For the ABIDE dataset, it is not specified whether ABIDE-I or ABIDE-II was used, whether data from a single site was selected, or what criteria guided these decisions.

- **Our Response**: We thank the reviewer for highlighting the lack of clarity regarding our dataset descriptions and subject selection criteria. We agree that this information is critical for the manuscript's transparency and reproducibility. To address this thoroughly, we have added a new, dedicated subsection to our Methods titled '3.2 Dataset Description and Subject Selection Criteria.'

  This new section now provides the specific details you requested:

  - For the ABCD dataset, we clarify that we used the baseline data and provide the three explicit exclusion criteria (e.g., fMRI QC failure, incidental findings, missing data) that account for our final sample size.

  - For the ABIDE dataset, we now specify that we used the ABIDE-I cohort, included data from all available sites to maximize heterogeneity, and detail the exclusion criteria based on quality control and diagnostic information completeness.

  We are confident that these additions make our methodology far more transparent and reproducible. We are very grateful for your detailed feedback.

- **Revised Manuscript**:

  - [Method section 3.2. paragraph 1-4] *"This study utilized three large-scale, publicly available datasets: the UK Biobank (UKB), the Adolescent Brain Cognitive Development (ABCD) Study, and the Autism Brain Imaging Data Exchange (ABIDE). The final sample size for each cohort was determined following a rigorous quality control and subject selection procedure.*

    UK Biobank (UKB): The UKB dataset is a prospective cohort study of approximately 500,000 adults. For our study, we included participants who had available resting-state fMRI data and passed the quality control assessments provided by the UKB consortium, resulting in a final sample of 41,283 individuals.

    Adolescent Brain Cognitive Development (ABCD) Study: We used the baseline, year-one data from the ABCD Study, a longitudinal study of brain development in U.S. children. The initial release contained imaging data from over 11,000 participants. Our final sample of 8,833 participants was derived

after excluding subjects based on several criteria: (1) failure to pass the fMRI quality control protocols provided by the ABCD consortium (e.g., due to excessive head motion), (2) presence of incidental neurological findings, and (3) unavailability of relevant behavioral or diagnostic data for our downstream tasks.

Autism Brain Imaging Data Exchange (ABIDE): We used data from the ABIDE-I cohort, which aggregates resting-state fMRI data from 17 international acquisition sites. We included all sites to maximize sample size and heterogeneity. Subjects were excluded if they failed the quality control assessments from the Preprocessed Connectomes Project (PCP) or if their diagnostic information was incomplete. This resulted in a final sample of 141 participants for the ASD classification task."

General Revision

During final proofreading, we discovered a calculation error in the percentage improvement reported for ASD classification. The improvement was originally stated as approximately 50%, but the corrected value is approximately 40%. Importantly, the corrected numbers remain consistent with our main claim of superior performance over all baselines and do not alter the overall ranking or qualitative conclusions of the study.

Our Response

We thank the reviewer for highlighting the importance of transparent and numerically consistent reporting. We have now (i) added a stepwise participant flow diagram, (ii) revised Table 2 to clearly distinguish initial and task-specific analytic samples, and (iii) performed a full cross-check to ensure consistency across the manuscript and Supplementary Materials.

Revised Manuscript

To address the reviewer's concern about sample size inconsistencies and the transparency of participant selection and attrition procedures, we have revised the Method section 3.2 and Table 2, and added Figure 4.

In response to the reviewer's request for clearer reporting of cohort construction, we provide below a detailed description of participant selection, quality control, and exclusion criteria for each dataset.

[Method section 3.2 paragraph 2-5]

Figure 4 summarizes the stepwise subject selection and attrition procedure for each dataset, including imaging availability, quality control, preprocessing success, and phenotype completeness. All sample sizes reported in the text, tables, and supplementary materials were cross-checked for numerical consistency.

UK Biobank (UKB): The UKB dataset is a prospective cohort study of approximately 500,000 adults. For our study, we included participants who had available resting-state fMRI data. Subjects were excluded if preprocessing failed due to spatial incompatibility or empty brain masks, or if invalid ROI signals were detected after atlas-based extraction (e.g., all-zero time series). Participants without required metadata were also excluded. These procedures resulted in a final analytic sample of 40,699 individuals.

Adolescent Brain Cognitive Development (ABCD) Study: We used the baseline, year-one data from the ABCD Study, a longitudinal study of brain development in U.S. children. The initial release contained imaging data from 11,604 participants. All subjects included in the present study passed the standardized MRI quality control procedures provided by the ABCD consortium. Additional exclusions were applied if preprocessing failed due to spatial incompatibility or empty brain masks, or if invalid ROI signals were identified after atlas-based extraction (e.g., all-zero time series). Participants without relevant behavioral or diagnostic data required for downstream tasks were also excluded. These criteria yielded a final analytic sample of 8,833 participants.

Autism Brain Imaging Data Exchange (ABIDE): We used data from the ABIDE-I cohort, which aggregates resting-state fMRI data from 17 international acquisition sites. All sites were included to maximize sample heterogeneity. Subjects were excluded if preprocessing failed due to spatial incompatibility or empty brain masks, if atlas-based ROI time series extraction resulted in invalid signals (e.g., all-zero time series), or if diagnostic metadata were incomplete. Quality control information from the Preprocessed Connectomes Project (PCP) was used as an initial reference. After applying these criteria uniformly across subjects, the final analytic sample consisted of 141 participants for the ASD classification task.
